# Viruses Roll the Dice: The Stochastic Behavior of Viral Genome Molecules Accelerates Viral Adaptation at the Cell and Tissue Levels

**DOI:** 10.1371/journal.pbio.1002094

**Published:** 2015-03-17

**Authors:** Shuhei Miyashita, Kazuhiro Ishibashi, Hirohisa Kishino, Masayuki Ishikawa

**Affiliations:** 1 Precursory Research for Embryonic Science and Technology (PRESTO), Japan Science and Technology Agency, Kawaguchi, Japan; 2 Plant-Microbe Interactions Research Unit, Division of Plant Sciences, National Institute of Agrobiological Sciences, Tsukuba, Japan; 3 Graduate School of Agricultural and Life Sciences, University of Tokyo, Tokyo, Japan; University of Wisconsin-Madison, UNITED STATES

## Abstract

Recent studies on evolutionarily distant viral groups have shown that the number of viral genomes that establish cell infection after cell-to-cell transmission is unexpectedly small (1–20 genomes). This aspect of viral infection appears to be important for the adaptation and survival of viruses. To clarify how the number of viral genomes that establish cell infection is determined, we developed a simulation model of cell infection for tomato mosaic virus (ToMV), a positive-strand RNA virus. The model showed that stochastic processes that govern the replication or degradation of individual genomes result in the infection by a small number of genomes, while a large number of infectious genomes are introduced in the cell. It also predicted two interesting characteristics regarding cell infection patterns: stochastic variation among cells in the number of viral genomes that establish infection and stochastic inequality in the accumulation of their progenies in each cell. Both characteristics were validated experimentally by inoculating tobacco cells with a library of nucleotide sequence–tagged ToMV and analyzing the viral genomes that accumulated in each cell using a high-throughput sequencer. An additional simulation model revealed that these two characteristics enhance selection during tissue infection. The cell infection model also predicted a mechanism that enhances selection at the cellular level: a small difference in the replication abilities of coinfected variants results in a large difference in individual accumulation via the multiple-round formation of the replication complex (i.e., the replication machinery). Importantly, this predicted effect was observed in vivo. The cell infection model was robust to changes in the parameter values, suggesting that other viruses could adopt similar adaptation mechanisms. Taken together, these data reveal a comprehensive picture of viral infection processes including replication, cell-to-cell transmission, and evolution, which are based on the stochastic behavior of the viral genome molecules in each cell.

## Abstract 2

Simulation models of cell-to-cell viral transmission illustrate how stochastic processes occurring within individual infected cells form a sophisticated adaptation system that optimizes viruses for further infection.

## Author Summary

Viruses rapidly adapt to environmental changes, complicating our efforts to control the spread of viral infections. Adaptation occurs in two steps: the generation of adaptive variants by random mutations and the subsequent selection of those adaptive variants. High mutation rates in viruses have been well documented; however, little is known about how adaptive variants are rapidly selected, especially given that most viral gene products are shared among viral populations within an infected cell, thereby diluting the direct benefits of each variant. Here we used an RNA virus—tomato mosaic virus—to examine the selection mechanisms hidden in its infection processes. First, we simulated the cell infection process computationally. We found that stochastic behaviors of viral genome molecules result in characteristic patterns of offspring accumulation within cells: (i) predominant replication of offspring from only a handful of founder viral genome molecules in each cell, (ii) variation in the number of offspring originating from each founder, and (iii) variation in the number of founders among cells. We then found that these characteristics enable rapid selection of adaptive variants of the virus during parallel and repeated cell infections, i.e., tissue infections. We speculate that similar selection mechanisms could also be employed by other viruses.

## Introduction

Viruses quickly adapt to different environments. Frequent mutations caused by error-prone replication [1,2] and the subsequent selection of adaptive genomes are the essence of viral adaptation. Although considerable attention has been given to the frequency of mutations, comparably little attention has been paid to the selection processes. In this study, we focused on the behavior of viral RNA molecules in a host cell to determine how adaptive genomes are selected at the cellular and tissue levels.

Most plant viruses spread in plant leaf tissues via transmission from infected cells to adjacent uninfected cells through the plasmodesmata, which connects the cytosol of neighboring cells [3]. Cell-to-cell transmission also occurs in many animal viruses, including human immunodeficiency virus (HIV), herpes simplex virus (HSV), and hepatitis C virus (HCV) [4]. Cell-to-cell transmission could be advantageous for increasing the probability of successful infection by introducing many virus genomes into neighboring uninfected cells [4]. This possibility led to the hypothesis that viral survival becomes more secure when increased numbers of genomes are introduced into neighboring cells. However, as far as investigated, viral cell infection starts by less than 20 founder genomes after cell-to-cell transmission, even though viruses accumulate up to 10^7^ genomes within an infected cell. By analyzing the spatial separation of two differently labeled viral variants from an initially coinfected cell, we demonstrated previously that the plant RNA virus Japanese soil-borne wheat mosaic virus (JSBWMV) infected cells with an average of 5–6 genomes after cell-to-cell transmission [5]. Consistent with these data, a study on two alphaherpesviruses—HSV and pseudorabies virus (PRV)—used a similar method and demonstrated that they infect cells with a mean of 1.4 and 1.6 genomes, respectively [6]. Different analyses also revealed cell infections with small numbers of genomes: HIV formed a mean of 3.82 provirus in a cell (i.e., the viral cDNA integrated into the host genome for replication) after cell-to-cell transmission [7]. Gonzalez-Jara et al. [8] and Gutierrez et al. [9] used a simplified method and suggested that tobacco mosaic virus (TMV) and cauliflower mosaic virus (CaMV) establish cell infections with 1–2 and 2–13 genomes, respectively. The above six viruses belong to four of the seven different Baltimore virus classification groups (JSBWMV and TMV, positive-strand RNA virus; CaMV, pararetrovirus; HSV and PRV, double-strand DNA virus; HIV, retrovirus), suggesting that establishing infection with a small number of genomes after cell-to-cell transmission might be universally advantageous for viruses.

The merit of infecting cells with a small number of genomes after cell-to-cell transmission might be enhancement of adaptation. Error-prone genome replication generates adaptive mutants stochastically, but it generates defective mutants at much higher frequencies [10,11]. Therefore, viruses need to select adaptive genomes and also exclude defective ones from the population. Some gene products or elements on viral genomes act exclusively on the genomes that carry the genes or the elements (i.e., act in *cis*) via certain mechanisms such as the co-translational binding of the gene products to the genome [12]. In contrast, other gene products or elements are shared among intracellular viral population (i.e., act in *trans*). In the case of *cis*-acting genes or elements, viral genomes with “more fit” variations will accumulate faster than those with “less fit” variations, resulting in the selection of the more fit variants. In contrast, in the case of *trans*-acting genes or elements, the sharing of gene products or elements delays selection because even viral genomes with less fit genes or elements will be helped by other genomes in the cell, while viral genomes with more fit genes or elements share the benefit among a large number of viral genomes in the cell (S1 Fig., panel A). Infection with a small number of genomes weakens this constraint, resulting in rapid selection on *trans*-acting genes or elements. Specifically, cell infections with a small number of genomes isolate the adaptive genomes from the defective ones stochastically, which is then followed by selection among intracellular populations (S1 Fig., panel B). Based on this principle, we previously developed a simple simulation model for selection on *trans*-acting genes or elements and showed that infection with <10 genomes results in the rapid selection of adaptive genomes and the exclusion of defective genomes, whereas infection with >50 genomes does not [5]. Therefore, viruses might have evolved to infect cells with a small number of genomes after cell-to-cell transmission to achieve the rapid selection of *trans*-acting genes or elements; however, the mechanisms that determine the number of genomes that establish infection have yet to be elucidated.

In this study, we showed that the plant RNA virus tomato mosaic virus (ToMV) also infects cells with a small number of genomes after cell-to-cell transmission in leaf tissues and after inoculation into protoplasts (i.e., single cells). To clarify how the number of genomes to infect a cell is determined, we developed a simulation model for single-cell infections, taking the stochastic behavior of each viral genome into consideration. We developed an additional simulation model for selection during cell-to-cell spreading. Based on all the data obtained by the simulations and verification experiments, we propose a comprehensive view of the virus infection processes covering replication, cell-to-cell transmission, and evolution.

## Results

### Cell Infections with a Small Number of ToMV Genomes

**Fig 1:**
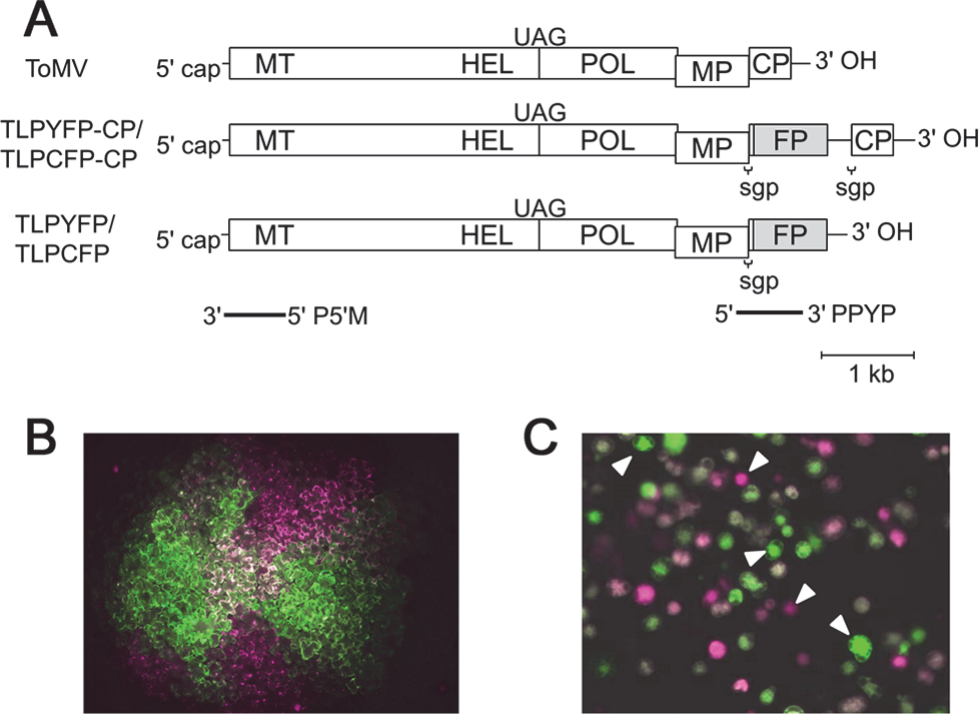
Estimation of founder numbers during cell-to-cell spread in leaf tissues after protoplast infection by ToMV. (A) The genome structure of wild-type ToMV and its derivatives used in the experiments. Rectangles indicate open reading frames (ORFs). MT, a methyltransferase-like domain; HEL, a helicase-like domain; POL, a polymerase-like domain of the replication proteins; MP, the cell-to-cell movement protein; and CP, the coat protein. Translation of the genomic RNA produces two replication proteins, 130K and 180K; 130K contains MT and HEL. The 180K is synthesized via the UAG stop codon read-through of 130K to express POL. FP indicates the fluorescent protein genes (YFP or CFP). TLPYFP-CP and TLPCFP-CP carry the YFP and CFP gene, respectively, between the MP and CP coding regions. The expression of the YFP or CFP genes is driven by a duplicated subgenomic promoter (sgp) for the CP. TLPYFP and TLPCFP lack the CP gene. The regions that correspond to the PPYP and P5′M probes, which were used for northern blotting and RNase protection assays, are indicated by bold lines. (B) An example of spatial separation of the two variants during cell-to-cell spread from a coinfected cell in a tobacco leaf. YFP and CFP fluorescence images were obtained separately 44 hours postinoculation (hpi) using a color charge-coupled device (CCD) camera. After converting the color of the CFP fluorescence image to magenta, the image was merged with the YFP fluorescence image without color conversion. The YFP images look green (rather than yellow) because of the transmission range of the emission filter. Coinfected cells were found at the central part of the infected region as white colored cells. (C) The stochastic occurrence of single infections by one of the two co-inoculated variants in protoplasts. Fluorescence images were obtained at 24 hpi and then processed as described above. White arrowheads indicate singly infected cells.

ToMV is a nonsegmented positive-strand plant RNA virus. We inoculated tobacco leaves with a 1:1 mixture of two ToMV derivatives named TLPCFP-CP and TLPYFP-CP, which carry the cyan fluorescent protein (CFP) and yellow fluorescent protein (YFP) genes, respectively (Fig. 1A). The spread of the two derivatives in tobacco leaves was traced using YFP and CFP fluorescence. Starting from the initially coinfected cells, spatial separations of the derivatives were observed during several cell-to-cell movements (an example is shown in Fig. 1B). This suggests that only a small number of ToMV genomes establish infection after cell-to-cell transmission and that single infections by one of the two derivatives occur stochastically. Analysis of spatial separation patterns indicated that a mean of 3.95 ± 0.27 (mean ± standard error [SE]) genomes establish infection in a cell after the first cell-to-cell transmission from the initially infected cells (See Materials and Methods and [5] for the detailed estimation procedures used). Hereafter, we call the genomes that established an infection in a cell the “founder” and the number of such genomes the “founder number.”

Co-inoculation with the YFP- or CFP-tagged ToMV derivatives TLPYFP and TLPCFP (Fig. 1A) into tobacco protoplasts using electroporation also caused the stochastic occurrence of single infections (Fig. 1C). Analysis of the frequencies of single infections and coinfections after the co-inoculation of 3 μg of each RNA into 1 × 10^6^ protoplasts showed that the mean founder number was 3.94 ± 0.21 (mean ± SE), which was comparable to the infections in the leaf tissues. Here, we assumed that similar mechanisms determine the founder number in protoplast and tissue cell infections after cell-to-cell movement, and we subsequently addressed the mechanism behind this by developing a simulation model for single-cell infections and analyzing the experimental results obtained by protoplast inoculations.

### Development of a Stochastic Model of Single-Cell Infections

**Fig 2:**
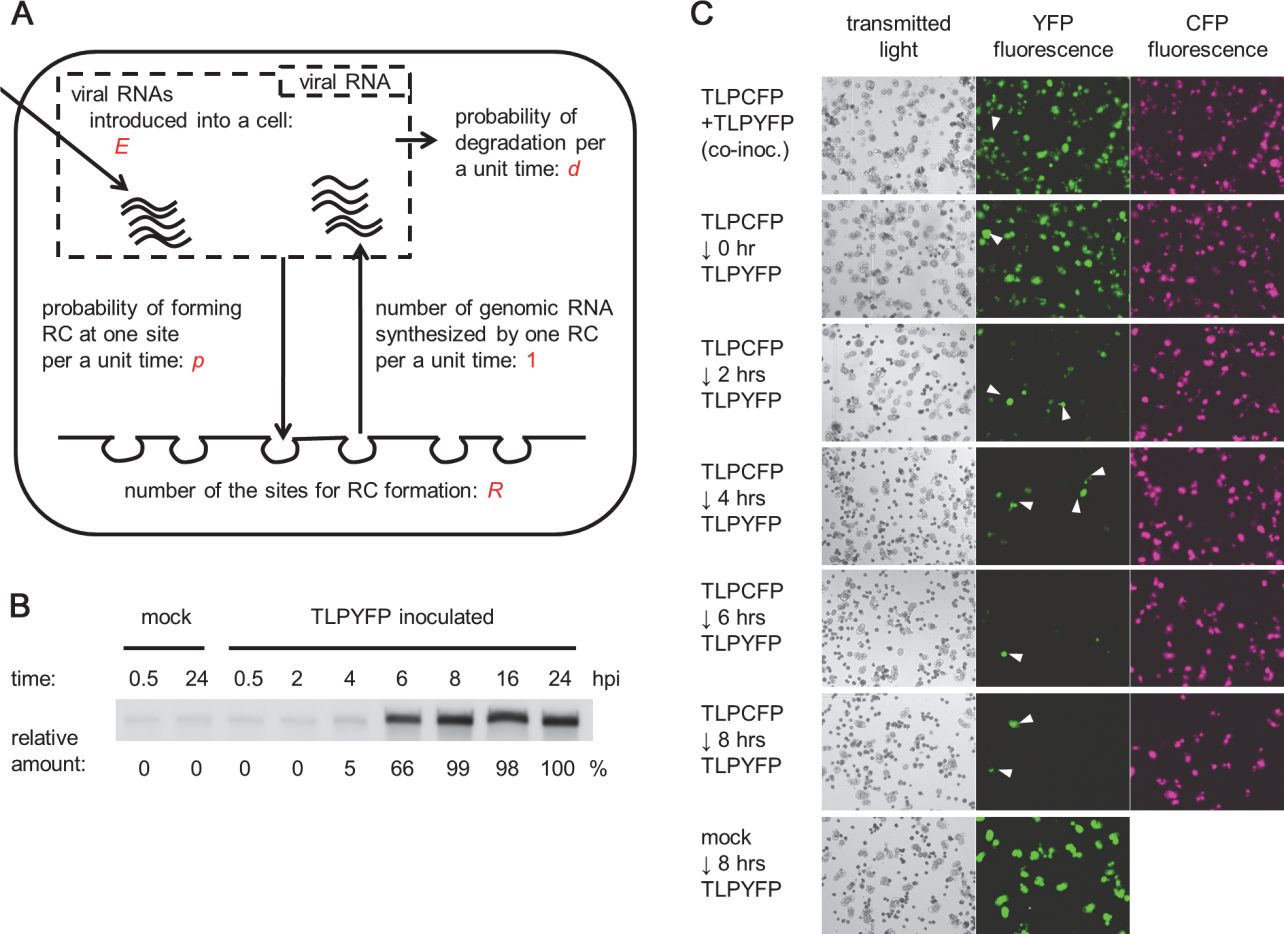
A model for single-cell infections. (A) A model for single-cell infections. (B) The accumulation of complementary-strand RNA at different time points. The results of northern blotting are shown, and the amount of complementary-strand RNA relative to that at 24 hpi is shown below the image. (C) Challenge inoculation with TLPYFP to TLPCFP-inoculated cells. Protoplasts were first inoculated with TLPCFP, followed by TLPYFP after the indicated intervals. Exceptionally, top panels show co-inoculation of TLPCFP and TLPCFP, and bottom panels show inoculation with TLPYFP 8 h after mock inoculation. Fluorescence microscopy images captured 40 h after initial inoculation (TLPCFP or mock inoculation) are shown. White arrowheads indicate cells that are singly infected with TLPYFP. The inoculation of TLPYFP 8 h after inoculation with TLPCFP did not result in coinfection of the variants, suggesting that TLPYFP did not infect the cells. The high infectivity of TLPYFP in the bottom panel rules out the possibility that protoplasts became incompetent by 8 h.

To clarify how the founder number is determined, we developed a simple stochastic model of ToMV single-cell infections (Fig. 2A), based on our current knowledge of the replication mechanism of ToMV and other positive-strand RNA viruses (reviewed in [13]). We first defined the number of genomic RNA molecules that were introduced into each cell as *E*. After introduction into the host cell, the genomic RNA molecules are translated by ribosomes to produce replication proteins, which are required for replication of the viral genome. The replication proteins recognize the genomic RNA molecules, recruit them to the cytoplasmic surfaces of host intracellular membranes, and form replication complexes (RCs) [14–16]. We assumed that a limited number (defined as *R*) of RCs are formed in a cell. In other words, we assumed that a host cell has *R* sites for RC formation. In the RCs, complementary-strand RNAs are synthesized using the genomic RNAs as templates. Then, using the complementary-strand RNA as a template, genomic RNA molecules are synthesized and released into the cytoplasm. We defined the time required for one RC to synthesize one genomic RNA molecule as one time unit. Complementary-strand RNAs are sequestered within the RCs and protected from cytoplasmic nucleases [14,15]; therefore, we hypothesized that RCs (and complementary-strand RNAs) are very stable. Unlike complementary-strand RNA, introduced genomic RNA molecules or newly synthesized progeny genomic RNA molecules released into the cytoplasm are degraded by nucleases in a stochastic manner. We defined the probability of a genomic RNA molecule being degraded in one time unit as *d*. We also defined the probability of a genomic RNA molecule forming RC at one RC formation site in one time unit as *p*. To simplify the case, the time periods required for RC formation were ignored.

Next, we estimated some of the parameter values. To estimate *E*, we inoculated protoplasts with radioisotope-labeled viral genomic RNA transcripts and found that 5 × 10^3^ ± 2 × 10^3^ viral genomic RNA molecules were introduced into a cell under the conditions used for estimating the founder number in Fig. 1C (mean ± standard deviation [SD] of three replicates). Thus, we assumed that *E* = 5 × 10^3^. This value might be overestimated because a fraction of the RNA molecules might not be functional (e.g., inappropriately synthesized or not capped) or might be delivered into cellular compartments other than the cytoplasm. However, as described below, we found that our model is robust to changes in parameter value for *E* as long as it is large enough: *E* can be 5 × 10^1^ or 5 × 10^2^ instead of 5 × 10^3^ without largely affecting the simulation results (see final section in the Results). To estimate *R*, we first quantified the accumulation of complementary-strand RNA. We found that complementary-strand RNA accumulates to ∼3 × 10^4^ molecules/cell 24 h after the inoculation of TLPYFP to protoplasts. This result is similar to a previous report for Flock House virus (FHV), which showed the accumulation of ∼2 × 10^4^ and ∼5 × 10^4^ complementary-strand RNAs for RNA1 and RNA2, respectively [17]. The same study reported the formation of 2 × 10^4^ RCs in a host cell using electron microscopy, suggesting that a mean of ∼1 and ∼2 complementary-strand RNAs are sequestered in an RC for RNA1 and RNA2, respectively [17]. To simplify the model, we assumed the presence of one complementary-strand RNA in one RC; therefore, *R* = 3 × 10^4^. Importantly, the assumed number of RC formation sites is much larger than the founder number, which suggests that after the first round of RC formations by the founders, multiple rounds of RC formations by the progenies occur until all the sites for RC formation become occupied. In a very early time of infection when most of the sites for RC formation are available, the probability that each genomic RNA introduced into a cell would form an RC can be approximated as *p* × *R*/*d*. Assuming that first-round RC formations by founders occur during this period of infection, the mean founder number (*λ* ≈ 4) can be approximated as *E* × *p* × *R*/*d*. Thus,

λ=E×p×R/d≈4,

where E = 5 × 10^3^, R = 3 × 10^4^; therefore,

$$p/d\approx 3\times 10^{-8}.$$ (1)

We performed several experiments to elucidate the kinetics of RC accumulation in inoculated protoplast cells. Time-course analysis using northern blot hybridization showed that in TLPYFP-inoculated protoplasts, complementary-strand RNAs were detectable at 4 hpi and accumulate until 8 hpi; the accumulation then reaches a plateau (Fig. 2B). This is consistent with our previous observations [18]. The amount of complementary-strand RNA did not decrease after stopping the formation of new RCs by adding the translation inhibitor cycloheximide to the protoplast media at different time points (S2 Fig., panel A; note that RC formation involves a translation-coupled step [12]). These results suggest that most RC formation sites were occupied before 8 hpi and that RCs were very stable once they were formed. Consistent with this finding, a challenge inoculation of TLPYFP into TLPCFP-inoculated cells showed that coinfection with TLPYFP and TLPCFP rarely occurred when TLPYFP was inoculated 8 h after the initial inoculation of TLPCFP (Fig. 2C). CFP fluorescence was barely detectable 8 h after the single inoculation of TLPCFP (S2 Fig., panel B), suggesting that plenty of nucleosides and amino acids were present in each cell at 8 hpi and that the failure of challenge infection was not due to their shortage. Therefore, we did not consider the degradation of RCs or the availability of substrates for translation or replication in the model.

### Simulation of the Course of Single-Cell Infections Using the Model

**Fig 3:**
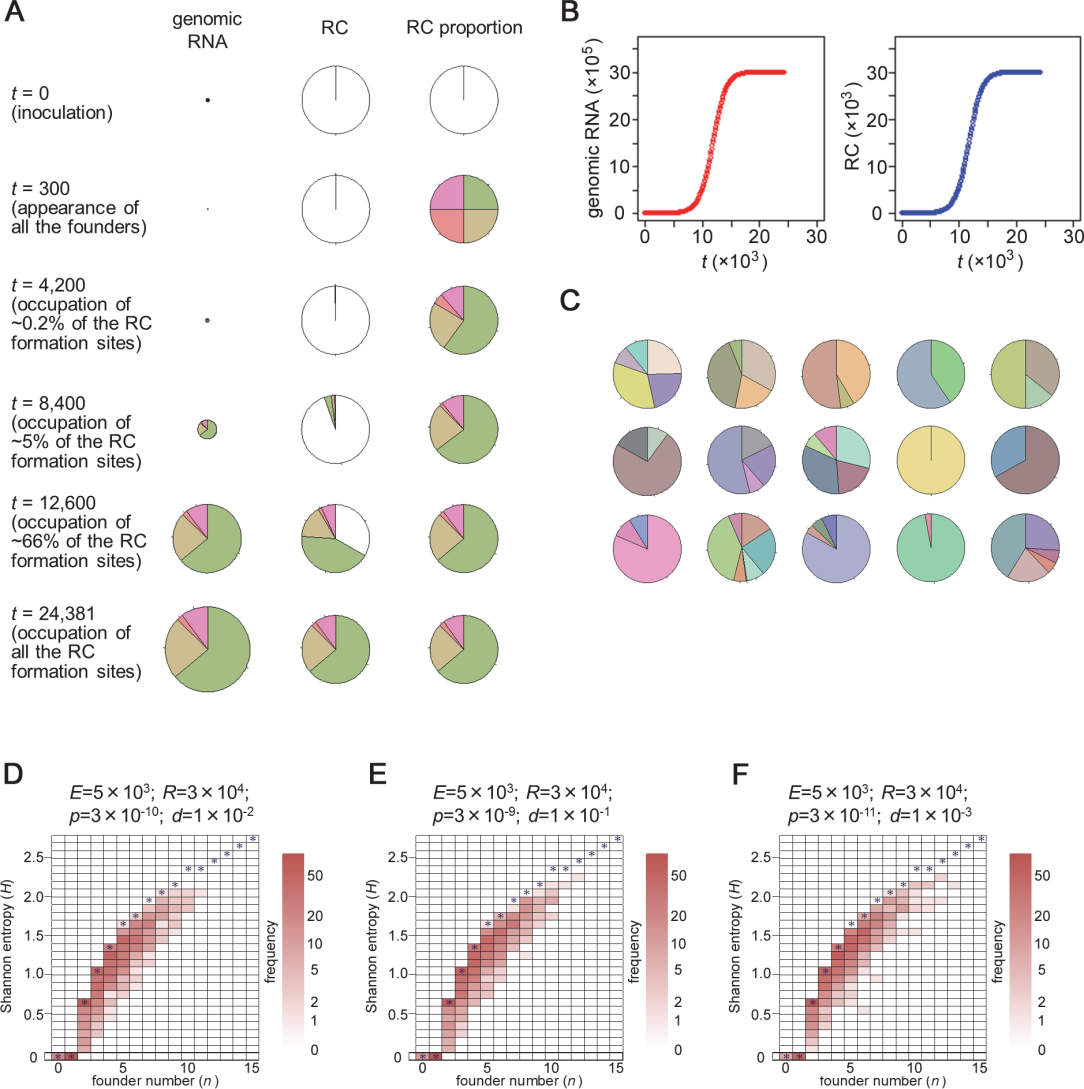
Simulation results for single-cell infections. (A) An example of simulated changes over time in the accumulation of genomic RNA and RC site occupancy in single-cell infections. Simulations were performed using the following parameters: *E* = 5 × 10^3^, *R* = 3 × 10^4^, *p* = 3 × 10^–10^, and *d* = 1 × 10^–2^. *t* indicates the time units that passed from the start of the simulation. Pie charts show the number of genomic RNAs, number of RCs, and proportion of RCs that originated from each founder. Note that the pie charts for number of RCs (middle) include empty sites for RC formation (indicated in white), whereas those for RC proportion (right) do not. For the number of genomic RNAs, the total amount in each time point is shown by an area of the pie chart. (B) The accumulation of total genomic RNA (left) and RCs (right) of the simulation shown in (A). (C) Examples of the simulated accumulation of progenies in 15 cells. The color codes were assigned automatically according to the identification number (from 1 to 5,000) of the founders. (D–F) Simulated founder numbers and progeny accumulations in 1,000-cell infections. The results are summarized in two-dimensional histograms, with founder number on the *x*-axis and Shannon entropy on the *y*-axis. The theoretically maximum Shannon entropy (i.e., the entropy in the case of equal accumulation) for each founder number belongs to each bin indicated by an asterisk. The parameter values used for the simulations are shown above each histogram. An R script used to generate panels A, B, and C is shown in S1 Text. To generate panels D, E, and F, an R script shown in S3 Text was used, and the data obtained by the authors are shown in S2 Data.

Using the above model, we simulated the behavior of each of the initially introduced 5 × 10^3^ genomes and their progenies using different combinations of the parameter values *p* and *d* under the constant ratio shown in Equation 1. Simulations were performed using R software [19], and the script is shown in S1 Text. An example result obtained using parameter values *p* = 3 × 10^–10^ and *d* = 1 × 10^−2^ is shown in Fig. 3A and 3B and S1 Movie. In the period shortly after inoculation (*t* = 0–300; *t* indicates time units passed after simulation started), the introduced RNA molecules were degraded stochastically, but a limited number of RNAs (the founders) form the first-round RCs before degradation to start the synthesis of progeny RNA (step 1). Then, RC formation by the progenies starts, and a stochastic variety in the number of RCs among the progenies arises (step 2). The proportion of RCs among the progenies becomes roughly fixed (step 3) before *t* = 4,200, when ∼0.2% of the RC formation sites (i.e., ∼60 sites) are occupied. The proportion then becomes almost fixed (step 4) before *t* = 8,400, when ∼5% of the RC formation sites (i.e., ∼1,500 sites) are occupied. Stochastic variety in the number of RCs among founders results in stochastic inequality in the progeny accumulation (SIPA) in each cell, which is further analyzed below. Finally, the sites for RC formation become occupied at ∼*t* = 24,000 (step 5). As a result, RCs belonging to different generations are formed in a cell, ranging from the first generation by the founders to about the 20th generation (S3 Fig.). The kinetics of RC accumulation in the simulation (Fig. 3B, right panel) resembles that of the accumulation of complementary-strand RNA in the experiments above (Fig. 2B), suggesting that *t* = 8,400 and *t* = 12,600 in the simulation (i.e., the time points that 5% and 66% of RC formation sites, respectively, get occupied) can be respectively approximated as 4 hpi and 6 hpi in the experiments. Assuming that a time unit in the simulation corresponds to a constant time in actual experiments, 1 h in experiments corresponds to ∼2,100 time units in the simulation. This suggests that steps 1–3 (i.e., founder appearance to rough fixation of the proportion of their progenies) occur before 2 hpi, when we could not detect the accumulation of genomic or complementary-strand RNA experimentally (Fig. 2B). Steps 4 and 5 correspond to ∼4 and ∼12 hpi, respectively, whereas ∼99% of RC formation sites were suggested to get occupied at ∼8 hpi, *t* = 16,800. Importantly, steps 1–4 occur when >95% of the RC formation sites are empty. This simulation result supports our experimental results, in which that lack of RC formation sites was not a cause of the small founder number. The results also suggest that the lack of RC formation sites is not a cause of SIPA. Instead, SIPA and the small founder number are attributable to the stochastic formation of RCs by the introduced viral RNAs and their progenies, as well as the stochastic degradation of the RNAs. Changing the values for parameters *p* and *d* under the constant ratio shown in Equation 1 did not largely affect the overall results, except for the final accumulation level of progeny RNAs and the relative length of time between the simulation and experimental data (see S4 Fig. for examples).

Simulation of infections in 15 individual cells predicted stochastic variation in founder number (SVFN) among the infected cells, as well as SIPA, when parameter values of *p* = 3 × 10^–10^ and *d* = 1 × 10^–2^ were used (Fig. 3C). We further simulated the inoculation of 1,000 cells, and the results are summarized as a two-dimensional histogram with the founder number (*n*) on the *x*-axis and Shannon entropy (*H*), which represents the variation in accumulation levels, on the *y*-axis (Fig. 3D). Shannon entropy was calculated using the following equation:

$$H=-\sum_{k=1}^{n}r_{k}\log_{e}r_{k},$$

where *r*_*k*_ is the proportion of progenies that originate in the *k*th founder out of *n* founders in the cell. Note that equal accumulation gives a maximum *H* of each founder number, and variation in the accumulation levels lead to lower Shannon entropy. The histogram showed distribution along the *x*-axis and along the *y*-axis of each founder number (≥2), suggesting that SVFN and SIPA occur, as well as that the degrees of SIPA vary among cells. When the parameter values for *p* and *d* were changed, keeping their ratio constant following Equation 1, the distributions in the two-dimensional histograms were not affected significantly (Fig. 3E, 3F). This suggests that the results of the simulation (i.e., the occurrence of SVFN and SIPA) are robust to changes in the values of these parameters.

### Experimental Demonstration of Occurrence of SVFN and SIPA

**Fig 4:**
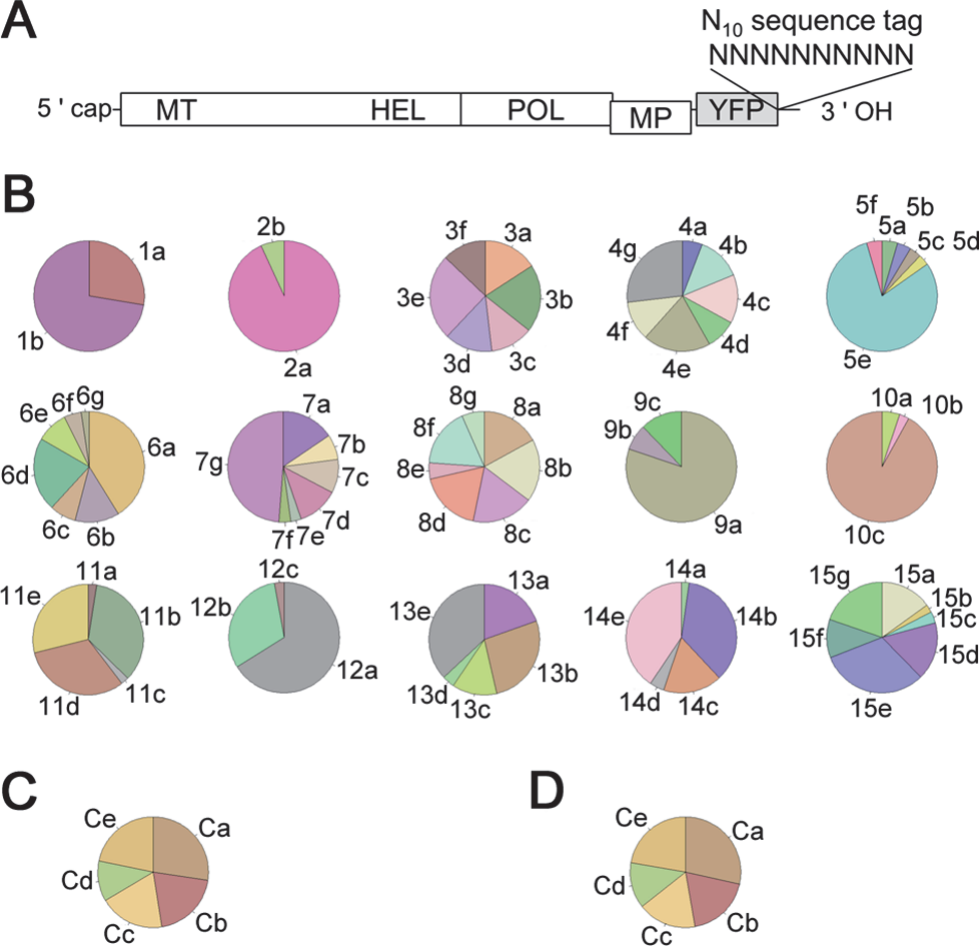
Stochastic variation in the numbers of founders and stochastic inequality in progeny accumulation in vivo. (A) A ToMV variant library tagged with random 10-nucleotide sequences. (B) The frequencies of tag sequences identified from each cell sample. Each pie chart corresponds to a cell sample. A tag ID code (e.g., “1a” and “1b”) was assigned to each tag sequence. The tag sequences with the tag ID codes and their detection frequencies are shown in S1 Table. The color codes were assigned automatically according to the identified tag sequences. (C,D) Control experiments. In control 1, a mixture containing equal amounts of five differently tagged viral RNAs was used as a template for RT-PCR (C). In control 2, RNA extracted from 1 × 10^5^ protoplasts inoculated with a mixture containing equal amounts of the five RNAs and cultured for 24 h was used as the template (D).

To investigate whether SVFN and SIPA are observed experimentally, we prepared a library of ToMV derivatives with random 10-nucleotide sequence tags (Fig. 4A). The library RNA was introduced into tobacco protoplasts by electroporation under the same conditions used for the above experiments. Because the library scale was 2.5 × 10^5^, 97.5 ± 0.3% of 5,000 viral RNAs introduced into each cell were expected to carry independent sequence tags according to our simple simulation (mean ± SD of 100-cell inoculations simulated). Considering that only a limited number of viral RNAs of the introduced 5 × 10^3^ would become founders, we ignored the possibility that the founders might coincidentally carry the same sequence tags. At 24 hpi, infected (YFP fluorescence-positive) cells were harvested individually using a glass capillary tube connected to a micromanipulator, and the tag-containing regions of the viral RNAs that had accumulated in each cell were amplified using reverse transcription–polymerase chain reaction (RT-PCR) and nested PCR. Then, 4 × 10^4^ to 4 × 10^5^ tag sequences per cell sample were analyzed using a Genome Analyzer IIx (Illumina, San Diego, California, United States). The numbers of obtained tag sequences (4 × 10^4^ to 4 × 10^5^) are not enough to know the identity of all the viral genomes in each cell, which accumulate to ∼10^6^, but are enough to know the approximate proportion of the progenies originating from each founder (see S4 Text for detailed assessments of the sampling error).

Among the 15 infected cells tested, the numbers of the tag sequences identified varied from 2 to 7, suggesting that 2–7 founders initiated the replication in each cell (Fig. 4B and S1 Table). Therefore, SVFN was observed in the cell infections. The mean founder number in these 15 cells was 5.0, which is comparable to the mean founder number of ∼4 that was estimated by the co-inoculation of two fluorescent-protein-tagged derivatives (Fig. 1C). The range of founder numbers (2–7) is comparable with a prediction assuming the Poisson process, supporting the stochastic nature of founder initiation. Specifically, with a mean founder number of 5.0, ∼83% of the infected cells are expected to have 2–7 founders (S5 Fig., panel A). The detection frequency of the tags in each infected cell varied greatly (Fig. 4B). To assess the effect of experimental artifacts such as bias in sequence tag detection and the difference in accumulation abilities among tagged viruses, we prepared transcripts of five ToMV variants carrying different sequence tags. The RT-PCR products of an equal mixture of these transcripts (control sample 1) and of total RNA extracted from 1 × 10^5^ cells at 24 hpi with the transcript mixture (control sample 2) were analyzed in the same way as single-cell samples. The control samples showed less-varied detection frequencies (Fig. 4C, 4D). Quantification of the variation showed that, in 14 of the 15 cells tested, the variation in the detection frequency was much larger than that observed in control experiments (See S5 Text and S5 Fig., panels B and C, for the quantification procedures and results). This suggests that SIPA occurs during actual infections. Moreover, the consistency between the experimental results and the simulation results suggests that the model describes the course of single-cell infections accurately.

### SVFN and SIPA Accelerate the Selection of Viral *Trans*-Acting Genes or Elements during Tissue Infection

**Fig 5:**
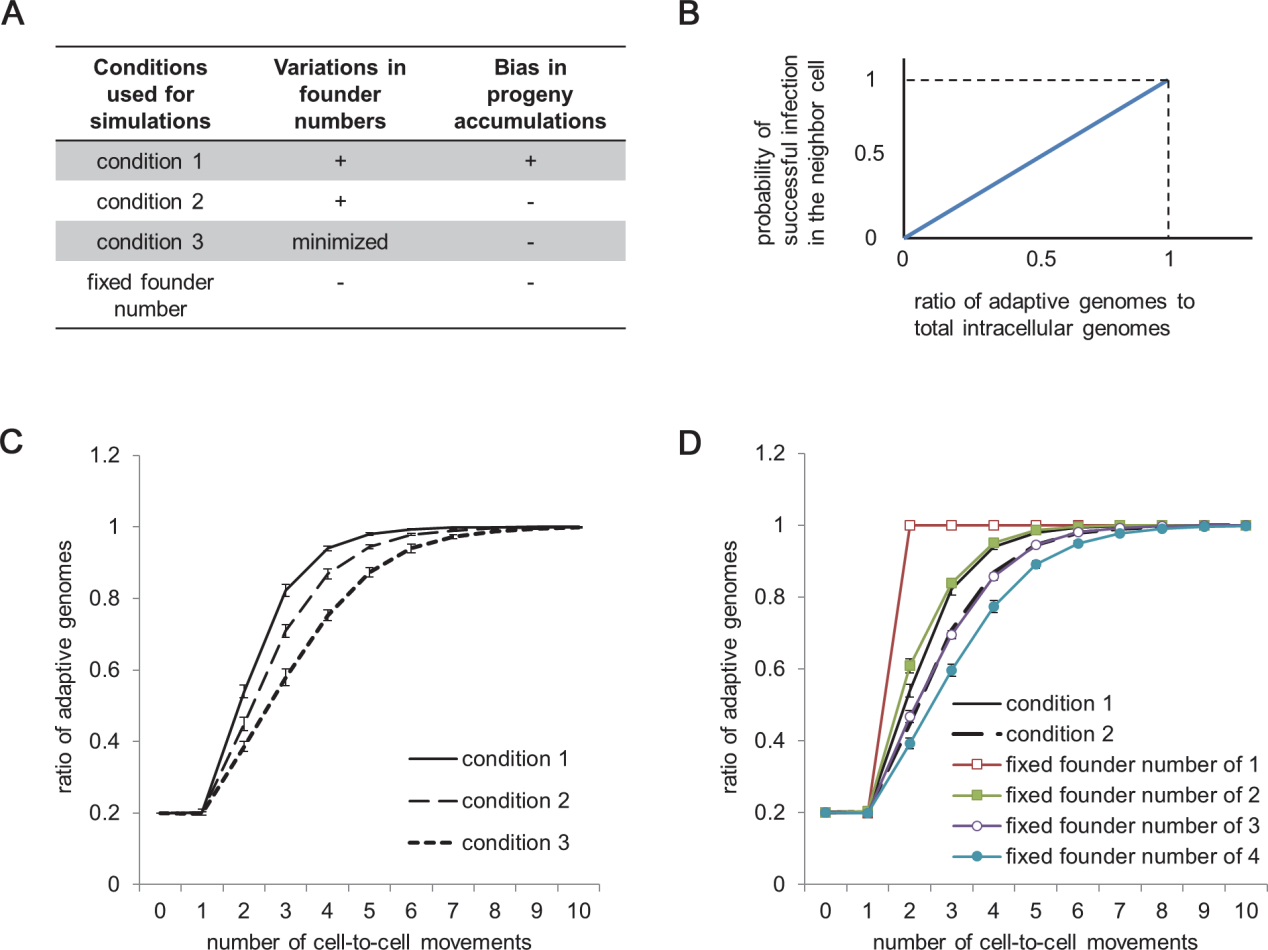
Simulation of the selection of *trans*-acting genes or elements at the tissue infection level. (A) A summary of the conditions used for the simulations. (B) Assumption of the probability of successful infection at a given ratio of adaptive genomes to the total number of intracellular genomes. (C) Comparison of the rate of adaptation under conditions 1–3. (D) Comparison of the rate of adaptation under conditions 1 and 2 with conditions assuming a fixed founder number and equal progeny accumulation. Data in panels C and D are presented as means ± SE, which were calculated by bootstrap analysis of 1,000 simulated cells. An R script used to generate panels C and D is shown in S6 Text, and the data obtained by the authors are shown in S3 Data.

As described above, infection with a small number of genomes enhances the selection on *trans*-acting genes or elements by isolating adaptive and defective genomes stochastically to counter the negative effects of complementation during tissue infection (S1 Fig.). If SVFN and SIPA also occur during tissue infections, they might further enhance selection by stimulating genome isolation. To assess whether enhanced selection occurs, we developed an additional simulation model and used it to compare three conditions (summarized in Fig. 5A). Condition 1 assumes the occurrence of both SVFN and SIPA; we consider that condition 1 reflects actual infections. Condition 2 assumes the occurrence of SVFN, but not SIPA (i.e., equal accumulation of progenies of each founder). Finally, condition 3 assumes minimized variation in the founder number and no SIPA. To include the effects of SVFN and SIPA under condition 1, we used the results of the simulation for single-cell infections using the following parameter values: *E* = 5 × 10^3^, *R* = 3 × 10^4^, *p* = 3 × 10^–10^, and *d* = 1 × 10^–2^. From the pool of simulation results for 1,000 cells (Fig. 3D), randomly selected simulation results (i.e., the founder number and progeny accumulation in a specific cell) were assigned to each cell infection. Similarly under condition 2, randomly selected results from the same pool of simulations (i.e., founder number) were assigned to each cell infection. Under condition 3, the founder number was set randomly at 4 or 5, with the probability that the mean founder number is equal to conditions 1 and 2 (i.e., 4.34).

The structure of the model is as follows: we assumed an initial 1,000 intracellular populations, each of which consists of 20% adaptive genomes and 80% defective genomes. For simplicity, the defective genomes were assumed to be totally unable to continue infections without complementation by the adaptive genomes. During tissue infection, the ratio of adaptive to defective genomes diversifies among each lineage. The probability of successful infection in the next cell by an intracellular population consisting of exclusively defective genomes is zero, whereas that by an intracellular population consisting of exclusively adaptive genomes should be high. Conversely, an intracellular population with an intermediate proportion of adaptive genomes will infect the next cell at an intermediate probability. To model this situation, we assumed a linear relationship between the proportion of adaptive genomes in an intracellular population and the probability of successful infection in the next cell, with a maximum probability of 1 in case of the population consisting of exclusively adaptive genomes (Fig. 5B). For each successful infection, the founder number and the accumulation ratio of the progenies were determined according to each of the three conditions. The founder genomes were selected randomly, regardless of whether the genome carried adaptive or defective genes. The decisions regarding successful or abortive infections, founder number, and the accumulation ratio of the progenies were repeated for ten successive infection cycles. For each cell-to-cell infection cycle, cells that were not infected because of abortive infections were filled with intracellular populations selected randomly from the other intracellular populations that had successfully infected the cells at the infection cycle.

The simulations showed that the ratio of adaptive genomes in the 1,000-cell population increased from 20% to 90% after four successive infection cycles under condition 1, whereas five and six infection cycles were required for conditions 2 and 3, respectively (Fig. 5C). The difference in the number of required infection cycles is not small, because the continuous production of defective mutants in each cell infection cycle is not considered in this simulation. Because viruses need to remove the defective mutants from the population continuously during actual infections, differences in the selection speed are critical for viruses to maintain their genomes. Comparing condition 1 with simulations assuming fixed founder numbers of 1, 2, 3, and 4 with no SIPA (Fig. 5D), the rate of adaptation in condition 1 was comparable with that of the fixed founder number of 2, although the mean founder number for condition 1 was 4.34. Therefore, SVFN and SIPA strongly enhance selection on *trans*-acting genes or elements while also securing successful infection after cell-to-cell transmission by keeping the founder number large. A population genetic consideration revealed that the rate of adaptive evolution was determined by the effective population size that incorporated the effects of SVFN and SIPA (See S7 Text).

### Multiple-Round RC Formation Results in Accelerated Selection of *Cis*-Acting Genes or Elements at the Single-Cell Level

**Fig 6:**
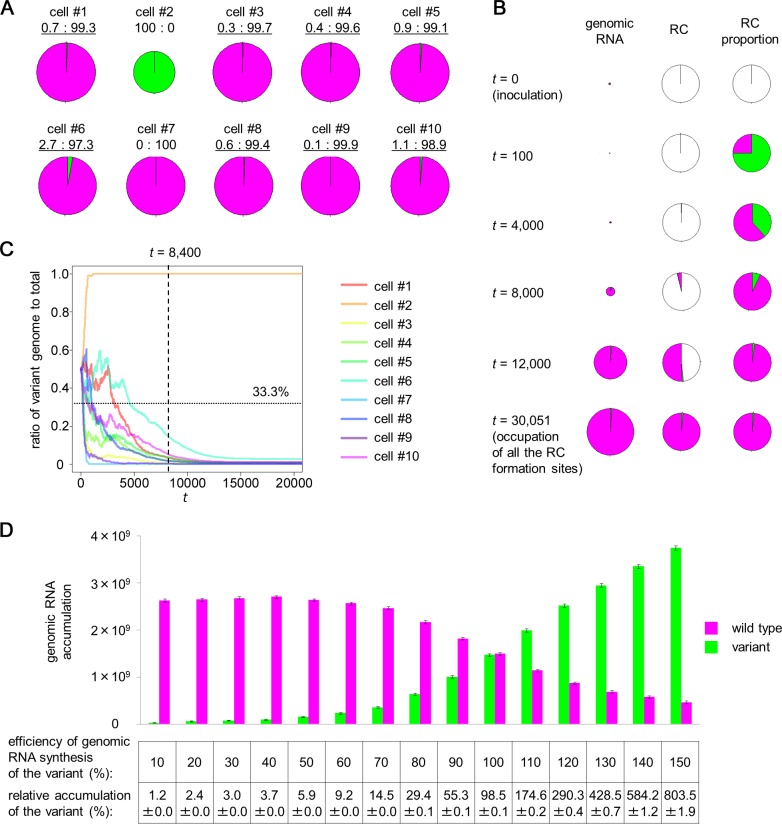
Enhanced bias in progeny accumulation between variants in *cis*-acting genes or elements simulated by the model. (A) Simulation of the accumulation of the wild type (WT; magenta) and a variant with 50% efficiency of genomic RNA synthesis (green), which were co-inoculated into cells at a 1:1 ratio. Each pie chart shows the amount of genomic RNAs that accumulated in each cell. The accumulation ratios of the variant to WT are indicated below the pie charts, and underlining indicates coinfections. The area size of the pie charts represents the total amount of genomic RNAs. (B) An example of a simulated change in genomic RNA accumulation and replication complex (RC) site occupancy over time in a cell coinfected with the WT (magenta) and a variant with 50% genomic RNA synthesis efficiency (green). (C) History of changes in the ratio of the variant to total genomes in cells 1–10 of panel A. The dashed line indicates *t* = 8,400, which corresponds to experimental ∼4 hpi. The efficiency of genome RNA synthesis of the variant is 50% of that for the co-inoculated WT RNA; therefore, a variant to total ratio of <33.3% [i.e., 50%/(50% + 100%)] indicates the occurrence of enhanced bias in progeny accumulation (EBPA). The dotted line indicates 33.3% accumulation of the variant. (D) The accumulations of WT (magenta) and co-inoculated variants with different genomic RNA synthesis efficiencies (green) in 1,000 cells were simulated. The relative accumulation of the variants is also shown in a table below the bar graph. Means ± SEs calculated using bootstrap analysis of 1,000 simulated cellular infections are shown. R scripts S9 Text and S10 Text were used to generate panels A–B and C, respectively. S9 Text was also used to generate S2 Movie. To generate panel D, R scripts S11 Text and S12 Text were used, and the data obtained by the authors are shown in S4 Data.

As described in the Introduction, *cis*-acting genes or elements are more easily selected than *trans*-acting genes or elements because differences in the fitness of each variant will be reflected directly on its accumulation. Importantly, multiple-round RC formations during single-cell infections might amplify the difference in the efficiency of single-round progeny production of the variants, resulting in enhanced bias in progeny accumulations (EBPA) between the coinfected variants that carry variations in *cis*-acting genes or elements. In the context of evolution, EBPA can be considered an enhancement of selection on *cis*-acting genes or elements. Therefore, we assessed whether EBPA could occur in simulations using the single-cell infection model. We first simulated the inoculation of 1,000 cells with a 1:1 mixture of wild-type (WT) and variant viruses, assuming that the genomic RNA synthesis efficiency of the variant is 50% of the WT virus. We used the following parameter values: *E* = 5 × 10^3^, *R* = 3 × 10^4^, *p* = 3 × 10^–10^, and *d* = 1 × 10^–2^ (An R script used for the simulation can be found as S8 Text). The simulations expected coinfection in 820 of 1,000 cells, the exclusive infection with the WT or the variant in 171 cells (88 by the WT and 83 by the variant), and no infection (because of SVFN) in nine cells (Fig. 6A shows ten examples of infected cells). In the coinfected cells, the relative accumulation of the variant was only 0.002%–19% of that of the WT (median, 0.5%), predicting the occurrence of EBPA. A change occurred in the competition in coinfected cells over time (Fig. 6B and S2 Movie): the variant could form the first-round RCs at a rate that was equivalent to the WT’s, but the defect in progeny synthesis resulted in lower efficiency in the second and subsequent rounds of RC formation. Therefore, the relative occupancy of RC formation sites by the variant gradually but rapidly decreased. The effect of EBPA became obvious before *t* = 8,400, the time point corresponding to ∼4 hpi experimentally (Fig. 6C).

**Fig 7:**
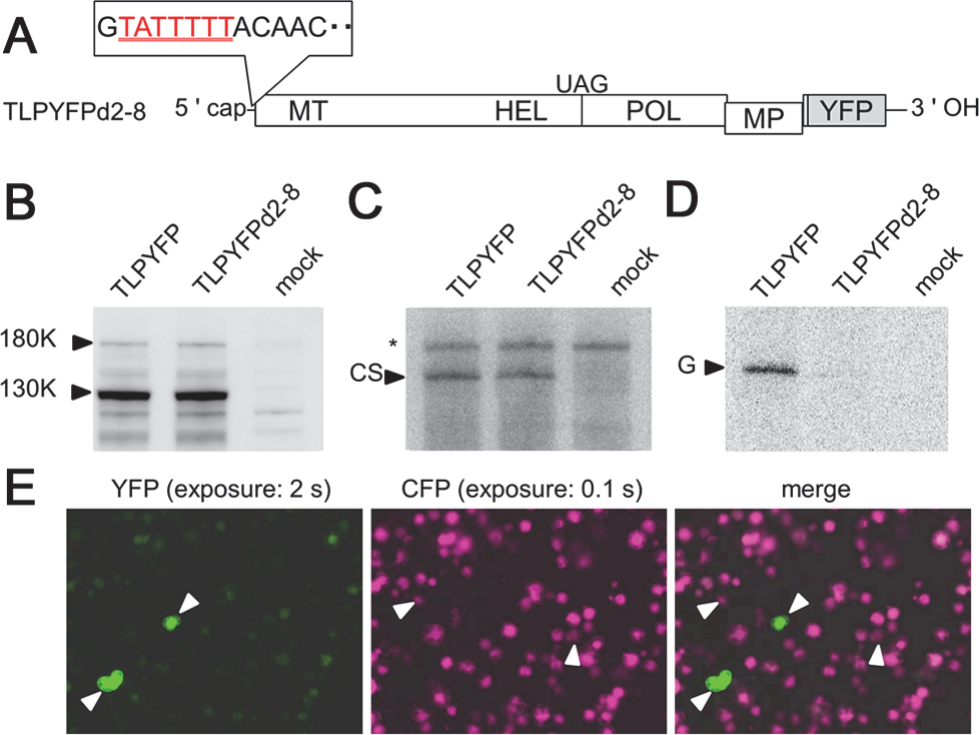
Enhanced variation in progeny accumulation demonstrated experimentally. (A) Structure of TLPYFPd2–8. The deleted region (nt 2–8) is shown by underlined red letters. (B–D) Characterization of TLPYFPd2–8 using an in vitro translation–replication system. (B) Western blotting of the translation products of TLPYFPd2–8 and parental TLPYFP without the 5′-UTR mutation. Arrowheads indicate the positions of the two replication proteins synthesized by translation: 130K and its stop codon read-through product 180K. (C) RNase protection assay (RPA) to detect complementary-strand RNA. An arrowhead indicates the position of the protected RNA originating from complementary-strand RNA (CS). An asterisk indicates the position of an unintended RNA band that originates from the minor complementary transcripts generated during probe preparation. (D) RPA of genomic RNA. An arrowhead indicates the positions of the protected RNAs originating from the genomic RNA (G). (E) Co-inoculation of TLPYFPd2–8 and TLPCFP. The images were obtained at 40 hpi and processed in the same way as described in Fig. 1. Note that the exposure period differs for the detection of YFP and CFP. Arrowheads indicate singly infected cells.

To determine whether EBPA is observed experimentally, we prepared the ToMV variant TLPYFPd2–8 (Fig. 7A) by introducing a 7-nucleotide deletion into the 5′-untranslated region (UTR) of TLPYFP from nucleotides 2 to 8 [20]. In an in vitro ToMV RNA translation and replication system [21,22], which allows the translation of viral genomes and single-round RC formation and subsequent genomic RNA synthesis, TLPYFPd2–8 showed a comparable ability to be translated and to synthesize complementary-strand RNA as parental TLPYFP; however, it showed a reduced ability to synthesize genomic RNA (Fig. 7B–7D). Inoculation of a 1:1 mixture of TLPYFPd2–8 and TLPCFP into protoplasts showed that a small number of cells were infected by either of the two variants, whereas others were coinfected. Among the coinfected cells, the fluorescence intensity of YFP was uniformly low compared to the cells infected with TLPYFP d2–8 alone (Fig. 7E). In contrast, the fluorescence intensity of CFP in coinfected cells was comparable to that in cells infected with only TLPCFP. These results suggest that the accumulation of TLPYFPd2–8 was suppressed strongly when coinfected with TLPCFP, confirming the occurrence of EBPA.

We further simulated the co-inoculation of 1,000 cells with a 1:1 mixture of WT and variant viruses with genomic RNA synthesis efficiencies ranging from 10% to 150% (Fig. 6D). Intriguingly, even the variants with 90% and 80% genomic RNA synthesis efficiency were predicted to accumulate to only 55.3 ± 0.1% and 29.4 ± 0.1%, respectively, of the WT virus in 1,000 cells, whereas the variants with 110% and 120% efficiency were predicted to accumulate to 174.6 ± 0.2% and 290.3 ± 0.4%, respectively, of the WT virus (mean ± SE, calculated by bootstrap analysis). These results suggest that even a small difference in fitness is amplified to cause a large bias in progeny accumulation. Similar results were obtained when RC formation efficiency was varied instead of genomic RNA synthesis efficiency (S6 Fig.). Therefore, enhanced selection might occur on all *cis*-acting genes or elements that are involved in replication, irrespective of the step at which the variation affects replication efficiency or the extent and direction of the variation.

### The Model and Deduced Viral Adaptation Mechanisms Are Robust to Changes in the Parameter Values

We analyzed the parameter values used in the single-cell infection model to identify the permissible ranges that support the occurrence of infection by a small founder number, SVFN, SIPA, and EBPA, which were all confirmed to occur experimentally and suggested to be important for viral adaptation. First, *E* × *p* × *R*/*d*, which mainly determines the mean founder number *λ*, should be low to achieve a small founder number. We fixed *E* × *p* × *R*/*d* to 4.5. Next, the parameter values of *E* were assessed under a fixed *R* of 3 × 10^4^ (S7 Fig., S8 Fig., and S9 Fig.). Changing *E* from 5 × 10^3^ to 5 × 10^4^ or 5 × 10^5^ did not affect the occurrence of SVFN, SIPA, or EBPA. Similarly, *E* = 5 × 10^2^ or 5 × 10^1^ could reproduce these phenomena by applying the appropriate *p* and *d* values, whereas *E* = 5 × 10^0^ could not reproduce SVFN and had a limited effect on EBPA because of the decreased frequency of coinfection. Therefore, *E* should be larger than 5 × 10^0^.

Next, the parameter values for *R* were assessed for the same criteria using a fixed *E* of 5 × 10^3^ (S10 Fig., S11 Fig., and S12 Fig.). Data revealed that *R* = 3 × 10^1^ showed limited SIPA and EBPA, and *R* = 3 ×10^2^ showed limited EBPA. In contrast, the criteria were fulfilled by *R* ≥ 3 × 10^3^ when the appropriate *p* and *d* values were applied. Therefore, *R* should be larger than 3 × 10^2^. This result can be explained by the number of RC formation cycles. A larger *R* leads to more cycles for RC formation, resulting in SIPA and EBPA. Finally, the presence of lower limits for *E* and *R* indicates the existence of an upper limit for the *p*/*d* ratio because *E* × *p* × *R*/*d* is fixed. *E* > 5 × 10^0^ at *R* = 3 × 10^4^ corresponds to *p*/*d* < 3 × 10^–5^, whereas *R* > 3 × 10^2^ at *E* = 5 × 10^3^ corresponds to *p*/*d* < 3 × 10^–6^. Therefore, *p* cannot be too large, and *d* cannot be too small, even though their permissive ranges depend on each other. Taken together, the permissive ranges for these parameters to support rapid adaptation are likely rather broad, suggesting that the simulation model and the adaptation mechanisms are robust to changes in these parameters.

## Discussion

### Mechanisms That Determine the ToMV Founder Number

We estimated that the mean ToMV founder number after cell-to-cell transmission was ∼4, thereby providing an additional example of the establishment of cell infections by a small number of genomes after viral cell-to-cell transmission. Possible explanations for the mechanism behind the occurrence of the small founder number were that only four genomic RNA molecules were transmitted to the adjacent cells or that only four RCs were formed in an infected cell. However, these possibilities were denied in the case of inoculations of ToMV RNA transcripts to protoplasts. Specifically, the protoplast inoculation experiments showed that cell infections by ∼4 founders occur when thousands of viral RNA molecules are introduced into each cell and that >10,000 RCs can be formed in each cell. In addition, a simulation model for single-cell infections showed that SVFN, SIPA, and EBPA, which were observed experimentally, could not be reproduced when assuming the transmission of a small number of genomes or the formation of a small number of RCs. Protoplast inoculation experiments also ruled out the possibility that availability of the substrates required for viral gene expression or replication limits the founder number. Instead, the experiments and simulations suggested that founder number is determined by the stochastic behavior of the viral genomes. The probability that the viral genomes will get degraded is much higher than the likelihood that they will form RCs and initiate replication, resulting in low numbers of founders. However, the proposed mechanism might not be applicable to all viruses because the degradation rate of the genome might differ dramatically between RNA viruses and DNA viruses and because viruses employ a diverse range of replication mechanisms. Future studies might reveal the varying mechanisms used to control founder number by different viruses.

### The ToMV Infection Process and Adaptation

Based on all of the experimental and simulation results, we here provide a comprehensive view of the tissue infection process of ToMV, from the stochastic behavior of viral genomes to adaptation. First, many viral genome molecules enter a cell from an adjacent infected cell. Most of the introduced genome molecules are degraded, and only a small number of them stochastically form RCs. Therefore, the founder number in each cell is determined stochastically, theoretically following the Poisson distribution (i.e., the occurrence of SVFN). In each cell, the RCs formed by the founders synthesize their progenies, that is, second-generation genomes. The progenies also get degraded at a high probability and form RCs at a low probability. The RCs formed by the second-generation genomes synthesize third-generation genomes, which also get degraded or form RCs. Progeny synthesis by RCs and RC formation by the progenies are repeated for multiple rounds until all of the 30,000 RC formation sites are occupied ∼12 h after introduction into the cell. Thus, RCs formed in a cell belong to different generations, presumably from the first to about the 20th generation.

Stochastic and repeated RC formations bring stochastic inequality to the number of RCs that are formed by the progenies of each founder during the early period of cell infection (within 4 h of introduction). This stochastic inequality in the number of RCs causes SIPA. The importance of stochastic events in the early period of infection has also been suggested in a preceding simulation study on a negative-strand RNA virus: stochastic inequality in the amount of replication protein present in the early period of cell infection was suggested to cause large difference in viral RNA accumulation levels among infected cells [23]. After this early period of cell infection, the relative amounts of RCs and progeny genomes become almost fixed because the RCs and progenies have accumulated to amounts that are not affected significantly by the stochasticity of viral genome behavior. ToMV spreads from cell to cell every ∼4 h (S13 Fig.), suggesting that cell-to-cell transmission occurs after SIPA arises. Therefore, from the unequally accumulated progeny population in a cell, thousands of viral genomes may move to the next cell, and the above cell infection process is repeated. Importantly, viral RNAs and proteins are barely detectable by 4 h after introduction, but important events to determine the proportion of progenies occur in this early period. The simulation model for single-cell infections developed in this study helped us understand the early events of infection that we cannot experimentally analyze by current techniques.

In the present simulation models, the effect of viral genome encapsidation is not taken into account. Previous studies have suggested that encapsidation negatively affects viral replication by sequestering viral genomes, preventing them from serving as replication templates in the late period of cell infection [24,25]. Furthermore, a shortage of RNA degradation machinery and other physiological changes may also occur in the late period of cell infection. These events are presumed to affect the RC and RNA accumulation kinetics in the late period of cell infection but should not affect the occurrence of SVFN, SIPA or EBPA, which arise in the early period of cell infection.

From the viewpoint of viral adaptation, the above infection process is an excellent system. Small founder number, SVFN, and SIPA strongly enhance the stochastic isolation of adaptive and defective variants, which leads to rapid selection on *trans*-acting genes or elements at the tissue level. Recently, Schulte and Andino [26] reported extensive variation in poliovirus accumulation levels among infected cells, which likely originated from stochastic differences among cells. Although this effect was not taken into consideration when constructing our current model, it may further enhance SVFN and accelerate selection. The selection on *cis*-acting genes or elements is also accelerated at the single-cell level, via the amplification of difference in replication ability during multiple-round formation of RCs (i.e., EBPA). The effect of EBPA becomes obvious before 4 hpi, suggesting that EBPA could also play a role in adaptation during host tissue infections. In this study, we simulated the exclusion of defective variants that had already been present in the viral population. However, in an actual infection of host tissue, defective variants would arise in every cell infection cycle, resulting in a generation-exclusion equilibrium of defective genomes. A small founder number, SVFN, SIPA, and EBPA should suppress the proportion of defective genomes in intrahost populations. This will secure the efficiency of host-to-host transmission, which may be the most inefficient step in the viral life cycle.

This study also revealed that a small founder number, SVFN, SIPA, and EBPA could be reproduced by broad ranges of parameters using a single-cell model. This result primarily ensures the reliability of the model and the ToMV adaptation system described above. However, more importantly, this result suggests that the adaptation system is not unique to ToMV but is also employed by other positive-strand RNA viruses that use different host factors but replicate via similar mechanisms. ToMV and other positive-strand RNA viruses might commonly employ a set of parameters to achieve rapid adaptation as follows: a “not too small” number of introduced genomes (*E*, larger than the number of founders), a large number of RC (*R*, much larger than the number of founders), a “not too small” probability of degradation (*d*), a “not too large” probability of forming RC (*p*), and the appropriate *E* × *p* × *R*/*d* in total. This hypothesis argues against the idea that viruses with a higher probability of initiating infection (*p*) and a lower probability of getting degraded (*d*) are always advantageous. Positive-strand RNA viruses may have adopted and/or successfully adapted to host factors for cell-to-cell transmission, replication, and/or degradation that give rise to appropriate *E*, *R*, *p*, and *d* values.

### Other Virological Insights

In addition to the implications for viral adaptation, this study provides insights regarding other important virological issues. The first example is the mechanisms behind the high particle-to-plaque forming unit (PFU) ratio. Some viruses are known to have a very high particle-to-PFU ratio, which means that the number of initially infected cells is much smaller than the number of particles used for inoculation. For example, the particle-to-PFU ratio of poliovirus is estimated to be 30–1,000 [27]. A high particle-to-PFU ratio has been often explained by the introduction of a low frequency of intact particles and a high frequency of junk particles into cells and a low efficiency of particle introduction. However, our simulations and experiments added “stochasticity in cell infection” as another possible factor that leads to high particle-to-PFU ratio, whereby many particles (or genomes) having equivalent but low probabilities of initiating replication invade a cell.

Another example is the interpretation of the fitness of viral variants estimated in different types of experiments. Our previous study revealed that a ToMV variant, LT1^D1097Y^, which carries three amino acid substitutions in viral replication proteins, showed similar levels of genomic RNA accumulation as WT ToMV when inoculated separately into protoplasts but showed impaired accumulation (∼40% of the WT virus) when co-inoculated with the WT virus [28]. Because the ToMV replication proteins replicate the genomic RNA preferentially in *cis* [12,29], this can be clearly explained by the EBPA described in the current study. A comparison of the level of accumulation after co-inoculation (∼40%) with the simulation results shown in Fig. 6D and S6 Fig. suggests that the progeny accumulation efficiency of LT1^D1097Y^ in a single-round of RC formation and replication was ∼80%–90% of that of the WT virus. This suggests that we need to distinguish the fitness values estimated by considering the productivity of single inoculations (e.g., [11]) from those estimated by considering viral accumulation after coinfection (e.g., [30]), as well as from those estimated by single-round replication assays using an in vitro system (e.g., Fig. 7). Nevertheless, the values obtained from these types of estimations are all meaningful.

Our simulation model for cell infection was developed to examine infections via cell-to-cell transmission, in which the number of viral genomes introduced into a cell, *E*, is likely to be large. However, the model is also applicable to other infection scenarios, e.g., aerosol or physical contact transmission between an infected and uninfected host, in which *E* is small. In this model, interactions characterized by small *E* (without changing other parameters) lead to frequent occurrence of cells with “zero founders,” that is, cells that do not get infected (S2 Table). This result suggests that when a host cell becomes infected under a small-*E* condition, there are likely to be far more cells that had been challenged by virus particles but were not infected. Further investigation will be necessary to validate such a scenario, with a particular focus on the number of virus particles that challenge a cell, the number of cells challenged, and the viral infection competency of the target cells.

### Concluding Remarks

In this study, we developed a simple model to analyze the course of single-cell infection of a positive-strand RNA virus. This model explains the dynamics of cell infections, as well as the entire infection process including evolution. We believe that this work establishes an important basis for understanding the life of positive-strand RNA viruses. It also provides a description of life-associated “large” systems that integrate the stochastic behaviors of molecules.

## Materials and Methods

### cDNA Construction of the ToMV Variants

To construct pTLPYFP and pTLPCFP, the coat protein (CP) coding region of pTLW3 [31], an infectious cDNA plasmid for ToMV, was substituted with the YFP and CFP coding sequences connected to nuclear localization signal (NLS) sequences that were derived from pJS2.NLSYFPp19 and pJS2.NLSCFPp19 [5] (DDBJ AB499725 and AB499726, respectively). pTLPYFP-CP and pTLPCFP-CP were constructed by inserting the YFP and CFP genes, respectively, between the MP and CP genes of pTLW3. The cDNA construct for TLPYFPd2–8 was prepared by deleting the second to the eighth nucleotides in the 5′-UTR. To construct a cDNA library of ToMV variants tagged with a random 10 nucleotides, the CP coding region of pTLW3 was substituted for the YFP-coding sequence containing a *Bst*EII restriction site directly downstream of the termination codon. The resultant plasmid was cleaved at the *Bst*EII and downstream *Bss*HII sites and was filled with a library of fragments containing random 10-nucleotide sequence tags. The tags were generated by annealing two oligo DNA fragments (To14 and To15) and then synthesizing the complementary strands using the Klenow fragment (TaKaRa Bio, Ohtsu, Japan). In total, 2.5 × 10^5^ independent colonies were obtained after transforming *Escherichia coli* JM109 cells using electroporation, and the cDNA library was extracted from these colonies without further amplification. The positions and the nucleotide sequences of the oligo DNA fragments used in this study are shown in S14 Fig. and S3 Table.

### In Vitro Transcription and RNA Purification

The cDNA plasmids and cDNA library of the ToMV variants were linearized at the *Mlu*I restriction site and transcribed using an AmpliCap-Max T7 RNA polymerase kit (CellScript, Madison, Wisconsin, US). After transcription, the template DNA was digested using DNase I and then purified using an RNeasy RNA purification kit (Qiagen, Hilden, Germany). The purified transcripts were quantified using a Qubit fluorometer (Invitrogen, Carlsbad, California, US).

### Inoculation of Tobacco Protoplasts and Tobacco Plants

The preparation and inoculation of tobacco BY2 protoplasts were performed as described previously [32] using 6 μg of transcripts to inoculate 1 × 10^6^ cells by electroporation. After inoculation, the protoplasts were incubated in protoplast culture medium [33] at 25°C in the dark. For the challenge inoculation experiments, the primary inoculated protoplasts were incubated in protoplast media and then washed with electroporation buffer before the second (challenge) inoculation. For inoculation of the tobacco plants, the inoculum was prepared by inoculating protoplasts with a mixture of TLPYFP-CP and TLPCFP-CP, followed by homogenization at 24 hpi in 10 mM sodium phosphate buffer (pH 7.0). The tobacco plants were grown at 25°C for 7–8 weeks before inoculation, and the fifth or sixth true leaves were inoculated with the homogenate using carborundum as an abrasive. The inoculated leaves were rinsed with water, and the plants were kept in the dark at 25°C.

### Fluorescence Microscopy

YFP and CFP fluorescence were observed using a fluorescence microscope (Axiophot; Carl Zeiss, Oberkochen, Germany). Images were captured using a CCD camera Retiga EXi Aqua (QImaging, Surrey, British Columbia, Canada) and analyzed using Image-Pro Plus software (Media Cybernetics, Rockville, Maryland, US).

### Estimation of the Founder Numbers in ToMV Cell Infections

The founder number was estimated as described previously [5]. Briefly, we assumed that each cell was infected by *n* viral genomes that were selected stochastically from a mixture of the two differently labeled virus variants A and B at a ratio of *r*:(1 − *r*). The probability that all *n* genomes consist of one of the variants, A or B, is *r* ^*n*^ or (1 − *r*)^*n*^, respectively, whereas the probability that the *n* genomes consist of both A and B is 1 − *r* ^*n*^ − (1 − *r*)^*n*^. Knowing *r*:(1 − *r*) of the source of infection (for the initial cell infection on leaves and protoplasts, we used a ratio of 0.5:0.5; for infections after cell-to-cell transmission, we assumed a binomial distribution of the previous infection), we could compare the actual ratio of single infections to coinfections with the calculated ratio, *r* ^*n*^ + (1 − *r*)^*n*^:1 − *r* ^*n*^ − (1 − *r*)^*n*^, and could thus estimate *n*. For the actual estimation, we assumed the Poisson distribution for *n* with mean *λ*, where *λ* was estimated. The raw data used for the estimations were composed of the frequencies of the observed coinfected and singly infected sites (14 and 19, respectively) and the frequencies of the observed coinfected and singly infected cells (234 and 77, respectively) in 45 coinfected sites with 5–9 infected cells. These data were then used to estimate the mean founder number for the first cell-to-cell transmission. In addition, the frequencies of the observed co- and singly infected cells (275 and 89, respectively) were used to estimate the mean founder number in the protoplast inoculation. We added a minor correction to our previously reported procedure. The previous procedure included infections by zero founders (i.e., the failure of infection due to stochastic variation in founder number) to single infections, resulting in a slight overestimation of the single infections; this was corrected. We confirmed that this correction only slightly changes the estimates of the mean founder number in the previous report by ∼1% (changed *λ* = 5.97 to *λ* = 5.93 for the first cell-to-cell transmission and *λ* = 5.02 to *λ* = 4.97 for the second cell-to-cell transmission).

### Estimation of the Number of Genomic RNA Molecules Introduced into Cells

To estimate the number of genomic RNA molecules introduced into each cell by electroporation, genomic RNA was labeled with [α-^32^P]GTP during transcription and then purified using Mini Quick Spin RNA Columns (Roche Applied Science, Penzberg, Germany). The purified RNA was quantified using a Qubit fluorometer and then introduced into protoplasts as described above. The inoculated protoplasts were washed with protoplast culture medium twice, with protoplast culture media containing 44 U/ml of micrococcal nuclease (TaKaRa Bio) four times, and finally with protoplast culture medium three times. The number of cells was then counted under a microscope. The number of RNA molecules associated with the cells was estimated based on the radioactivity. To estimate the amount of RNA molecules that were bound outside the cells but had not washed off, we mixed labeled RNA with protoplasts immediately after mock inoculation and estimated the amount after washing. We subtracted the amount of RNA that was associated outside and then calculated the amount of RNA that was introduced into the cell.

### Northern Blotting

Northern blotting was performed as described previously [34]. A probe named PPYP was used to detect the complementary RNA strand of TLPYFP. The probe corresponds to the 5′ part of the YFP coding region (523 nt) and its upstream 124 nt region of TLPYFP (Fig. 1A). The probe was labeled by incorporating DIG-UTP (Roche Applied Science) during transcription with T7 RNA polymerase. The hybridized probe was detected using DIG detection reagents (Roche Applied Science) and an LAS-3000 image analyzer (Fujifilm, Tokyo, Japan).

### Analysis of the Sequence-Tagged ToMV That Accumulated in Individual Cells

Cells infected with the sequence-tagged ToMV library were identified by observation under a fluorescence microscope (IX70; Olympus, Tokyo, Japan) and harvested into PCR tubes using glass capillaries connected to a CellTram vario microinjector (Eppendorf, Hamburg, Germany) and controlled by a micromanipulator (Narishige, Tokyo, Japan). Extracellular nucleic acids were digested using micrococcal nuclease (TaKaRa Bio) to eliminate contamination with inoculated transcripts that bound to the outside of the cell or viral RNA from broken cells. After inactivating micrococcal nuclease by the addition of EGTA, each cell was broken by shaking vigorously. RT-PCR amplification of the regions containing a 10-nucleotide sequence tag was then performed using PrimeScript One Step RT-PCR kit Ver.2 (TaKaRa Bio) and the oligo DNA primers To17 and To18. Each RT-PCR product was diluted with distilled H_2_O and subjected to nested PCR using PrimeSTAR HS polymerase (TaKaRa Bio) and the oligo DNA primer sets TagYF and TagGR. These primer sets contain 3–5 nucleotide tag sequences that were used to identify each cell sample. Equal amounts of the PCR products were mixed and analyzed using a GAIIx sequencer (Illumina, San Diego, California, US), and the resultant reads were redistributed to the corresponding samples based on the sample identification tags. The 10-nucleotide tag sequences detected at an incidence of <2% in each cell sample were discarded because of probable sequencing errors in the tag sequences, misdistribution of the reads caused by sequencing errors in the sample-identification tags, and/or wrong priming in the amplification step. The tag sequences detected in multiple samples at low frequencies were also discarded as contamination of the RNA samples with extracellular RNA.

### Characterization of the Less Fit TLPYFP Variant TLPYFPd2–8

Translation, complementary-strand RNA synthesis, and genomic and subgenomic RNA synthesis were examined using an in vitro translation/replication system with BYL lysates from evacuolated tobacco BY2 protoplasts [21,22]. Briefly, viral RNA was added to membrane-depleted BYL and translated for 1 h at 25°C. After stopping the translation with puromycin, the lysate was mixed with a membrane fraction of BYL and incubated at 15°C for 1 h. A replication reaction was then performed in the presence of NTPs for 1 h. Protein samples were harvested before the addition of the membranes, and western blotting was performed as described previously using antibodies raised against the helicase domain of the ToMV replication proteins [12]. RNA samples were prepared using phenol–chloroform extraction after the replication reaction. To detect the genomic RNA, replication reactions were performed in the presence of [α-^32^P]CTP, and RNase protection assays were performed using a nonlabeled P5′M probe. To detect the complementary-strand RNA, the replication reactions were performed in the absence of radiolabeled NTPs, and the RNase protection assay was performed using a ^32^P-labeled PPYP probe.

## Supporting Information

S1 Data Simulation results for RC generations at different parameter values. (XLS)

S2 Data Simulation results for founder numbers and Shannon entropy at different parameter values. (XLS)

S3 Data Simulation results for selection on *trans*-acting genes or elements at different conditions. (XLS)

S4 Data Simulation results for viral RNA accumulation of variants with different genomic RNA synthesis efficiencies. (XLS)

S5 Data Simulation results for viral RNA accumulation of variants with different RC formation efficiencies. (XLS)

S6 Data Simulation results for relative accumulation of a variant to a wild-type virus in 1,000 cells at different parameter values. (XLS)

S1 Movie Simulated course of single-cell infection. (MP4)

S2 Movie Simulated competition in a coinfected cell. (MP4)

S1 Fig A constraint in the adaptation of *trans*-acting genes or elements. (A) The shared use of *trans*-acting genes or elements among intracellular populations delays selection. (B) Infection with a small number of genomes counteracts the negative effect of share use by isolating the adaptive genomes from defective genomes in a stochastic manner, thereby enabling selection among intracellular populations.
(TIF)

S2 Fig Observation of complementary-strand RNA accumulation and gene expression over time. (A) Effect of inhibiting translation on complementary-strand RNA accumulation. TLPYFP-inoculated protoplasts were sampled at indicated time points (upper panel) or mixed with cycloheximide (CHX) at the indicated time points, incubated, and sampled at 24 hpi (lower panel). Complementary-strand RNA was detected using northern blotting. The relative band intensities to that of samples harvested at 16 hpi are shown below the panels. The slight increase in the amount of complementary-strand RNA after CHX treatment suggests that CHX treatment did not completely or immediately stop translation and/or that already translated viral replication proteins participated in the formation of additional RCs. The result eliminates the possibility that already formed RCs and the complementary-strand RNAs they contain are degraded. Note that a radioisotope-labeled probe was used rather than a DIG-labeled probe. (B) The observation of TLPCFP-inoculated cells at different time points after inoculation. Note that CFP fluorescence can be detected only at very low levels at 8 hpi.
(TIF)

S3 Fig Simulated frequencies of RCs belonging to different generations formed in an infected cell. (A) Simulation using the following parameter values: *E* = 5 × 10^3^, *R* = 3 × 10^4^, *p* = 3 × 10^–10^, and *d* = 1 × 10^–2^. (B) Simulation using the following parameter values: *E* = 5 × 10^3^, *R* = 3 × 10^4^, *p* = 3 × 10^–9^, and *d* = 1 × 10^–1^. (C) Simulation using the following parameter values: *E* = 5 × 10^3^, *R* = 3 × 10^4^, *p* = 3 × 10^–11^, and *d* = 1 × 10^–3^. The mean frequencies of RCs in simulated 100-cell infections are shown by bar graphs. Means ± SD of the 100-cell results for mean generation, median generation, and maximum generation are indicated. An R script used for the simulation is shown in S2 Text, and the data obtained by the authors are shown in S1 Data.
(TIF)

S4 Fig Examples of changes in the amounts of genomic RNA and RCs over time in single-cell infections simulated using different parameter values. (A) Simulation using the following parameter values: *E* = 5 × 10^3^, *R* = 3 × 10^4^, *p* = 3 × 10^–9^, and *d* = 1 × 10^–1^. (B) Simulation using the following parameter values: *E* = 5 × 10^3^, *R* = 3 × 10^4^, *p* = 3 × 10^–11^, and *d* = 1 × 10^–3^. Data are presented as described in Fig. 3A and 3B. For the number of genomic RNAs, the total amount at each time point is indicated by the relative area of the pie chart. The total amount of the genomic RNA differs in panels A and B; therefore, the amount of genomic RNA per area size differs between panels. A comparison of the RC accumulation kinetics for complementary-strand RNA in the simulation and experimentally (Fig. 2B) suggests that 1 h in an experiment corresponds to ∼1,600 and ∼2,800 units of time in (A) and (B), respectively. An R script S1 Text was used for simulations with changes in parameter values.
(TIF)

S5 Fig Evaluation of variation in founder number and inequality in progeny accumulation in actual infections. (A) The expected distribution of founder numbers assuming the Poisson process with a mean founder number of 5.0. (B) Tag sequences and their frequencies identified from each cell sample. Each pie chart corresponds to a cell sample, and an effect size *ω* from equal detection is shown under each pie chart. (C) Control experiments. In control 1, a mixture containing equal amounts of five differently tagged viral RNAs was used as a template for RT-PCR. In control 2, RNA extracted from 1 × 10^5^ protoplasts inoculated with a mixture containing equal amounts of the five RNAs and cultured for 24 h was used as the template. Effect sizes *ω* from “ideal” equal detection and that from control 1 to control 2, which would reflect the effect of accumulation bias, are shown. See S5 Text for more details.
(TIF)

S6 Fig EBPA assuming variation in RC formation efficiency. The accumulation of wild-type RNA (magenta) and co-inoculated variant RNAs with different RC formation efficiencies (green) in 1,000 cells was simulated. The relative accumulation of the variants is also shown in a table below the graph. Means ± SEs calculated using bootstrap analyses of simulated 1,000-cell infections are shown. An R script used for the simulation and the obtained data are shown in S13 Text and S5 Data, respectively.
(TIF)

S7 Fig The effect of changes in the parameter values *E*, *p*, and *d* at a fixed *R* on SVFN and SIPA. Simulated variations in founder number and progeny accumulation using parameter sets assuming a fixed *R* and variable *E*, *p*, and *d*. The inoculation of 1,000 cells was simulated for each set of parameters, and the results are summarized in two-dimensional histograms showing founder number on the *x*-axis and Shannon entropy on the *y*-axis (as shown in Fig. 3D). An R script used for the simulation and the obtained data are shown in S3 Text and S2 Data, respectively.
(TIF)

S8 Fig The effect of changes in the parameter values *E*, *p*, and *d* at a fixed *R* on EBPA. Simulated relative accumulation of a variant virus with 50% efficiency of genomic RNA synthesis compared with the WT virus using parameter sets assuming a fixed *R* and variable *E*, *p*, and *d*. Inoculations of 1,000 cells using a 1:1 mixture of the variant and WT viruses were simulated for each set of parameters, and the relative accumulation in the 1,000 cells is shown in pie charts. The accumulation ratios are also indicated below the pie charts. An R script used for the simulation and the obtained data are shown in S14 Text and S6 Data, respectively.
(TIF)

S9 Fig The effect of changes in the parameter values *E*, *p*, and *d* at a fixed *R* on the number of RC formation cycles. Simulated RC formation cycles. The frequencies of RCs belonging to each generation were simulated for 100 cells, and the mean frequencies are shown as a histogram (as described in S3 Fig.). The mean number of generation cycles is also indicated. An R script used for the simulation and the obtained data are shown in S2 Text and S1 Data, respectively.
(TIF)

S10 Fig Effects of changes in the values of parameters *R*, *p*, and *d* at a fixed *E* on SVFN and SIPA. The occurrences of SVFN and SIPA were tested for parameter sets assuming a fixed *E* and variable *R*, *p*, and *d*. The results are presented in the same way as in S7 Fig. An R script used for the simulation and the obtained data are shown in S3 Text and S2 Data, respectively.
(TIF)

S11 Fig Effects of changes in the values of parameters *R*, *p*, and *d* at a fixed *E* on EBPA. The occurrences of EBPA was tested for parameter sets assuming a fixed *E* and variable *R*, *p*, and *d*. The results are presented in the same way as in S8 Fig. An R script used for the simulation and the obtained data are shown in S14 Text and S6 Data, respectively.
(TIF)

S12 Fig Effects of changes in the values of parameters *R*, *p*, and *d* at a fixed *E* on the number of RC formation cycles. The frequencies of RCs belonging to each generation were simulated for parameter sets assuming a fixed *E* and variable *R*, *p*, and *d*. The results are presented in the same way as in S9 Fig. An R script used for the simulation and the obtained data are shown in S2 Text and S1 Data, respectively.
(TIF)

S13 Fig Spread of TLPYFP-CP in a tobacco leaf tissue. YFP-fluorescence images of three sites infected with TLPYFP-CP were obtained at indicated time points.
(TIF)

S14 Fig The positions of oligo DNA fragments on sequence-tagged TLPYFP genomes. (A) The oligo DNA fragments used to construct a library of sequence-tagged TLPYFP. (B) The oligo DNA fragments used to sequence the tags. Open squares indicate YFP coding regions, small black boxes represent the 10-nucleotide random sequence tag, and arrows denote the position of the oligo DNA fragments and their directions.
(TIF)

S1 Table Detection frequencies of the tag sequences. Detection frequencies of tag sequences from each infected cell sample and control samples are shown.
(DOC)

S2 Table Expected numbers of infected and uninfected cells at different *E* values. Using the simulation model for cell infection, inoculations of 10,000 cells were simulated at different *E* values and fixed values for *R*, *p*, and *d* (*R* = 3 × 10^4^, *p* = 3 × 10^–10^, and *d* = 1 × 10^–2^). The expected numbers of infected (founder number ≥ 1) and uninfected (founder number = 0) cells out of the 10,000 cells are shown.
(DOC)

S3 Table The oligo DNA fragments used in this study. (DOC)

S4 Table *N*_e_-decreasing effect of SIPA at different founder numbers. (DOC)

S1 Text An R script used to generate Fig. 3ABC. The authors recommend the interested readers try this script.
(DOC)

S2 Text An R script used to obtain the data for S3 Fig., S9 Fig., and S12 Fig.
(DOC)

S3 Text An R script used to obtain the data for Fig. 3D–3F, S7 Fig., and S10 Fig.
(DOC)

S4 Text The effect of sampling errors in the analysis of sequence-tagged virus. (DOC)

S5 Text Quantification of the variance in progeny accumulation. (DOC)

S6 Text An R script used to obtain the data for Fig. 5C and 5D. (DOC)

S7 Text The adaptation-enhancing effects of SVFN and SIPA: The effective population size. (DOC)

S8 Text An R script used to simulate co-inoculation of 1,000 cells with a wild-type virus and a variant that synthesizes viral genomic RNA at 50% efficiency to the wild-type virus. (DOC)

S9 Text An R script used to generate Fig. 6A and 6B. (DOC)

S10 Text An R script used to generate Fig. 6C. (DOC)

S11 Text An R script used to obtain the data for Fig. 6D. (DOC)

S12 Text Another R script used to obtain the data for Fig. 6D. (DOC)

S13 Text An R script used to obtain the data for S6 Fig.
(DOC)

S14 Text An R script used to obtain the data for S8 Fig. and S11 Fig.
(DOC)

## Abbreviations

CaMV: cauliflower mosaic virus
CCD: charge-coupled device
CFP: cyan fluorescent protein
CP: coat protein
EBPA: enhanced bias in progeny accumulation
FHV: Flock House virus
HCV: hepatitis C virus
HIV: human immunodeficiency virus
hpi: hours postinoculation
HSV: herpes simplex virus
JSBWMV: Japanese soil-borne wheat mosaic virus
MP: cell-to-cell movement protein
NLS: nuclear localization signal
PFU: plaque forming unit
PRV: pseudorabies virus
RCs: replication complexes
RPA: RNase protection assay
RT-PCR: reverse transcription–polymerase chain reaction
SD: standard deviation
SE: standard error
SIPA: stochastic inequality in progeny accumulation
SVFN: stochastic variation in founder number
TMV: tobacco mosaic virus
ToMV: tomato mosaic virus
UTR: untranslated region
WT: wild-type
YFP: yellow fluorescent protein
