# Supplementary figures and images for: Viruses Roll the Dice: The Stochastic Behavior of Viral Genome Molecules Accelerates Viral Adaptation at the Cell and Tissue Levels
Source: PLoS Biol. 2015 Mar 17;13(3):e1002094. doi: 10.1371/journal.pbio.1002094 (PMC4364534; doi:10.1371/journal.pbio.1002094)

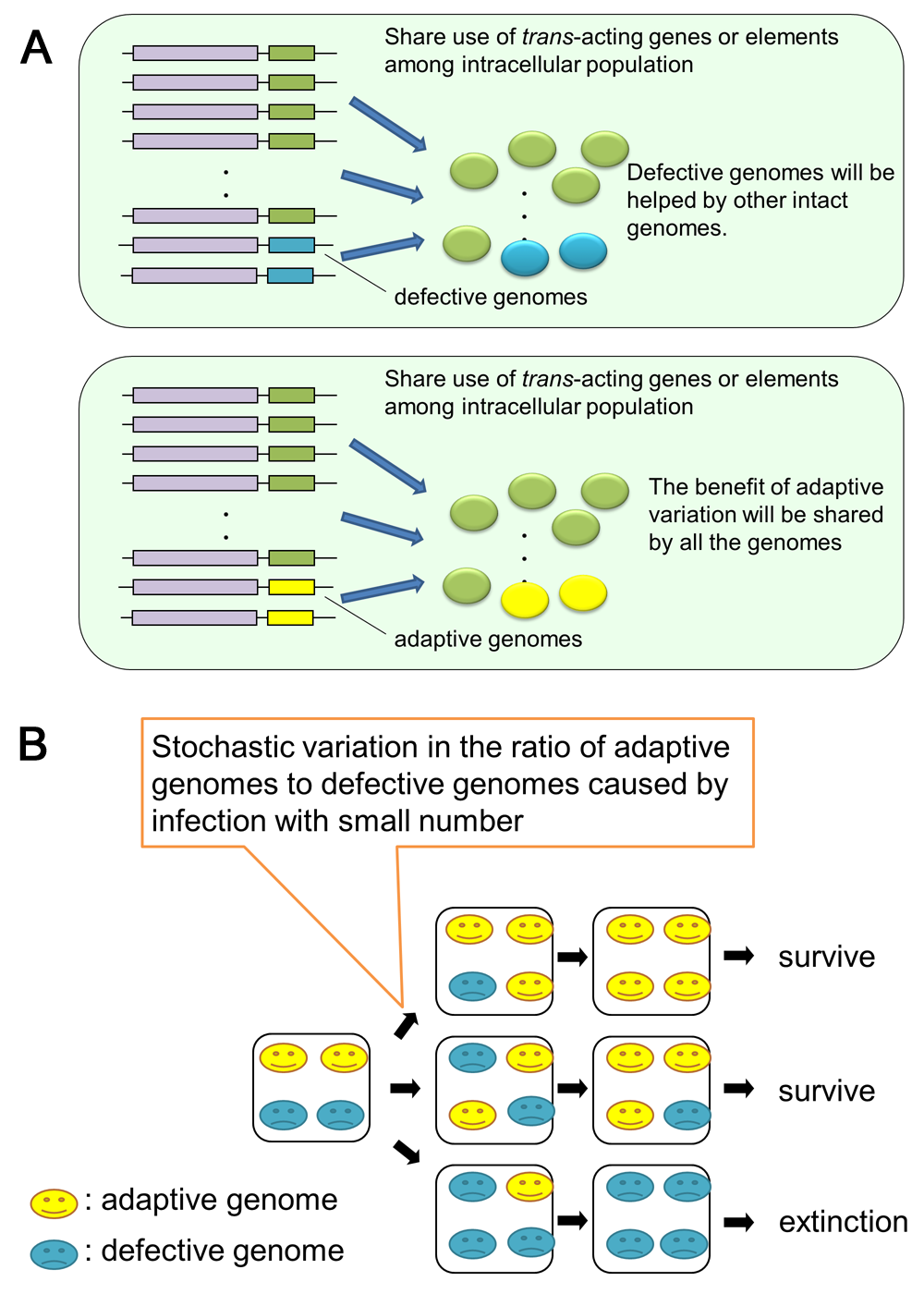

Supplement: S1 Fig — (A) The shared use of trans-acting genes or elements among intracellular populations delays selection. (B) Infection with a small number of genomes counteracts the negative effect of share use by isolating the adaptive genomes from defective genomes in a stochastic manner, thereby enabling selection among intracellular populations. (TIF) [file pbio.1002094.s009.tif]

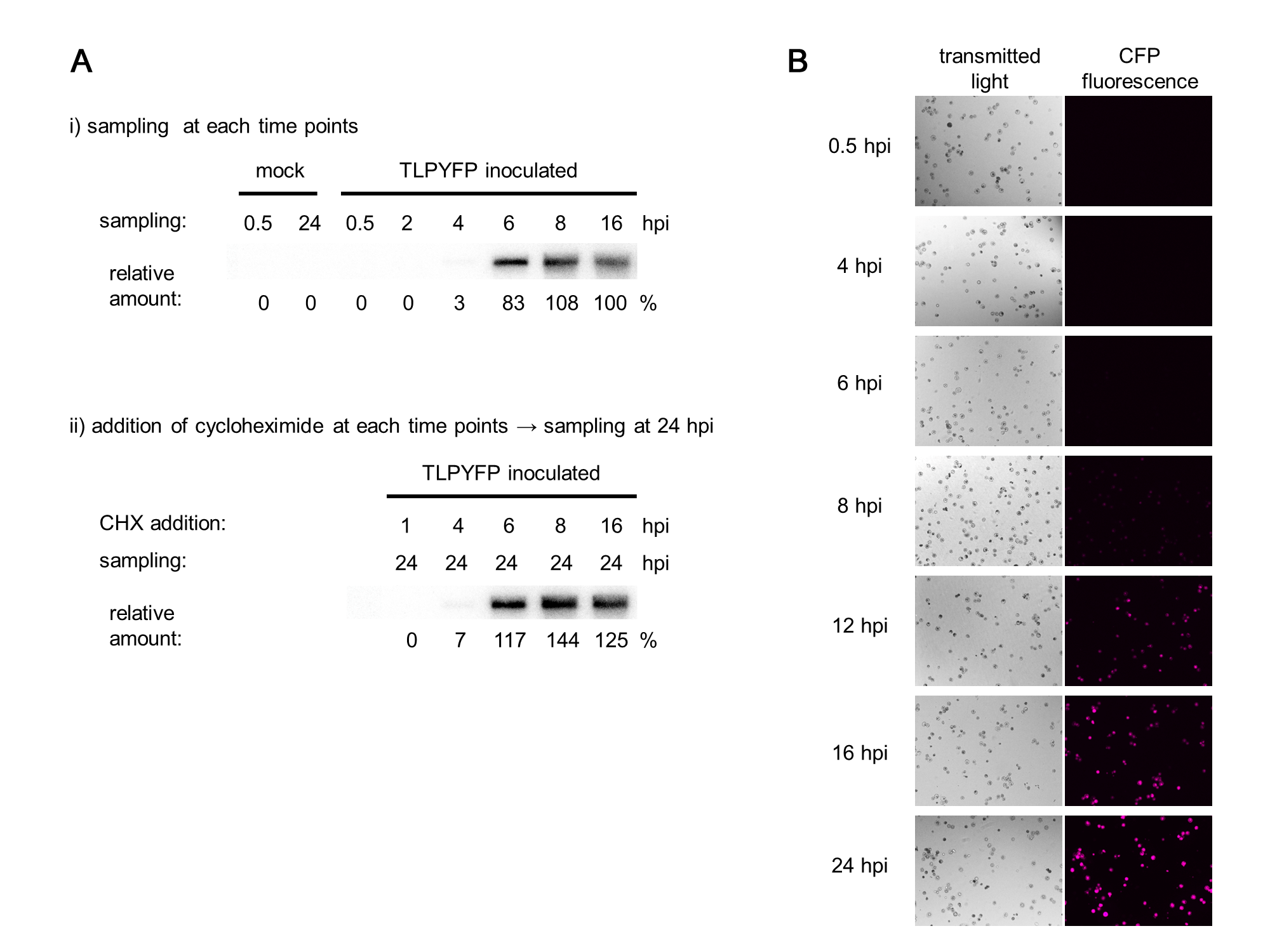

Supplement: S2 Fig — (A) Effect of inhibiting translation on complementary-strand RNA accumulation. TLPYFP-inoculated protoplasts were sampled at indicated time points (upper panel) or mixed with cycloheximide (CHX) at the indicated time points, incubated, and sampled at 24 hpi (lower panel). Complementary-strand RNA was detected using northern blotting. The relative band intensities to that of samples harvested at 16 hpi are shown below the panels. The slight increase in the amount of complementary-strand RNA after CHX treatment suggests that CHX treatment did not completely or immediately stop translation and/or that already translated viral replication proteins participated in the formation of additional RCs. The result eliminates the possibility that already formed RCs and the complementary-strand RNAs they contain are degraded. Note that a radioisotope-labeled probe was used rather than a DIG-labeled probe. (B) The observation of TLPCFP-inoculated cells at different time points after inoculation. Note that CFP fluorescence can be detected only at very low levels at 8 hpi. (TIF) [file pbio.1002094.s010.tif]

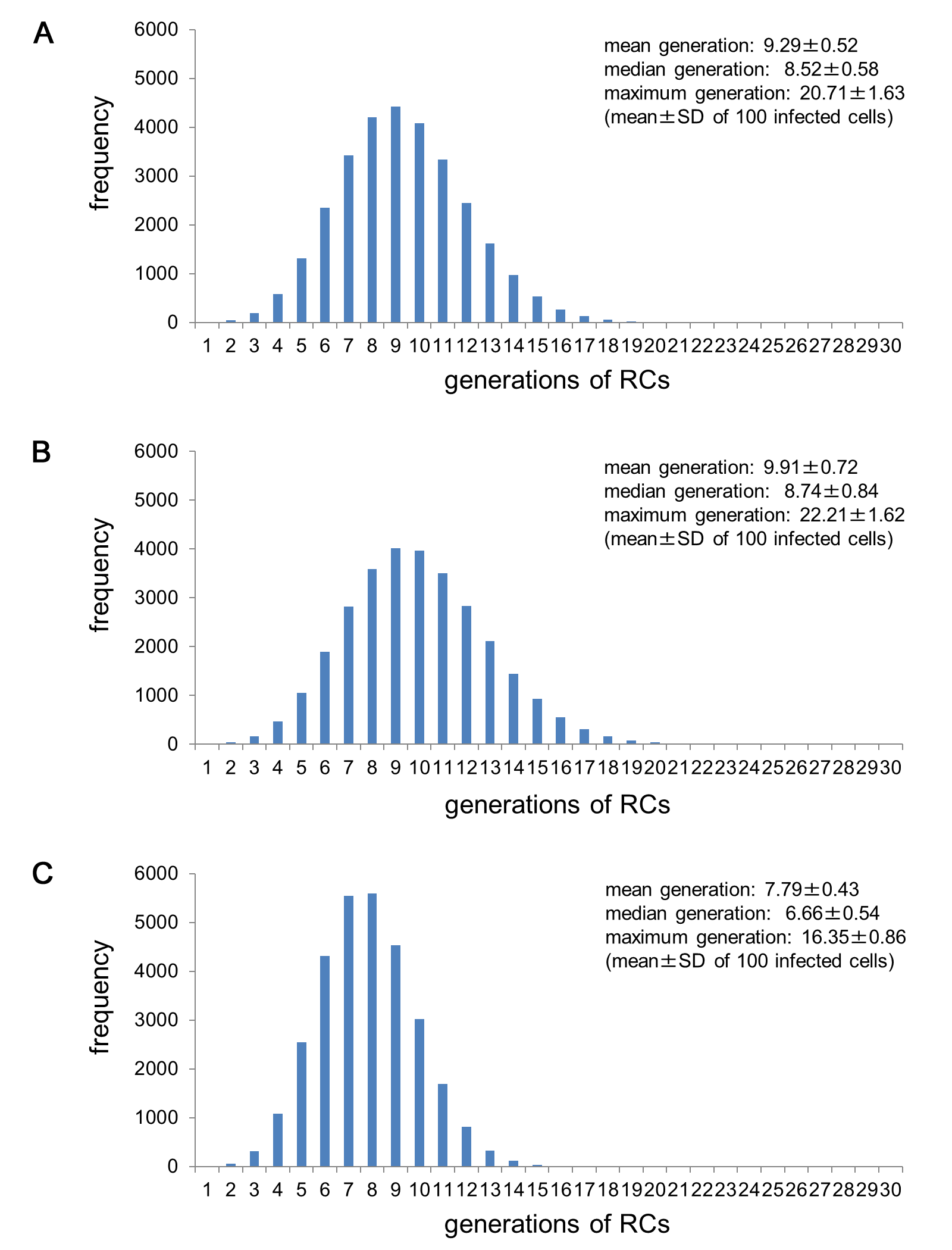

Supplement: S3 Fig — (A) Simulation using the following parameter values: E = 5 × 10^3^, R = 3 × 10^4^, p = 3 × 10^–10^, and d = 1 × 10^–2^. (B) Simulation using the following parameter values: E = 5 × 10^3^, R = 3 × 10^4^, p = 3 × 10^–9^, and d = 1 × 10^–1^. (C) Simulation using the following parameter values: E = 5 × 10^3^, R = 3 × 10^4^, p = 3 × 10^–11^, and d = 1 × 10^–3^. The mean frequencies of RCs in simulated 100-cell infections are shown by bar graphs. Means ± SD of the 100-cell results for mean generation, median generation, and maximum generation are indicated. An R script used for the simulation is shown in S2 Text, and the data obtained by the authors are shown in S1 Data. (TIF) [file pbio.1002094.s011.tif]

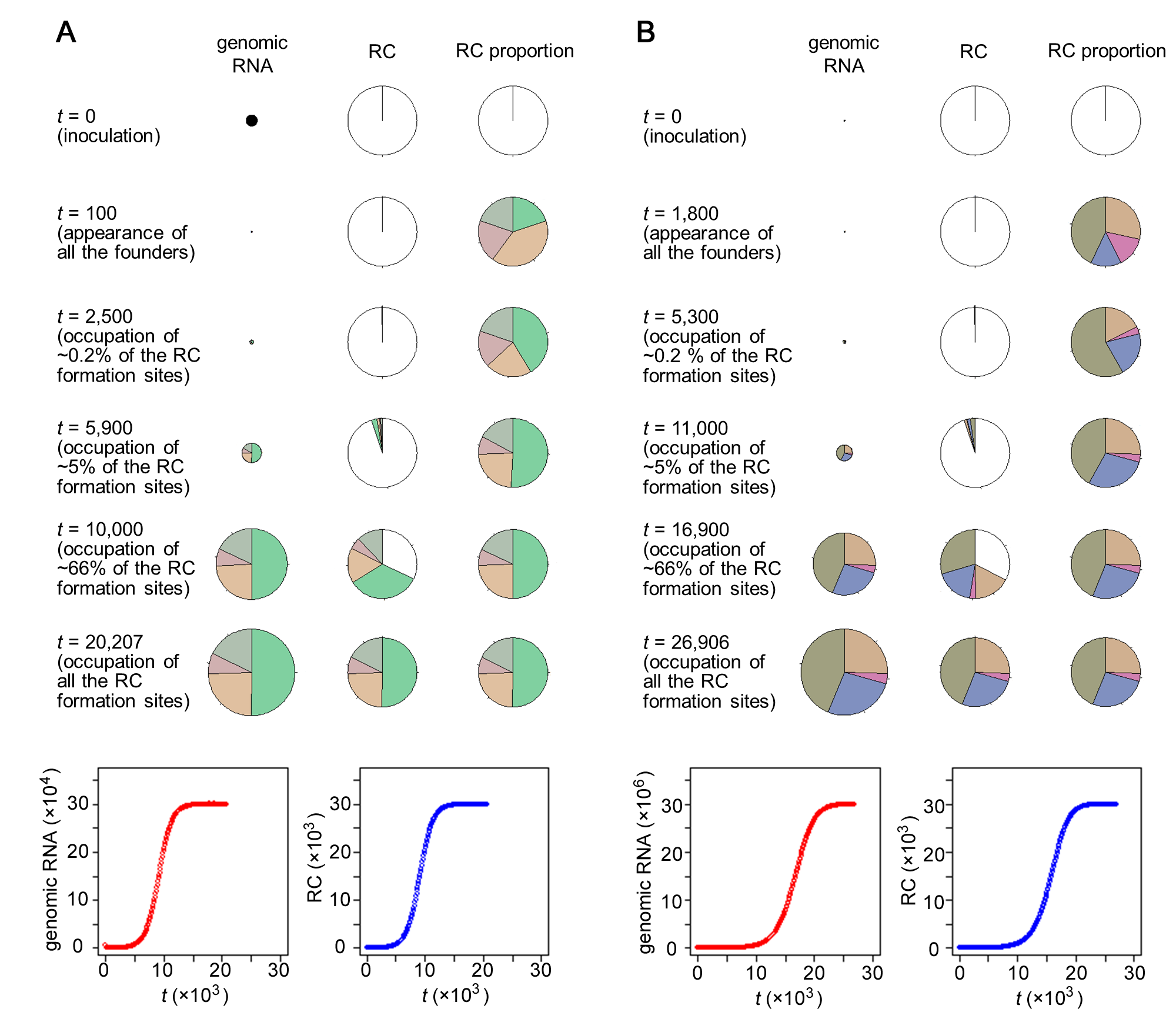

Supplement: S4 Fig — (A) Simulation using the following parameter values: E = 5 × 10^3^, R = 3 × 10^4^, p = 3 × 10^–9^, and d = 1 × 10^–1^. (B) Simulation using the following parameter values: E = 5 × 10^3^, R = 3 × 10^4^, p = 3 × 10^–11^, and d = 1 × 10^–3^. Data are presented as described in Fig. 3A and 3B. For the number of genomic RNAs, the total amount at each time point is indicated by the relative area of the pie chart. The total amount of the genomic RNA differs in panels A and B; therefore, the amount of genomic RNA per area size differs between panels. A comparison of the RC accumulation kinetics for complementary-strand RNA in the simulation and experimentally (Fig. 2B) suggests that 1 h in an experiment corresponds to ∼1,600 and ∼2,800 units of time in (A) and (B), respectively. An R script S1 Text was used for simulations with changes in parameter values. (TIF) [file pbio.1002094.s012.tif]

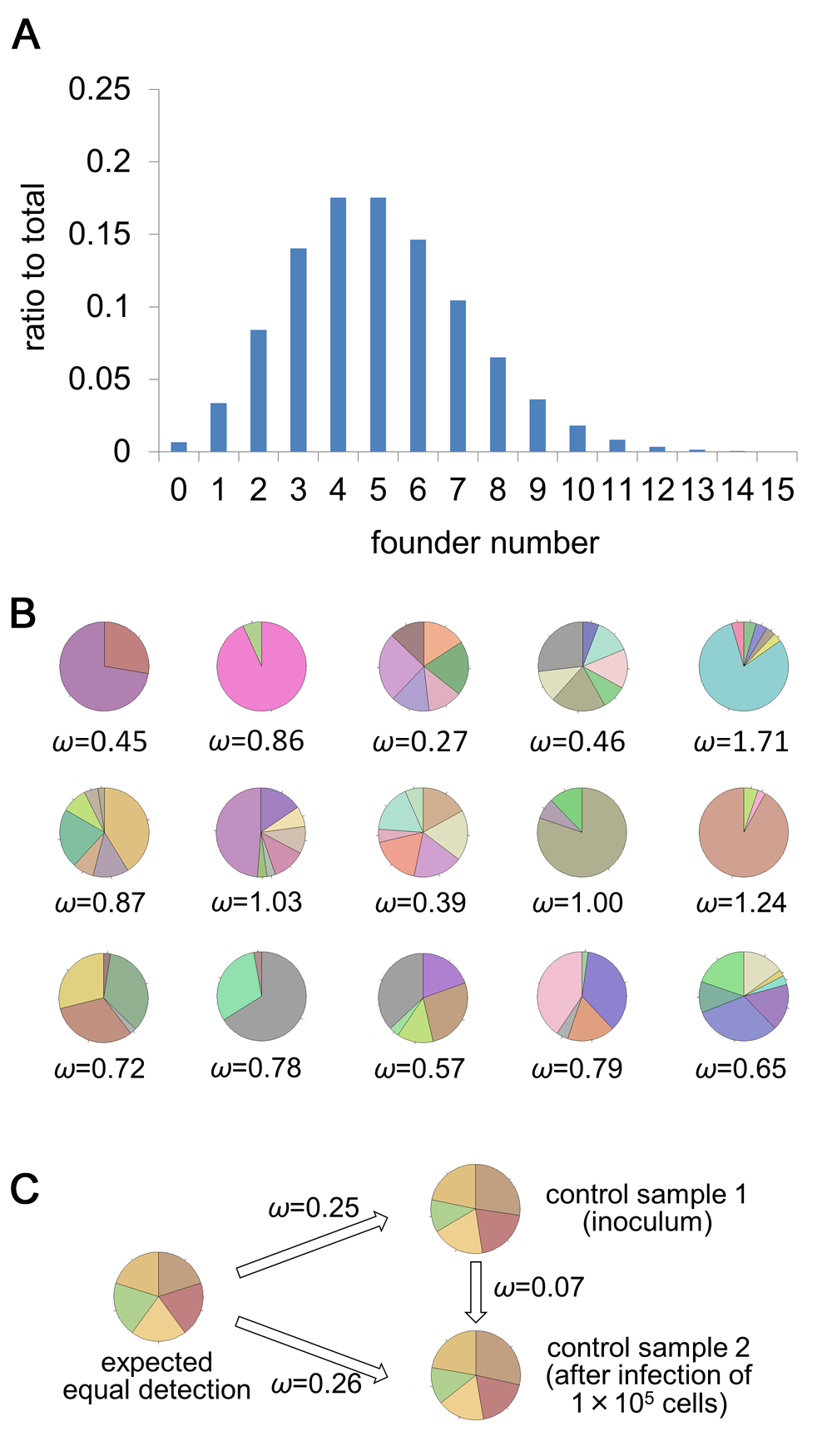

Supplement: S5 Fig — (A) The expected distribution of founder numbers assuming the Poisson process with a mean founder number of 5.0. (B) Tag sequences and their frequencies identified from each cell sample. Each pie chart corresponds to a cell sample, and an effect size ω from equal detection is shown under each pie chart. (C) Control experiments. In control 1, a mixture containing equal amounts of five differently tagged viral RNAs was used as a template for RT-PCR. In control 2, RNA extracted from 1 × 10^5^ protoplasts inoculated with a mixture containing equal amounts of the five RNAs and cultured for 24 h was used as the template. Effect sizes ω from “ideal” equal detection and that from control 1 to control 2, which would reflect the effect of accumulation bias, are shown. See S5 Text for more details. (TIF) [file pbio.1002094.s013.tif]

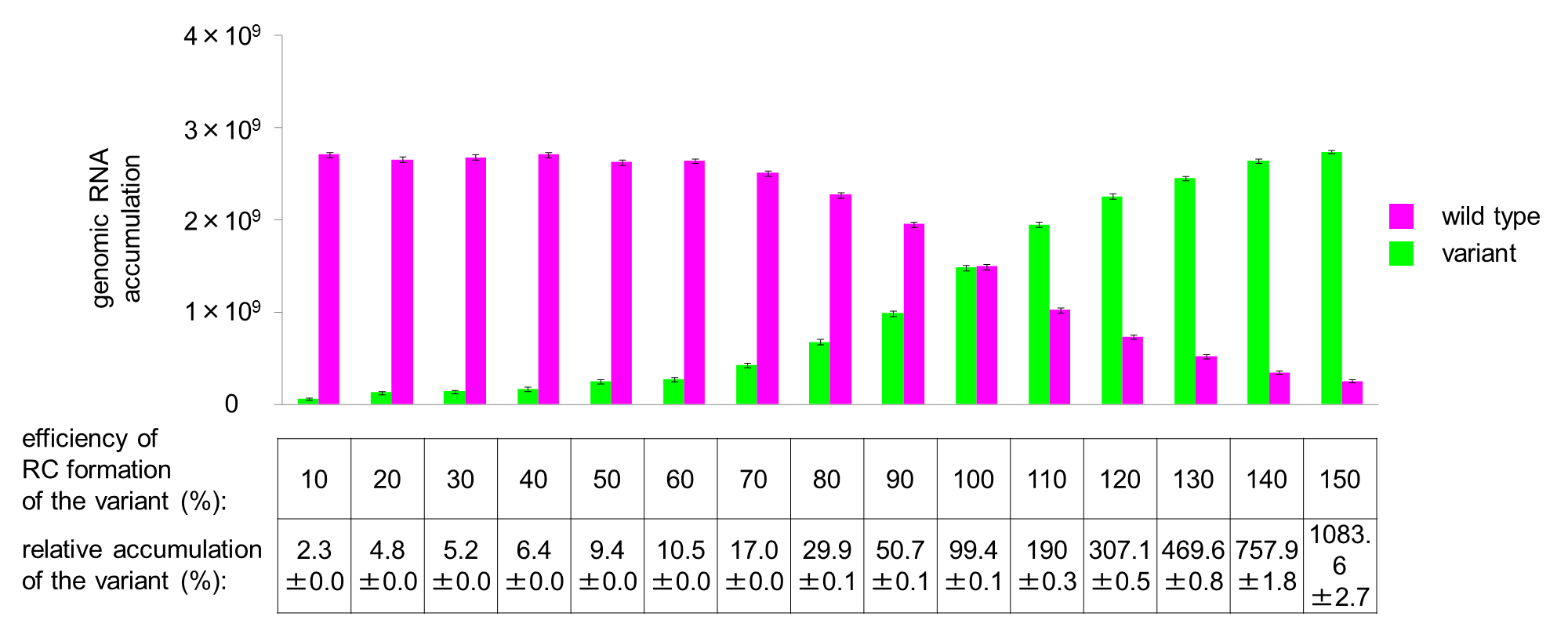

Supplement: S6 Fig — The accumulation of wild-type RNA (magenta) and co-inoculated variant RNAs with different RC formation efficiencies (green) in 1,000 cells was simulated. The relative accumulation of the variants is also shown in a table below the graph. Means ± SEs calculated using bootstrap analyses of simulated 1,000-cell infections are shown. An R script used for the simulation and the obtained data are shown in S13 Text and S5 Data, respectively. (TIF) [file pbio.1002094.s014.tif]

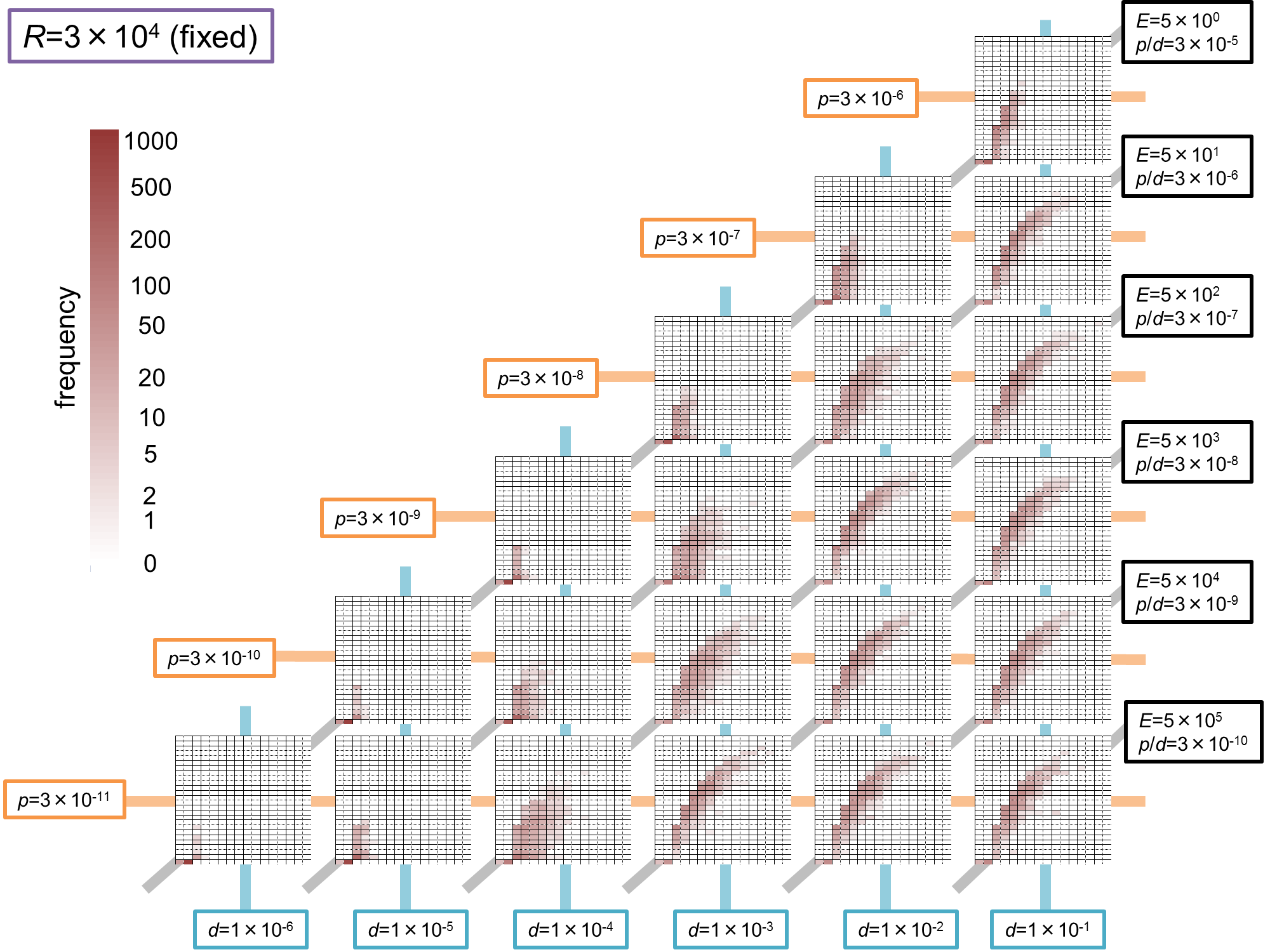

Supplement: S7 Fig — Simulated variations in founder number and progeny accumulation using parameter sets assuming a fixed R and variable E, p, and d. The inoculation of 1,000 cells was simulated for each set of parameters, and the results are summarized in two-dimensional histograms showing founder number on the x-axis and Shannon entropy on the y-axis (as shown in Fig. 3D). An R script used for the simulation and the obtained data are shown in S3 Text and S2 Data, respectively. (TIF) [file pbio.1002094.s015.tif]

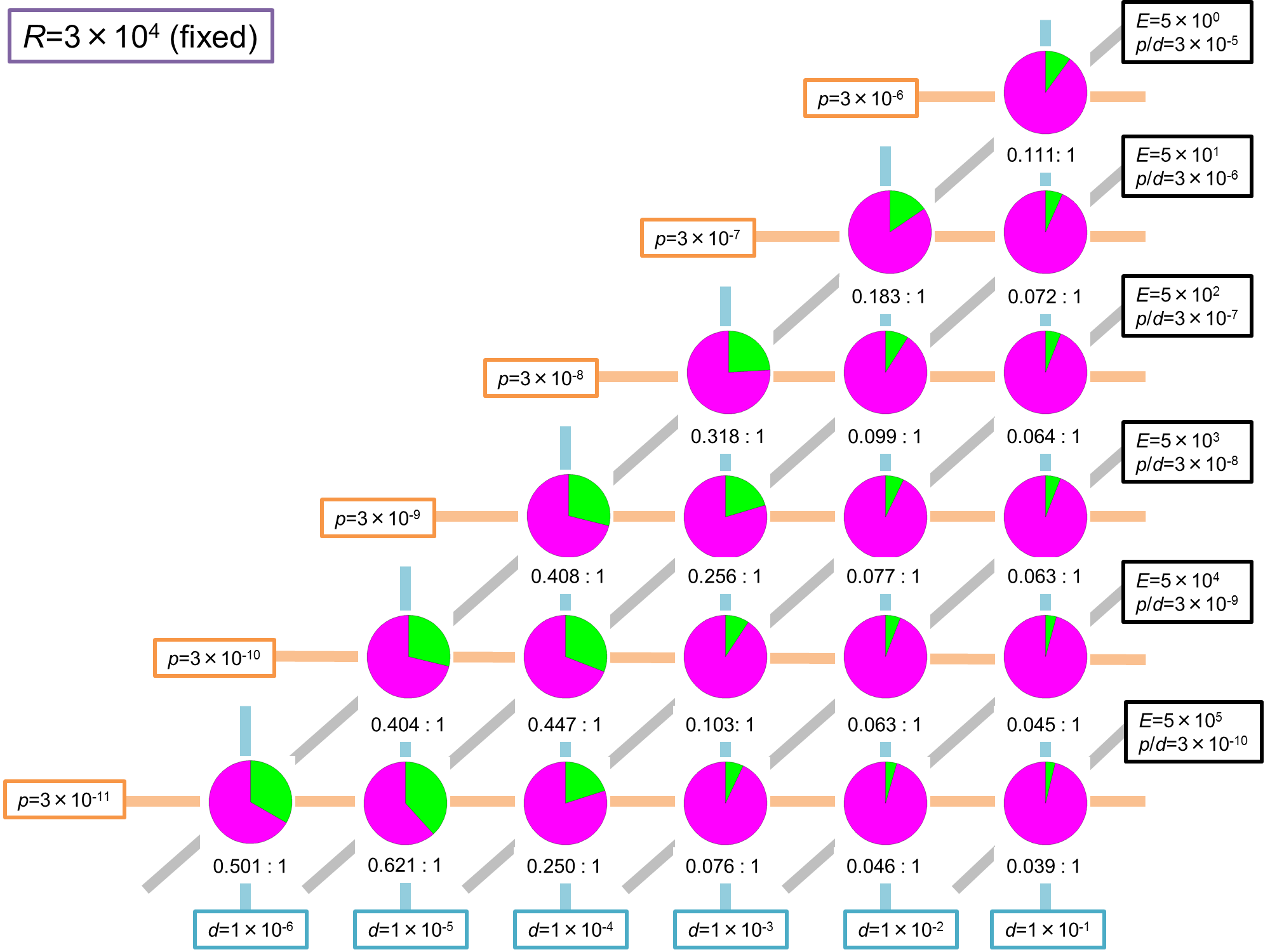

Supplement: S8 Fig — Simulated relative accumulation of a variant virus with 50% efficiency of genomic RNA synthesis compared with the WT virus using parameter sets assuming a fixed R and variable E, p, and d. Inoculations of 1,000 cells using a 1:1 mixture of the variant and WT viruses were simulated for each set of parameters, and the relative accumulation in the 1,000 cells is shown in pie charts. The accumulation ratios are also indicated below the pie charts. An R script used for the simulation and the obtained data are shown in S14 Text and S6 Data, respectively. (TIF) [file pbio.1002094.s016.tif]

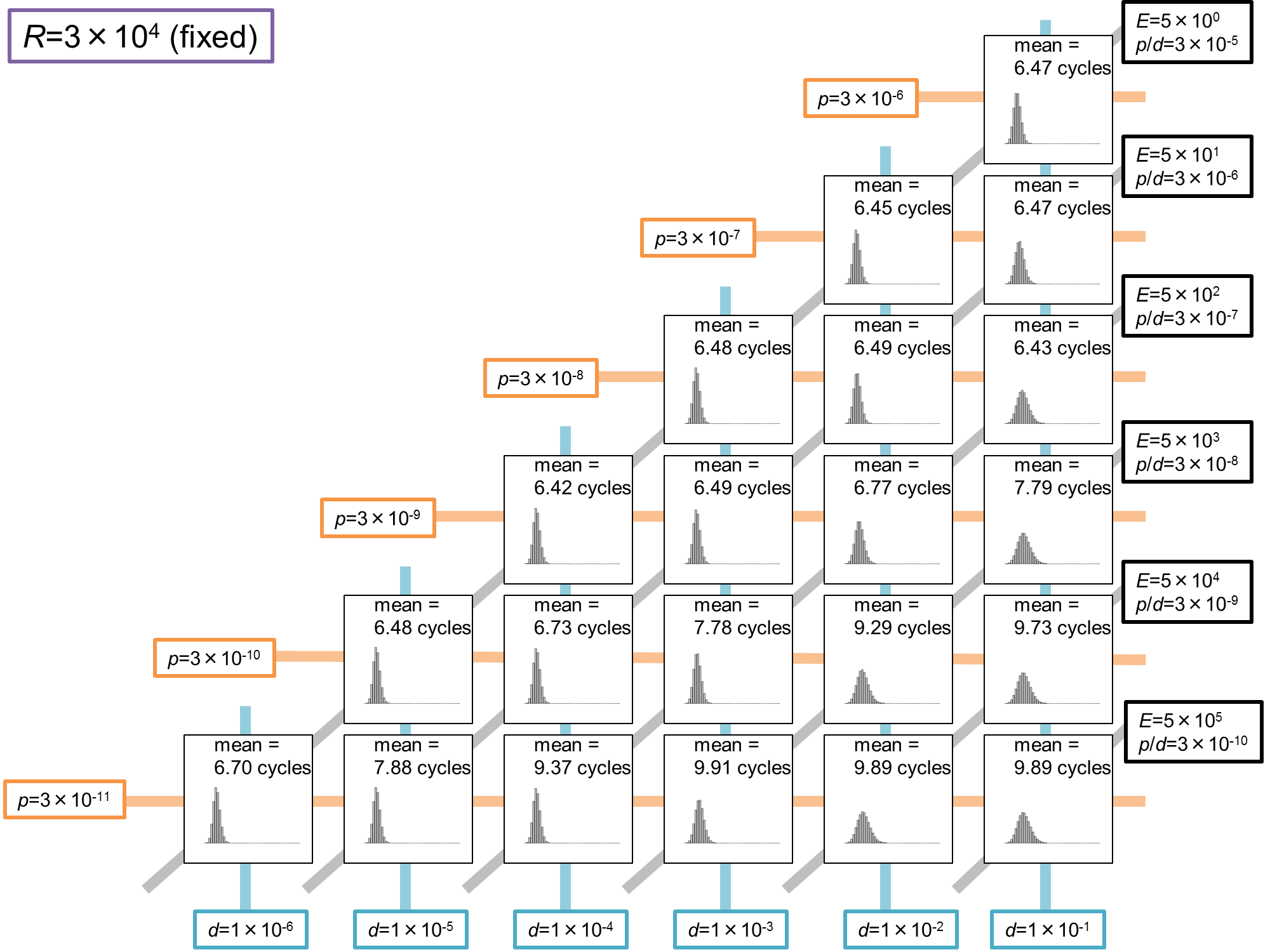

Supplement: S9 Fig — Simulated RC formation cycles. The frequencies of RCs belonging to each generation were simulated for 100 cells, and the mean frequencies are shown as a histogram (as described in S3 Fig.). The mean number of generation cycles is also indicated. An R script used for the simulation and the obtained data are shown in S2 Text and S1 Data, respectively. (TIF) [file pbio.1002094.s017.tif]

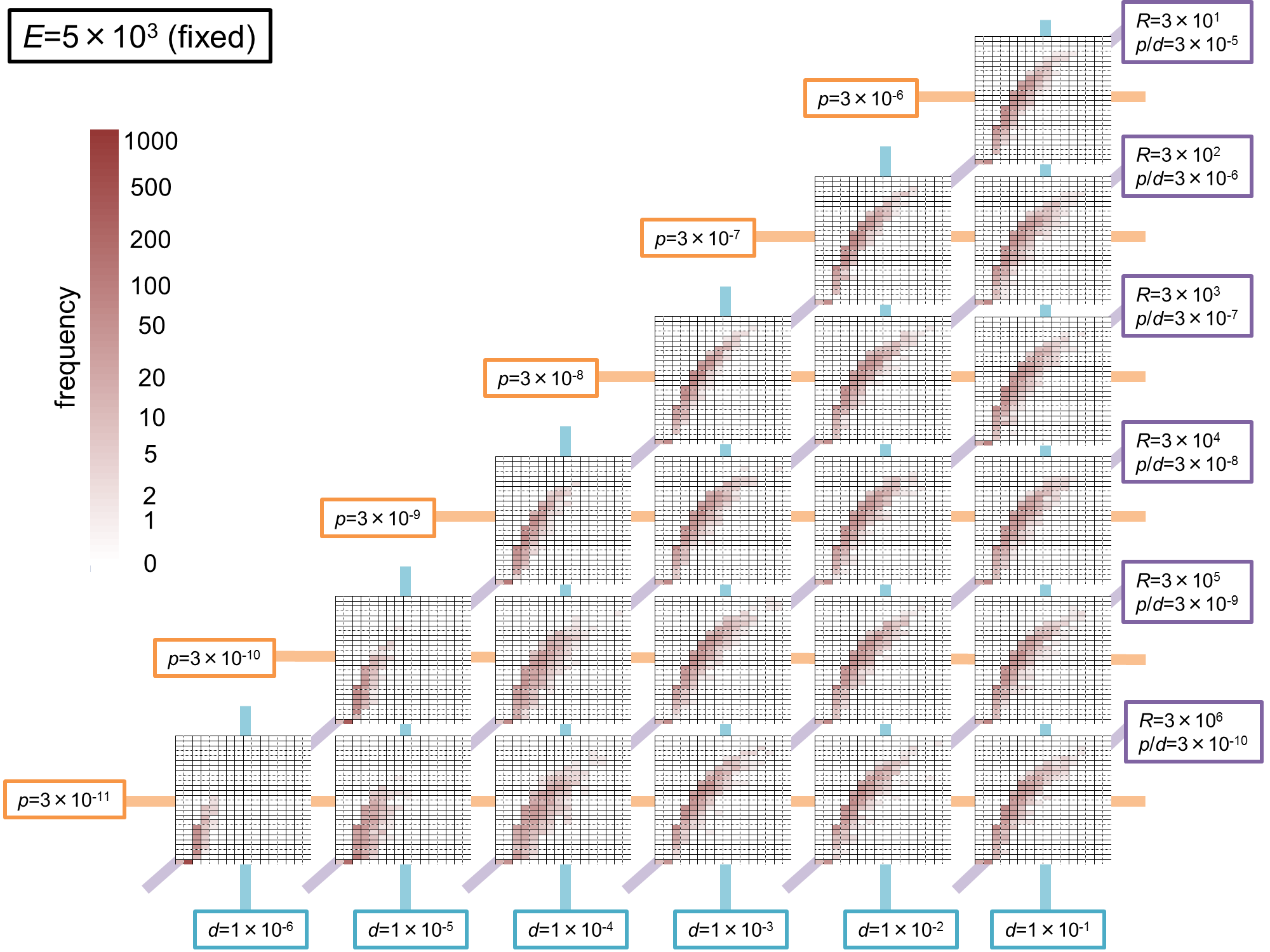

Supplement: S10 Fig — The occurrences of SVFN and SIPA were tested for parameter sets assuming a fixed E and variable R, p, and d. The results are presented in the same way as in S7 Fig. An R script used for the simulation and the obtained data are shown in S3 Text and S2 Data, respectively. (TIF) [file pbio.1002094.s018.tif]

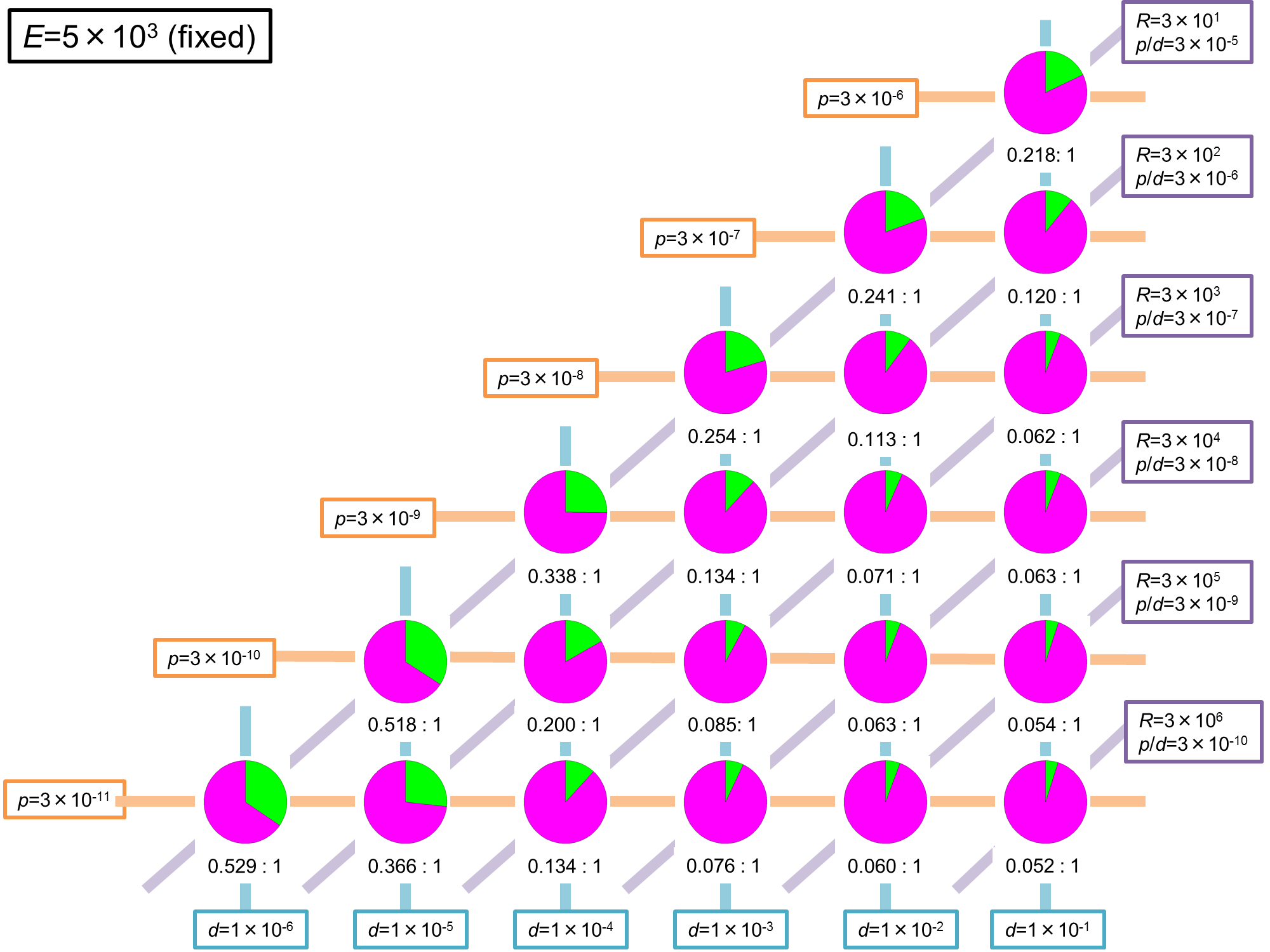

Supplement: S11 Fig — The occurrences of EBPA was tested for parameter sets assuming a fixed E and variable R, p, and d. The results are presented in the same way as in S8 Fig. An R script used for the simulation and the obtained data are shown in S14 Text and S6 Data, respectively. (TIF) [file pbio.1002094.s019.tif]

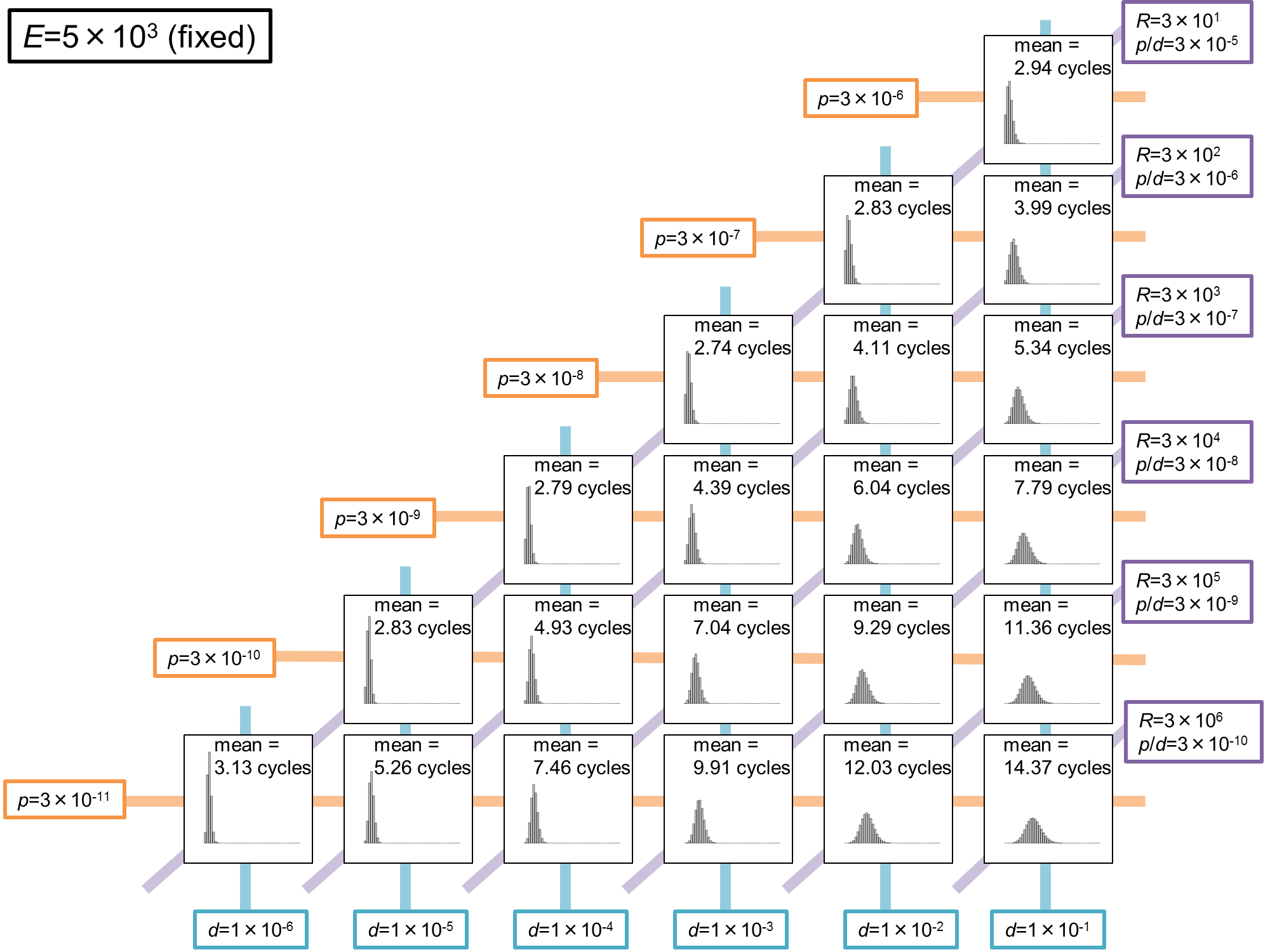

Supplement: S12 Fig — The frequencies of RCs belonging to each generation were simulated for parameter sets assuming a fixed E and variable R, p, and d. The results are presented in the same way as in S9 Fig. An R script used for the simulation and the obtained data are shown in S2 Text and S1 Data, respectively. (TIF) [file pbio.1002094.s020.tif]

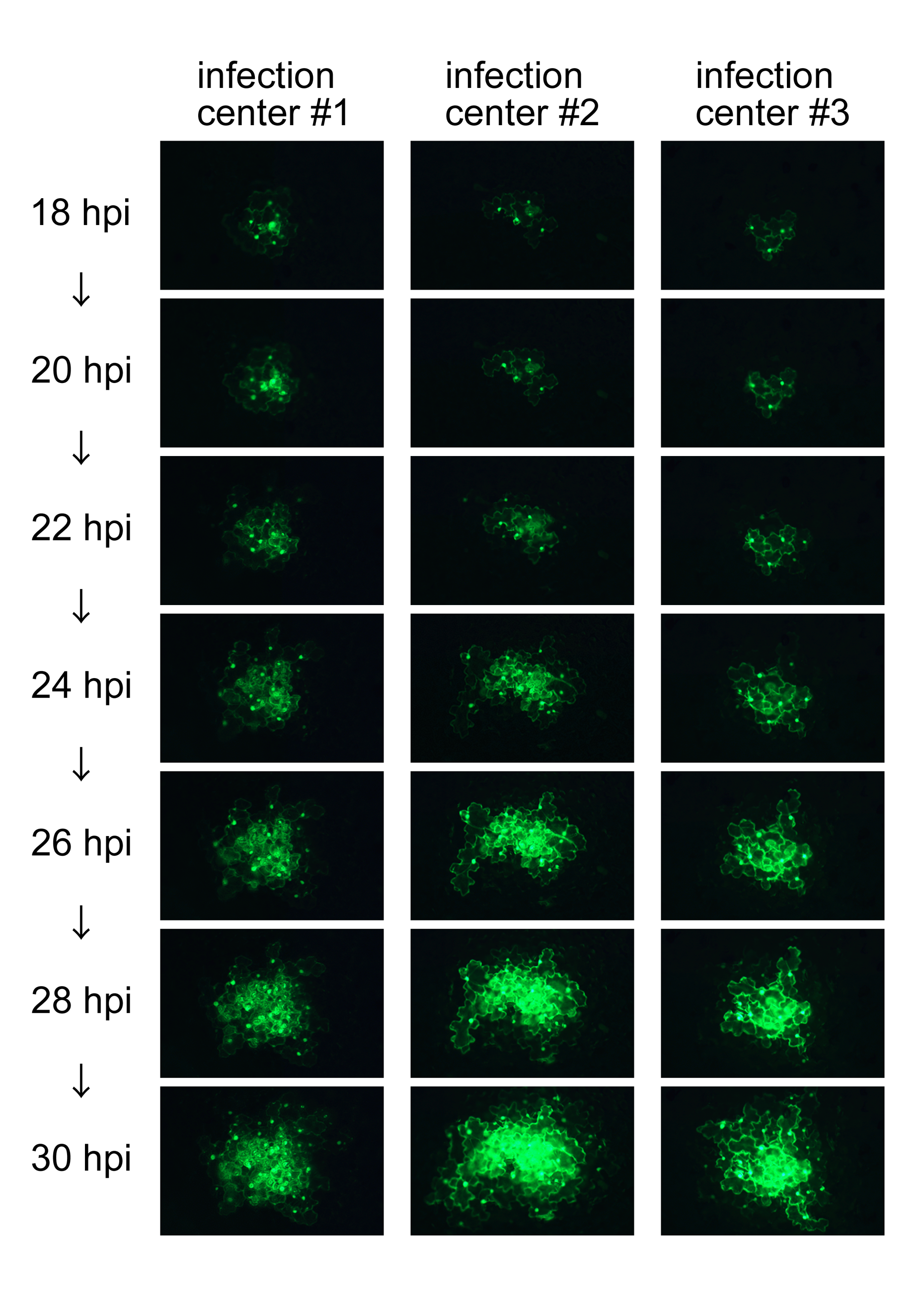

Supplement: S13 Fig — YFP-fluorescence images of three sites infected with TLPYFP-CP were obtained at indicated time points. (TIF) [file pbio.1002094.s021.tif]

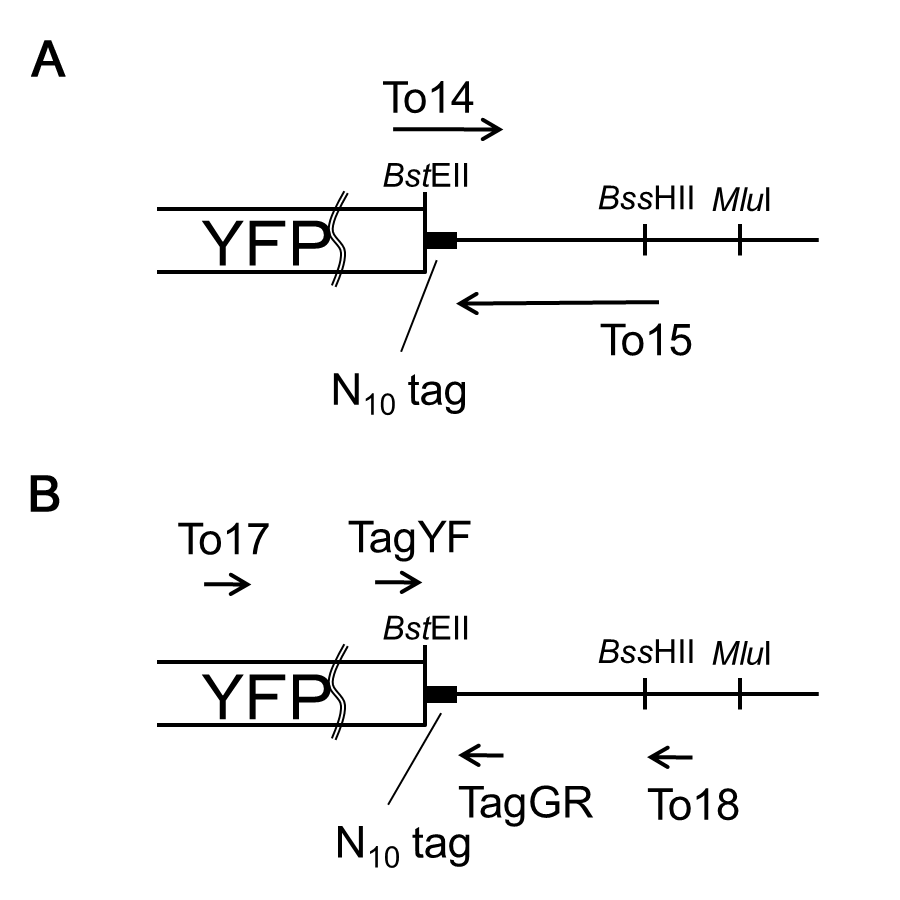

Supplement: S14 Fig — (A) The oligo DNA fragments used to construct a library of sequence-tagged TLPYFP. (B) The oligo DNA fragments used to sequence the tags. Open squares indicate YFP coding regions, small black boxes represent the 10-nucleotide random sequence tag, and arrows denote the position of the oligo DNA fragments and their directions. (TIF) [file pbio.1002094.s022.tif]
