# Supplementary material for: Viruses Roll the Dice: The Stochastic Behavior of Viral Genome Molecules Accelerates Viral Adaptation at the Cell and Tissue Levels
Source: PLoS Biol. 2015 Mar 17;13(3):e1002094. doi: 10.1371/journal.pbio.1002094 (PMC4364534; doi:10.1371/journal.pbio.1002094)
Supplement: S1 Table — Detection frequencies of tag sequences from each infected cell sample and control samples are shown. (DOC) [file pbio.1002094.s023.doc]

**S1 Table.** Detection frequencies of tag sequences

| sample | detected tag sequence | tag ID code | frequency | total frequency for each cell |
| --- | --- | --- | --- | --- |
| cell #1 | atggagactg | 1a | 61,585 | 223,057 |
|  | gatgtgcacc | 1b | 161,472 |  |
| cell #2 | catctcgtat | 2a | 155,858 | 167,255 |
|  | tgcgtattgt | 2b | 11,397 |  |
| cell #3 | acagtgccat | 3a | 31,989 | 201,812 |
|  | cggcgatccg | 3b | 40,050 |  |
|  | ggaatctgta | 3c | 25,030 |  |
|  | ggcccgaacc | 3d | 28,186 |  |
|  | tctgtacact | 3e | 50,608 |  |
|  | tttgtaagag | 3f | 25,949 |  |
| cell #4 | ataacacgaa | 4a | 13,955 | 238,791 |
|  | cgtaaaatgt | 4b | 30,919 |  |
|  | gcctgtggtc | 4c | 33,864 |  |
|  | tacattgaac | 4d | 21,045 |  |
|  | tctattaaat | 4e | 47,283 |  |
|  | tctgctgtca | 4f | 27,782 |  |
|  | tggatagcgg | 4g | 63,943 |  |
| cell #5 | agggggtgac | 5a | 4,276 | 95,826 |
|  | atgttaatgc | 5b | 3,919 |  |
|  | cccctacacc | 5c | 3,310 |  |
|  | gtaggtgagg | 5d | 3,130 |  |
|  | ttaaagcgtt | 5e | 76,897 |  |
|  | tttggtgtat | 5f | 4,294 |  |
| cell #6 | aaaaattata | 6a | 59,905 | 145,621 |
|  | aaccgagcgt | 6b | 18,877 |  |
|  | cactaggact | 6c | 11,256 |  |
|  | cgtagccccg | 6d | 31,362 |  |
|  | gattcggaga | 6e | 13,413 |  |
|  | gcccaaataa | 6f | 7,442 |  |
|  | tatcctcgcc | 6g | 3,366 |  |

**S1Table. Detection frequencies of tag sequences (continued)**

| sample | detected tag sequence | tag ID code | frequency | total frequency for each cell |
| --- | --- | --- | --- | --- |
| cell #7 | agcgttgtta | 7a | 25,181 | 162,954 |
|  | attttttccc | 7b | 12,017 |  |
|  | cgggtttggc | 7c | 16,150 |  |
|  | ggatggcagc | 7d | 19,498 |  |
|  | tcattacttc | 7e | 5,044 |  |
|  | tgcgggatta | 7f | 5,635 |  |
|  | tttcccggcg | 7g | 79,429 |  |
| cell #8 | aatggccctt | 8a | 48,422 | 284,781 |
|  | acgtagatag | 8b | 51,972 |  |
|  | ataatagttt | 8c | 51,324 |  |
|  | ctatgttatc | 8d | 51,761 |  |
|  | gcgccaaggg | 8e | 13,462 |  |
|  | ggaaatgtgt | 8f | 49,132 |  |
|  | gttattccaa | 8g | 18,708 |  |
| cell #9 | atacgcaaat | 9a | 101,193 | 126,197 |
|  | ggttgctctc | 9b | 9,595 |  |
|  | tgctctttga | 9c | 15,409 |  |
| cell #10 | aacgtagcaa | 10a | 2,329 | 44,922 |
|  | ggggcaaagt | 10b | 1,274 |  |
|  | tccgaagctt | 10c | 41,319 |  |
| cell #11 | agggctgtta | 11a | 6,724 | 266,776 |
|  | ctggacggtt | 11b | 92,654 |  |
|  | gtagcgttgt | 11c | 6,229 |  |
|  | gttaatcacg | 11d | 84,138 |  |
|  | tcttcagcca | 11e | 77,031 |  |
| cell #12 | tgtcagtgca | 12a | 112,904 | 170,676 |
|  | tgtccttgtt | 12b | 52,985 |  |
|  | ttgggccctc | 12c | 4,787 |  |

**S1 Table. Detection frequencies of tag sequences (continued)**

| sample | detected tag sequence | tag ID code | frequency | total frequency for each cell |
| --- | --- | --- | --- | --- |
| cell #13 | aagcgggctc | 13a | 83,904 | 430,229 |
|  | agcaaacttg | 13b | 115,522 |  |
|  | aggccgagga | 13c | 55,979 |  |
|  | atatactaca | 13d | 15,729 |  |
|  | tagtcttcgg | 13e | 159,095 |  |
| cell #14 | aaccgtgaag | 14a | 5,607 | 253,424 |
|  | atgcgtccct | 14b | 90,731 |  |
|  | ctgtgagtag | 14c | 43,465 |  |
|  | gtggtggctc | 14d | 10,640 |  |
|  | taataagccc | 14e | 102,981 |  |
| cell #15 | agtagccagg | 15a | 31,383 | 207,870 |
|  | ccgcacgtca | 15b | 4,834 |  |
|  | gaaacgtatt | 15c | 6,717 |  |
|  | gatttggcta | 15d | 35,335 |  |
|  | taatctatct | 15e | 65,235 |  |
|  | tctcatcgaa | 15f | 23,515 |  |
|  | ttgcatctgc | 15g | 40,851 |  |
| control sample 1 | aatttattaa | Ca | 158,066 | 578,596 |
|  | atggagactg | Cb | 116,995 |  |
|  | gaccgctccc | Cc | 109,318 |  |
|  | gatatgcacc | Cd | 68,531 |  |
|  | gtacaagagg | Ce | 125,686 |  |
| control sample 2 | aatttattaa | Ca | 138,889 | 487,573 |
|  | atggagactg | Cb | 91,732 |  |
|  | gaccgctccc | Cc | 83,482 |  |
|  | gatatgcacc | Cd | 64,784 |  |
|  | gtacaagagg | Ce | 108,686 |  |
