# Supplementary material for: Viruses Roll the Dice: The Stochastic Behavior of Viral Genome Molecules Accelerates Viral Adaptation at the Cell and Tissue Levels
Source: PLoS Biol. 2015 Mar 17;13(3):e1002094. doi: 10.1371/journal.pbio.1002094 (PMC4364534; doi:10.1371/journal.pbio.1002094)
Supplement: S2 Table — Using the simulation model for cell infection, inoculations of 10,000 cells were simulated at different E values and fixed values for R, p, and d (R = 3 × 10^4^, p = 3 × 10^–10^, and d = 1 × 10^–2^). The expected numbers of infected (founder number ≥ 1) and uninfected (founder number = 0) cells out of the 10,000 cells are shown. (DOC) [file pbio.1002094.s024.doc]

**S2 Table. Expected numbers of infected and uninfected cells at different *E*** values

| *E* | # of infected cells out of 10,000 cells (founder number ≥ 1) | # of uninfected cells out of 10,000 cells (founder number = 0) |
| --- | --- | --- |
| 1 | 9 | 9,991 |
| 2 | 21 | 9,979 |
| 5 | 44 | 9,956 |
| 10 | 102 | 9,898 |
| 20 | 189 | 9,811 |
| 50 | 420 | 9,580 |
| 100 | 811 | 9,189 |
| 200 | 1,632 | 8,368 |
| 500 | 3,623 | 6,377 |
| 1,000 | 6,025 | 3,975 |
| 2,000 | 8,353 | 1,647 |
| 5,000 | 9,850 | 150 |
| 10,000 | 9,998 | 2 |
