# Supplementary material for: Viruses Roll the Dice: The Stochastic Behavior of Viral Genome Molecules Accelerates Viral Adaptation at the Cell and Tissue Levels
Source: PLoS Biol. 2015 Mar 17;13(3):e1002094. doi: 10.1371/journal.pbio.1002094 (PMC4364534; doi:10.1371/journal.pbio.1002094)
Supplement: S3 Table — (DOC) [file pbio.1002094.s025.doc]

**S3 Table.** The oligo DNA fragments used in this study.

| Name | Sequence | Details |
| --- | --- | --- |
| To14 | 5′-CTAA*GGTAACC*NNNNNNNNNNTAGGTGCTGAAATATAAAG-3′ | The oligo DNA fragment used to construct a cDNA library of virus variants tagged with random 10 nucleotide sequences. Italicized letters show the *Bst*EII restriction site. N indicates random nucleotides incorporated into the oligo DNAs, and underlined letters denote the sequence that anneals to To15. |
| To15 | 5′-CTCC*GCGCGC*TCCAAGACACTACCCTTCGATTTAAGTGGAGGGAAAAACACTGTACGTTATCGTACGTACCACGTGTGTTTTAGAAACACAAACTTTATATTTCAGCACCTA-3′ | Another oligo DNA fragment used to construct the cDNA library of virus variants tagged with random 10 nucleotide sequences. Italicized letters indicate the *Bss*HII restriction site, and underlined letters denote the sequence that anneals to To14. |
| To17 | 5′-GCGTTCAACTAGCAGAC-3′ | Forward primer used for RT-PCR. |
| To18 | 5′-TACGTGCCTACGGACAT-3′ | Reverse primer used for RT-PCR. |
| TagYF | 5′-XYZTGAACTGTACAACTAAGGTA-3′ | The forward primer sets used for nested PCR. XYZ indicates the 3–5 nucleotide tag sequences used to identify each cell sample. |
| TagGR | 5′-XYZACTTTATATTTCAGCACCTA-3’ | The reverse primer sets used for nested PCR. XYZ indicates the 3–5 nucleotide tag sequences used to identify each cell sample. |
