# Supplementary material for: Viruses Roll the Dice: The Stochastic Behavior of Viral Genome Molecules Accelerates Viral Adaptation at the Cell and Tissue Levels
Source: PLoS Biol. 2015 Mar 17;13(3):e1002094. doi: 10.1371/journal.pbio.1002094 (PMC4364534; doi:10.1371/journal.pbio.1002094)
Supplement: S4 Table — (DOC) [file pbio.1002094.s026.doc]

**S4 Table. *N*_e_-decreasing effect of SIPA at different founder numbers**.

| Founder number (*k*) | Estimated *α*(*^k^*) () | *k*+1 | *N*_e_, including the effect of SIPA () | *N*_e_-decreasing effect () |
| --- | --- | --- | --- | --- |
| 1 | NA | NA | 1 | NA |
| 2 | 1.00 ± 0.11 | 3.00 | 1.50 | 0.75 |
| 3 | 1.13 ± 0.07 | 4.40 | 2.06 | 0.69 |
| 4 | 1.09 ± 0.05 | 5.35 | 2.56 | 0.64 |
| 5 | 1.13 ± 0.06 | 6.63 | 3.12 | 0.62 |
| 6 | 0.99 ± 0.05 | 6.96 | 3.49 | 0.58 |
| 7 | 1.11 ± 0.07 | 8.80 | 4.16 | 0.59 |
| 8 | 1.14 ± 0.07 | 10.08 | 4.72 | 0.59 |
| 9 | 1.03 ± 0.11 | 10.28 | 5.06 | 0.56 |
| 10 | 1.18 ± 0.14 | 12.78 | 5.87 | 0.59 |
| 11 | 1.31 ± 0.52 | 15.42 | 6.67 | 0.61 |
| 12 | 2.27 ± 0.90 | 28.19 | 8.63 | 0.72 |
