# Supplementary material for: Viruses Roll the Dice: The Stochastic Behavior of Viral Genome Molecules Accelerates Viral Adaptation at the Cell and Tissue Levels
Source: PLoS Biol. 2015 Mar 17;13(3):e1002094. doi: 10.1371/journal.pbio.1002094 (PMC4364534; doi:10.1371/journal.pbio.1002094)
Supplement: S1 Text — The authors recommend the interested readers try this script. (DOC) [file pbio.1002094.s027.doc]

**S1 Text. An R script used to generate Fig 3ABC.**

# parameter settings

E <- 5*10^3

R <- 3*10^4

p <- 3*10^-10

d <- 1*10^-2

# initial settings

table <- matrix(rep(0,E*3),nrow=E)

table[,1] <- c(1:E) # ID for each introduced RNA

table[,2] <- c(rep(1,E)) # number of genomic RNA

table[,3] <- c(rep(0,E)) # number of RC

RCO <- R

alive <- E

nsum <- E

t <- 0

# graphics settings

letter <- c("8","9","A","B","C","D","E","F")

nresult <- matrix(rep("NA",600),nrow=2)

rresult <- matrix(rep("NA",600),nrow=2)

# main body of simulation

while (RCO > 0) {

if (nsum == 0) break

## showing graphics by every 100 unit time

if (t%%100 == 0){

nsum <- sum(table[,2])

colorn <- c(rep(0,alive))

idn <- c(rep(0,alive))

for (k in 1:alive){

colorn[k] <- as.character(paste("#",letter[1+table[k,1]%%8],"0",letter[1+table[k,1]%%7],"0",letter[1+table[k,1]%%6],"0",sep=""))

if (table[k,2]>1){

idn[k] <- table[k,1]

}else{

idn[k] <- ""

}

}

resultn <- table[,2]

matrixn1 <- rbind(resultn,idn,colorn)

matrixn2 <- matrix(c(as.character(matrixn1)),nrow=3)

if (RCO > 0){

idr <- c("open sites",idn)

resultr <- c(RCO,table[,3])

colorr <- c("#FFFFFF",colorn)

}else{

idr <- idn

resultr <- table[,3]

colorr <- colorn

}

matrixr1 <- rbind(resultr,idr,colorr)

matrixr2 <- matrix(c(as.character(matrixr1)),nrow=3)

if (RCO < R){

resultrr <- table[,3]

matrixrr1 <- rbind(resultrr,idn,colorn)

matrixrr2 <- matrix(c(as.character(matrixrr1)),nrow=3)

}else{

matrixrr2 <- matrix(c(1,"no RC","#FFFFFF"),nrow=3)

}

t1 <- t/100+1

nresult[1,t1] <- t

nresult[2,t1] <- nsum

rresult[1,t1] <- t

rresult[2,t1] <- R-RCO

x1 <- na.omit(nresult[1,])

y1 <- na.omit(nresult[2,])

x2 <- rresult[1,]

y2 <- rresult[2,]

par(mfrow=c(2,3))

pie(as.numeric(matrixn2[1,]),labels=matrixn2[2,],col=matrixn2[3,],clockwise=TRUE,main=paste("genomic RNA: ",nsum,sep=""),radius=sqrt(nsum/R*d))

pie(as.numeric(matrixr2[1,]),labels=matrixr2[2,],col=matrixr2[3,],clockwise=TRUE,main=paste("RC: ",R-RCO,"\n open site: ",RCO,sep=""))

pie(as.numeric(matrixrr2[1,]),labels=matrixrr2[2,],col=matrixrr2[3,],clockwise=TRUE,main=paste("RC proportion",sep=""))

plot(x1,y1,xlim=c(0,30000),ylim=c(0,R/d*6/5),col="red",xlab="t",ylab="genomic RNA")

plot(x2,y2,xlim=c(0,30000),ylim=c(0,R*6/5),col="blue",xlab="t",ylab="RC")

plot(0,0,xlim=c(0,1),ylim=c(0,1),col="white",xlab="",ylab="",axes=F,bty="n")

text(0.5,0.5,labels=paste("t = ",t),cex=2)

}else{

}

## genomic RNA degradation and synthesis

for (j in 1:alive) {

D[j] <- rbinom(1,table[j,2],d)

table[j,2] <- table[j,2]-D[j]+1*table[j,3]

}

nsum <- sum(table[,2])

## RC formation

if (nsum >0) {

irc <- rbinom(1,RCO,min(c(1,nsum*p)))

RCO <- RCO-irc

sr <- sample(1:alive,irc,replace=TRUE,prob=table[,2])

fr <- as.vector(table(factor(sr,levels=1:alive)))

table[,3] <- table[,3]+fr

} else {

}

## removing extinct ones from simulation procedure

if (prod(table[,2]) == 0){

table <- na.omit(t(rbind(table[,1],replace(table[,2],which(table[,2]==0),NA),table[,3])))

}else{

}

alive <- nrow(table)

t <- t+1

}

# showing graphics at the end of the simulation

nsum <- sum(table[,2])

colorn <- c(rep(0,alive))

idn <- c(rep(0,alive))

for (k in 1:alive){

colorn[k] <- as.character(paste("#",letter[1+table[k,1]%%8],"0",letter[1+table[k,1]%%7],"0",letter[1+table[k,1]%%6],"0",sep=""))

if (table[k,2]>1){

idn[k] <- table[k,1]

}else{

idn[k] <- ""

}

}

resultn <- table[,2]

matrixn1 <- rbind(resultn,idn,colorn)

matrixn2 <- matrix(c(as.character(matrixn1)),nrow=3)

if (RCO > 0){

idr <- c("open sites",idn)

resultr <- c(RCO,table[,3])

colorr <- c("#FFFFFF",colorn)

}else{

idr <- idn

resultr <- table[,3]

colorr <- colorn

}

matrixr1 <- rbind(resultr,idr,colorr)

matrixr2 <- matrix(c(as.character(matrixr1)),nrow=3)

if (RCO < R){

resultrr <- table[,3]

matrixrr1 <- rbind(resultrr,idn,colorn)

matrixrr2 <- matrix(c(as.character(matrixrr1)),nrow=3)

}else{

matrixrr2 <- matrix(c(1,"no RC","#FFFFFF"),nrow=3)

}

par(mfrow=c(2,3))

pie(as.numeric(matrixn2[1,]),labels=matrixn2[2,],col=matrixn2[3,],clockwise=TRUE,main=paste("genomic RNA: ",nsum,sep=""),radius=sqrt(nsum/R*d))

pie(as.numeric(matrixr2[1,]),labels=matrixr2[2,],col=matrixr2[3,],clockwise=TRUE,main=paste("RC: ",R-RCO,"\n open site: ",RCO,sep=""))

pie(as.numeric(matrixrr2[1,]),labels=matrixrr2[2,],col=matrixrr2[3,],clockwise=TRUE,main=paste("RC proportion",sep=""))

plot(x1,y1,xlim=c(0,30000),ylim=c(0,R/d*6/5),col="red",xlab="t",ylab="genomic RNA")

plot(x2,y2,xlim=c(0,30000),ylim=c(0,R*6/5),col="blue",xlab="t",ylab="RC")

plot(0,0,xlim=c(0,1),ylim=c(0,1),col="white",xlab="",ylab="",axes=F,bty="n")

text(0.5,0.5,labels=paste("t = ",t),cex=2)
