# Supplementary material for: Viruses Roll the Dice: The Stochastic Behavior of Viral Genome Molecules Accelerates Viral Adaptation at the Cell and Tissue Levels
Source: PLoS Biol. 2015 Mar 17;13(3):e1002094. doi: 10.1371/journal.pbio.1002094 (PMC4364534; doi:10.1371/journal.pbio.1002094)
Supplement: S2 Text — (DOC) [file pbio.1002094.s028.doc]

**S2 Text. An R script used to obtain the data for S3 Fig, S9 Fig, and S12 Fig.**

#R script for obtaining the simulation results summarized in S3 Fig, S9 Fig and S12 Fig.

#This script generates many output files in CSV format.

#This simulation may require a couple of days.

#The simulation results obtained by the authors are shown in S1 Data.

factors <- matrix(c(

0,4,-11,6,

0,4,-10,5,

0,4,-9,4,

0,4,-8,3,

0,4,-7,2,

0,4,-6,1,

1,4,-11,5,

1,4,-10,4,

1,4,-9,3,

1,4,-8,2,

1,4,-7,1,

2,4,-11,4,

2,4,-10,3,

2,4,-9,2,

2,4,-8,1,

3,1,-11,6,

3,1,-10,5,

3,1,-9,4,

3,1,-8,3,

3,1,-7,2,

3,1,-6,1,

3,2,-11,5,

3,2,-10,4,

3,2,-9,3,

3,2,-8,2,

3,2,-7,1,

3,3,-11,4,

3,3,-10,3,

3,3,-9,2,

3,3,-8,1,

3,4,-11,3,

3,4,-10,2,

3,4,-9,1,

3,5,-11,2,

3,5,-10,1,

3,6,-11,1,

4,4,-11,2,

4,4,-10,1,

5,4,-11,1

),ncol=39)

for (s in 1:39){

# parameter settings

Ef <- factors[1,s]

Rf <- factors[2,s]

pf <- factors[3,s]

df <- factors[4,s]

E <- 5*10^Ef

R <- 3*10^Rf

p <- 3*10^pf

d <- 1*10^(-df)

fname <- paste("cycles-E5",Ef,"R3",Rf,"p3",pf,"d1-",df,".csv",sep="")

cells <- 100

cmax <- 50

result <- matrix(rep(0,cmax*cells),nrow=cells)

resultac <- c(rep(0,cells))

c <- 1

while (c < (cells+1)){

# initial settings

t <- 1

table <- matrix(rep(0,(cmax+1)*3),nrow=3)

table[1,] <- c(0:cmax) # number of cycles

table[3,1] <- E

RCO <- R

nsum <- E

D <- c(rep(0,1+cmax))

# main body of simulation

while (RCO > 0) {

if (nsum == 0) break

for (j in 1:(cmax+1)) {

D[j] <- rbinom(1,table[3,j],d)

table[3,j] <- table[3,j]-D[j]+1*table[2,j]

}

nsum <- sum(table[3,])

if (nsum > 0) {

irc <- rbinom(1,RCO,min(c(1,nsum*p)))

RCO <- RCO-irc

sr <- sample(1:(1+cmax),irc,replace=TRUE,prob=table[3,])

fr <- as.vector(table(factor(sr,levels=1:(1+cmax))))

table[2,] <- table[2,]+c(0,fr[1:cmax])

} else {

}

t <- t+1

}

if (nsum > 0){

result[c,] <- table[2,2:(cmax+1)]

par(mfrow=c(1,1))

plot(s,c,ylim=c(0,cells))

c <- c+1

}else{

}

gc()

gc()

}

write.csv(result,file=fname)

}
