# Supplementary material for: Viruses Roll the Dice: The Stochastic Behavior of Viral Genome Molecules Accelerates Viral Adaptation at the Cell and Tissue Levels
Source: PLoS Biol. 2015 Mar 17;13(3):e1002094. doi: 10.1371/journal.pbio.1002094 (PMC4364534; doi:10.1371/journal.pbio.1002094)
Supplement: S3 Text — (DOC) [file pbio.1002094.s029.doc]

**S3 Text. An R script used to obtain the data for Fig 3D–3F, S7 Fig, and S10 Fig.**

#R script for obtaining the simulation results summarized in Fig 3D–3F, S7 Fig, and S10 Fig.

#This script generates many output files in CSV format.

#The simulation may take a long time using usual PC, typically days to a couple of weeks.

#The simulation results obtained by the authors are shown in S2 Data.

factors <- matrix(c(

0,4,-11,6,

0,4,-10,5,

0,4,-9,4,

0,4,-8,3,

0,4,-7,2,

0,4,-6,1,

1,4,-11,5,

1,4,-10,4,

1,4,-9,3,

1,4,-8,2,

1,4,-7,1,

2,4,-11,4,

2,4,-10,3,

2,4,-9,2,

2,4,-8,1,

3,1,-11,6,

3,1,-10,5,

3,1,-9,4,

3,1,-8,3,

3,1,-7,2,

3,1,-6,1,

3,2,-11,5,

3,2,-10,4,

3,2,-9,3,

3,2,-8,2,

3,2,-7,1,

3,3,-11,4,

3,3,-10,3,

3,3,-9,2,

3,3,-8,1,

3,4,-11,3,

3,4,-10,2,

3,4,-9,1,

3,5,-11,2,

3,5,-10,1,

3,6,-11,1,

4,4,-11,2,

4,4,-10,1,

5,4,-11,1

),ncol=39)

for (s in 1:39){

# parameter settings

Ef <- factors[1,s]

Rf <- factors[2,s]

pf <- factors[3,s]

df <- factors[4,s]

e <- 5*10^Ef

R <- 3*10^Rf

p <- 3*10^pf

d <- 1*10^(-df)

# file names saved

fname <- paste("shannon-E5",Ef,"R3",Rf,"p3",pf,"d1-",df,".csv",sep="")

cells <- 1000

resultas <- matrix(rep(0,2*cells),nrow=cells)

for (c in 1:cells){

# initial status

t <- 1

table <- matrix(rep(0,e*3),nrow=e)

table[,1] <- c(1:e) #ID for vRNA lines (each inoculated vRNAs and their progenies)

table[,2] <- c(rep(1,e)) #number of vRNA

table[,3] <- c(rep(0,e)) #number of RC

RCO <- R #number of open sites for RC formation

alive <- e #number of vRNA lines that have at least one vRNA

nsum <- e #total number of vRNAs

# main body of simulation

while (alive > 10 && RCO > 0) {

if (nsum == 0) break

D <- rbinom(c(rep(1,alive)),table[,2],c(rep(d,alive)))

table[,2] <- table[,2]-D+1*table[,3]

nsum <- sum(table[,2])

if (nsum > 0) {

irc <- rbinom(1,RCO,min(c(1,nsum*p)))

RCO <- RCO-irc

sr <- sample(1:alive,irc,replace=TRUE,prob=table[,2])

fr <- as.vector(table(factor(sr,levels=1:alive)))

table[,3] <- table[,3]+fr

} else {

}

if (prod(table[,2]) == 0){

table <- na.omit(t(rbind(table[,1],replace(table[,2],which(table[,2]==0),NA),table[,3])))

}else{

}

alive <- nrow(table)

t <- t+1

}

while (RCO > 0) {

if (nsum == 0) break

for (j in 1:alive) {

D[j] <- rbinom(1,table[j,2],d)

table[j,2] <- table[j,2]-D[j]+1*table[j,3]

}

nsum <- sum(table[,2])

if (nsum > 0) {

irc <- rbinom(1,RCO,min(c(1,nsum*p)))

RCO <- RCO-irc

sr <- sample(1:alive,irc,replace=TRUE,prob=table[,2])

fr <- as.vector(table(factor(sr,levels=1:alive)))

table[,3] <- table[,3]+fr

} else {

}

if (prod(table[,2]) == 0){

table <- na.omit(t(rbind(table[,1],replace(table[,2],which(table[,2]==0),NA),table[,3])))

}else{

}

alive <- nrow(table)

t <- t+1

}

if (prod(table[,3]) == 0){

tablef <- na.omit(t(rbind(table[,1],table[,2],replace(table[,3],which(table[,3]==0),NA))))

}else{

tablef <- table

}

founder <- nrow(tablef)

resultas[c,1] <- founder

nsumf <- sum(tablef[,2])

if (founder == 0){

result[c,] <- c(rep(0,150))

}else{

rn <- tablef[,2]/nsumf

resultas[c,2] <- sum(-rn*log(rn))

}

# showing the progress of simulation

par(mfrow=c(1,1))

plot(c,s,xlim=c(0,cells),ylim=c(0,s))

gc()

gc()

}

write.csv(resultas,file=fname) #founder number and shannon entropy of vRNA accumulation in each cell

}
