# Supplementary material for: Viruses Roll the Dice: The Stochastic Behavior of Viral Genome Molecules Accelerates Viral Adaptation at the Cell and Tissue Levels
Source: PLoS Biol. 2015 Mar 17;13(3):e1002094. doi: 10.1371/journal.pbio.1002094 (PMC4364534; doi:10.1371/journal.pbio.1002094)
Supplement: S4 Text — (DOC) [file pbio.1002094.s030.doc]

**S4 Text. The effect of sampling errors in the analysis of sequence-tagged virus.**

To assess the effect of sampling errors in the analysis of sequence-tagged virus using GAIIx, we carried out simple simulations using R software. When a random sampling of 4 × 10^4^ genomes from an equal (1:1:1:1) mixture of four differently-tagged viral genomes was simulated, the expected detection frequencies were 9,927, 10,017, 10,102, and 9,954 times for each of the genotypes. (The R script is shown below as Script #1). Comparing with the ideal detection frequency of 10,000 each, this result indicates that the effect of sampling error is only less than ~1% for each genotype (<~100 of 10,000), and only less than ~0.25% of total (<~100 of 40,000). A random sampling of 4 × 10^4^ genomes from a 29:8:2:1 mixture (Script #2; ideal frequencies of 29,000, 8,000, 2,000, and 1,000) expected detection frequencies of 28,960, 7,999, 2,006, and 1,035 for each, suggesting up to ~4% of sampling errors for each [35 (1,035-1,000) of 1,000 for the first genotype], up to ~0.1% of the total [40 (29,000-28,960) of 40,000]. Thus, we concluded that the effect of sampling errors is limited.

Script #1:

rmultinom(1,size=40000,prob=c(1,1,1,1))

Script #2:

rmultinom(1,size=40000,prob=c(29,8,2,1))
