# Supplementary material for: Viruses Roll the Dice: The Stochastic Behavior of Viral Genome Molecules Accelerates Viral Adaptation at the Cell and Tissue Levels
Source: PLoS Biol. 2015 Mar 17;13(3):e1002094. doi: 10.1371/journal.pbio.1002094 (PMC4364534; doi:10.1371/journal.pbio.1002094)
Supplement: S5 Text — (DOC) [file pbio.1002094.s031.doc]

**S5 Text. Quantification of the variance in progeny accumulation.**

The variance in the observed detection frequencies of the tag sequences was quantified by calculating effect size *ω* from a hypothetical equal detection as follows:

$\omega=\sqrt{\frac{\chi^{2}}{f_{Total}}}$,

where

$$\chi^{2}=\sum\frac{(f_{Observed}-f_{Expected})^{2}}{f_{Expected}}$$

and *f_Total_*, *f_Observed_*, and *f_Expected_* are the total frequency of the tags, the observed frequency of each tag, and the expected frequency of each tag, respectively, under specific assumptions such as equal detection. If the observed frequencies are equal to the expected frequencies, *ω* = 0, whereas a larger variance gives a larger *ω*. Eleven of the 15 cell samples showed large effect (*ω* ≥ 0.5) from the hypothetical equal detection, suggesting that the detection frequencies are far from equal. Three cells had a middle to large effect (*ω =* 0.3–0.5), and one cell had small to middle effect (*ω* = 0.27; S5 Fig, panel B). Control experiments to assess the effect of experimental artifacts were done by using transcripts of five tomato mosaic virus (ToMV) variants carrying different sequence tags. The reverse transcription–polymerase chain reaction (RT-PCR) product of an equal mixture of these transcripts (control sample 1) and of total RNA extracted from 1 × 10^5^ cells at 24 hours postinoculation (hpi) with the transcript mixture (control sample 2) were analyzed in the same way as single-cell samples. The resulting effect size from “ideal” equal detection was small to medium for both samples (*ω* = 0.25 and 0.26, respectively). In addition, the effect size comparing control sample 2 (i.e., after infection) to control sample 1 (i.e., inoculum) was very small (*ω* = 0.07; S5 Fig, panel C). These results suggest that bias in detection might cause small to middle effects on the detection frequencies, and that differences in the accumulation ability of tagged viruses might give only a very small effect. Because most of the cells showed much larger effects than these controls, most of the variance in detection frequencies was not caused by biased accumulation ability or biased detection, but rather originated in stochastic processes during viral infections.
