# Supplementary material for: Viruses Roll the Dice: The Stochastic Behavior of Viral Genome Molecules Accelerates Viral Adaptation at the Cell and Tissue Levels
Source: PLoS Biol. 2015 Mar 17;13(3):e1002094. doi: 10.1371/journal.pbio.1002094 (PMC4364534; doi:10.1371/journal.pbio.1002094)
Supplement: S6 Text — (DOC) [file pbio.1002094.s032.doc]

**S6 Text. An R script used to obtain the data for Fig 5C and 5D.**

#R script for obtaining the simulation results summarized in Fig 5C and 5D.

#This script generates 9 output files in CSV format.

#The simulation results obtained by the authors are shown in S3 Data.

###### STEP1: obtaining the raw data for 1000 cell infection

factors <- c(3,4,-10,2)

# parameter settings

Ef <- factors[1]

Rf <- factors[2]

pf <- factors[3]

df <- factors[4]

e <- 5*10^Ef

R <- 3*10^Rf

p <- 3*10^pf

d <- 1*10^(-df)

cells <- 1000

result <- matrix(rep(0,150*cells),nrow=cells)

for (c in 1:cells){

# initial status

t <- 1

table <- matrix(rep(0,e*3),nrow=e)

table[,1] <- c(1:e) #ID for vRNA lines (each inoculated vRNAs and their progenies)

table[,2] <- c(rep(1,e)) #number of vRNA

table[,3] <- c(rep(0,e)) #number of RC

RCO <- R #number of open sites for RC formation

alive <- e #number of vRNA lines that have at least one vRNA

nsum <- e #total number of vRNAs

# main body of simulation

while (alive > 10 && RCO > 0) {

if (nsum == 0) break

D <- rbinom(c(rep(1,alive)),table[,2],c(rep(d,alive)))

table[,2] <- table[,2]-D+1*table[,3]

nsum <- sum(table[,2])

if (nsum > 0) {

irc <- rbinom(1,RCO,min(c(1,nsum*p)))

RCO <- RCO-irc

sr <- sample(1:alive,irc,replace=TRUE,prob=table[,2])

fr <- as.vector(table(factor(sr,levels=1:alive)))

table[,3] <- table[,3]+fr

} else {

}

if (prod(table[,2]) == 0){

table <- na.omit(t(rbind(table[,1],replace(table[,2],which(table[,2]==0),NA),table[,3])))

}else{

}

alive <- nrow(table)

t <- t+1

}

while (RCO > 0) {

if (nsum == 0) break

for (j in 1:alive) {

D[j] <- rbinom(1,table[j,2],d)

table[j,2] <- table[j,2]-D[j]+1*table[j,3]

}

nsum <- sum(table[,2])

if (nsum > 0) {

irc <- rbinom(1,RCO,min(c(1,nsum*p)))

RCO <- RCO-irc

sr <- sample(1:alive,irc,replace=TRUE,prob=table[,2])

fr <- as.vector(table(factor(sr,levels=1:alive)))

table[,3] <- table[,3]+fr

} else {

}

if (prod(table[,2]) == 0){

table <- na.omit(t(rbind(table[,1],replace(table[,2],which(table[,2]==0),NA),table[,3])))

}else{

}

alive <- nrow(table)

t <- t+1

}

if (prod(table[,3]) == 0){

tablef <- na.omit(t(rbind(table[,1],table[,2],replace(table[,3],which(table[,3]==0),NA))))

}else{

tablef <- table

}

founder <- nrow(tablef)

nsumf <- sum(tablef[,2])

if (founder == 0){

result[c,] <- c(rep(0,150))

}else{

rest <- 50-founder

result[c,] <- c(tablef[,1],rep(0,rest),tablef[,2],rep(0,rest),tablef[,3],rep(0,rest))

}

gc()

gc()

}

write.csv(result,file="1000raw.csv",row.names=F)

rtable <- result

###### STEP2: simulating exclusion of defective variants in different conditions

#######condition1

tablen <- rtable[1:1000,51:65]

init <- c(0.2,0.8)

cells <- 1000

rep <- 10

results <- NULL

for (i in 1:rep){

t <- 1

tmax <- 20

result <- init[1]

tabler <- t(matrix(c(rep(init,cells)),ncol=cells))

for (t in 1:tmax){

for (c in 1:cells){

line <- ceiling(runif(1,0,cells))

sum1 <- sum(tablen[line,]*rbinom(15,1,tabler[c,1])) # adaptive genome

sum2 <- sum(tablen[line,])-sum1 # defective genome

sum3 <- sum1+sum2 # sum

if (sum3 == 0){

tabler[c,] <- c(NA,NA)

}else{

r1 <- round(sum1/sum3,3)

tabler[c,] <- c(r1,1-r1)

}

}

tabler <- na.omit(tabler)

alive <- nrow(tabler)

result <- c(result,mean(tabler[,1]))

for (c in 1:alive){

d <- rbinom(1,1,tabler[c,1])

if (d == 0){

tabler[c,] <- c(NA,NA)

}else{

}

}

tabler <- na.omit(tabler)

alive <- nrow(tabler)

if (alive < cells){

dupl <- sample(1:alive,(cells-alive),replace=T)

tabler <- rbind(tabler,tabler[dupl,])

}else{

}

t <- t+1

}

results <- rbind(results,result)

}

write.csv(results,file="trans-condition1.csv")

#######condition2

tablen <- rtable[1:1000,51:65]

tablen[tablen>0] <- 1

init <- c(0.2,0.8)

cells <- 1000

rep <- 10

results <- NULL

for (i in 1:rep){

t <- 1

tmax <- 20

result <- init[1]

tabler <- t(matrix(c(rep(init,cells)),ncol=cells))

for (t in 1:tmax){

for (c in 1:cells){

line <- ceiling(runif(1,0,1000))

sum1 <- sum(tablen[line,]*rbinom(15,1,tabler[c,1])) # adaptive genome

sum2 <- sum(tablen[line,])-sum1 #defective genome

sum3 <- sum1+sum2 # sum

if (sum3 == 0){

tabler[c,] <- c(NA,NA)

}else{

r1 <- round(sum1/sum3,3)

tabler[c,] <- c(r1,1-r1)

}

}

tabler <- na.omit(tabler)

alive <- nrow(tabler)

result <- c(result,mean(tabler[,1]))

for (c in 1:alive){

d <- rbinom(1,1,tabler[c,1])

if (d == 0){

tabler[c,] <- c(NA,NA)

}else{

}

}

tabler <- na.omit(tabler)

alive <- nrow(tabler)

if (alive < cells){

dupl <- sample(1:alive,(cells-alive),replace=T)

tabler <- rbind(tabler,tabler[dupl,])

}else{

}

t <- t+1

}

results <- rbind(results,result)

}

write.csv(results,file="trans-condition2.csv")

#######condition3

tablen <- rtable[1:1000,51:65]

tablen[tablen>0] <- 1

sumt <- sum(tablen)

sn <- sumt%/%1000

nln <- sumt%%1000

tablen <- t(matrix(c(rep(c(rep(1,sn),rep(0,(15-sn))),1000-nln),rep(c(rep(1,sn+1),rep(0,(15-sn-1))),nln)),ncol=1000))

init <- c(0.2,0.8)

cells <- 1000

rep <- 10

results <- NULL

for (i in 1:rep){

t <- 1

tmax <- 20

result <- init[1]

tabler <- t(matrix(c(rep(init,cells)),ncol=cells))

for (t in 1:tmax){

for (c in 1:cells){

line <- ceiling(runif(1,0,1000))

sum1 <- sum(tablen[line,]*rbinom(15,1,tabler[c,1])) # adaptive genome

sum2 <- sum(tablen[line,])-sum1 #defective genome

sum3 <- sum1+sum2 # sum

if (sum3 == 0){

tabler[c,] <- c(NA,NA)

}else{

r1 <- round(sum1/sum3,3)

tabler[c,] <- c(r1,1-r1)

}

}

tabler <- na.omit(tabler)

alive <- nrow(tabler)

result <- c(result,mean(tabler[,1]))

for (c in 1:alive){

d <- rbinom(1,1,tabler[c,1])

if (d == 0){

tabler[c,] <- c(NA,NA)

}else{

}

}

tabler <- na.omit(tabler)

alive <- nrow(tabler)

if (alive < cells){

dupl <- sample(1:alive,(cells-alive),replace=T)

tabler <- rbind(tabler,tabler[dupl,])

}else{

}

t <- t+1

}

results <- rbind(results,result)

}

write.csv(results,file="trans-condition3.csv")

#######fixed founder number of 1

ffn <- 1

tablen <- t(matrix(c(rep(c(rep(1,ffn),rep(0,(15-ffn))),1000)),ncol=1000))

init <- c(0.2,0.8)

cells <- 1000

rep <- 10

results <- NULL

for (i in 1:rep){

t <- 1

tmax <- 20

result <- init[1]

tabler <- t(matrix(c(rep(init,cells)),ncol=cells))

for (t in 1:tmax){

for (c in 1:cells){

line <- ceiling(runif(1,0,1000))

sum1 <- sum(tablen[line,]*rbinom(15,1,tabler[c,1])) # adaptive genome

sum2 <- sum(tablen[line,])-sum1 #defective genome

sum3 <- sum1+sum2 # sum

if (sum3 == 0){

tabler[c,] <- c(NA,NA)

}else{

r1 <- round(sum1/sum3,3)

tabler[c,] <- c(r1,1-r1)

}

}

tabler <- na.omit(tabler)

alive <- nrow(tabler)

result <- c(result,mean(tabler[,1]))

for (c in 1:alive){

d <- rbinom(1,1,tabler[c,1])

if (d == 0){

tabler[c,] <- c(NA,NA)

}else{

}

}

tabler <- na.omit(tabler)

alive <- nrow(tabler)

if (alive < cells){

dupl <- sample(1:alive,(cells-alive),replace=T)

tabler <- rbind(tabler,tabler[dupl,])

}else{

}

t <- t+1

}

results <- rbind(results,result)

}

write.csv(results,file="trans-ffn1.csv")

#######fixed founder number of 2

ffn <- 2

tablen <- t(matrix(c(rep(c(rep(1,ffn),rep(0,(15-ffn))),1000)),ncol=1000))

init <- c(0.2,0.8)

cells <- 1000

rep <- 10

results <- NULL

for (i in 1:rep){

t <- 1

tmax <- 20

result <- init[1]

tabler <- t(matrix(c(rep(init,cells)),ncol=cells))

for (t in 1:tmax){

for (c in 1:cells){

line <- ceiling(runif(1,0,1000))

sum1 <- sum(tablen[line,]*rbinom(15,1,tabler[c,1])) # adaptive genome

sum2 <- sum(tablen[line,])-sum1 #defective genome

sum3 <- sum1+sum2 # sum

if (sum3 == 0){

tabler[c,] <- c(NA,NA)

}else{

r1 <- round(sum1/sum3,3)

tabler[c,] <- c(r1,1-r1)

}

}

tabler <- na.omit(tabler)

alive <- nrow(tabler)

result <- c(result,mean(tabler[,1]))

for (c in 1:alive){

d <- rbinom(1,1,tabler[c,1])

if (d == 0){

tabler[c,] <- c(NA,NA)

}else{

}

}

tabler <- na.omit(tabler)

alive <- nrow(tabler)

if (alive < cells){

dupl <- sample(1:alive,(cells-alive),replace=T)

tabler <- rbind(tabler,tabler[dupl,])

}else{

}

t <- t+1

}

results <- rbind(results,result)

}

write.csv(results,file="trans-ffn2.csv")

#######fixed founder number of 3

ffn <- 3

tablen <- t(matrix(c(rep(c(rep(1,ffn),rep(0,(15-ffn))),1000)),ncol=1000))

init <- c(0.2,0.8)

cells <- 1000

rep <- 10

results <- NULL

for (i in 1:rep){

t <- 1

tmax <- 20

result <- init[1]

tabler <- t(matrix(c(rep(init,cells)),ncol=cells))

for (t in 1:tmax){

for (c in 1:cells){

line <- ceiling(runif(1,0,1000))

sum1 <- sum(tablen[line,]*rbinom(15,1,tabler[c,1])) # adaptive genome

sum2 <- sum(tablen[line,])-sum1 #defective genome

sum3 <- sum1+sum2 # sum

if (sum3 == 0){

tabler[c,] <- c(NA,NA)

}else{

r1 <- round(sum1/sum3,3)

tabler[c,] <- c(r1,1-r1)

}

}

tabler <- na.omit(tabler)

alive <- nrow(tabler)

result <- c(result,mean(tabler[,1]))

for (c in 1:alive){

d <- rbinom(1,1,tabler[c,1])

if (d == 0){

tabler[c,] <- c(NA,NA)

}else{

}

}

tabler <- na.omit(tabler)

alive <- nrow(tabler)

if (alive < cells){

dupl <- sample(1:alive,(cells-alive),replace=T)

tabler <- rbind(tabler,tabler[dupl,])

}else{

}

t <- t+1

}

results <- rbind(results,result)

}

write.csv(results,file="trans-ffn3.csv")

#######fixed founder number of 4

ffn <- 4

tablen <- t(matrix(c(rep(c(rep(1,ffn),rep(0,(15-ffn))),1000)),ncol=1000))

init <- c(0.2,0.8)

cells <- 1000

rep <- 10

results <- NULL

for (i in 1:rep){

t <- 1

tmax <- 20

result <- init[1]

tabler <- t(matrix(c(rep(init,cells)),ncol=cells))

for (t in 1:tmax){

for (c in 1:cells){

line <- ceiling(runif(1,0,1000))

sum1 <- sum(tablen[line,]*rbinom(15,1,tabler[c,1])) # adaptive genome

sum2 <- sum(tablen[line,])-sum1 #defective genome

sum3 <- sum1+sum2 # sum

if (sum3 == 0){

tabler[c,] <- c(NA,NA)

}else{

r1 <- round(sum1/sum3,3)

tabler[c,] <- c(r1,1-r1)

}

}

tabler <- na.omit(tabler)

alive <- nrow(tabler)

result <- c(result,mean(tabler[,1]))

for (c in 1:alive){

d <- rbinom(1,1,tabler[c,1])

if (d == 0){

tabler[c,] <- c(NA,NA)

}else{

}

}

tabler <- na.omit(tabler)

alive <- nrow(tabler)

if (alive < cells){

dupl <- sample(1:alive,(cells-alive),replace=T)

tabler <- rbind(tabler,tabler[dupl,])

}else{

}

t <- t+1

}

results <- rbind(results,result)

}

write.csv(results,file="trans-ffn4.csv")

#######fixed founder number of 5

ffn <- 5

tablen <- t(matrix(c(rep(c(rep(1,ffn),rep(0,(15-ffn))),1000)),ncol=1000))

init <- c(0.2,0.8)

cells <- 1000

rep <- 10

results <- NULL

for (i in 1:rep){

t <- 1

tmax <- 20

result <- init[1]

tabler <- t(matrix(c(rep(init,cells)),ncol=cells))

for (t in 1:tmax){

for (c in 1:cells){

line <- ceiling(runif(1,0,1000))

sum1 <- sum(tablen[line,]*rbinom(15,1,tabler[c,1])) # adaptive genome

sum2 <- sum(tablen[line,])-sum1 #defective genome

sum3 <- sum1+sum2 # sum

if (sum3 == 0){

tabler[c,] <- c(NA,NA)

}else{

r1 <- round(sum1/sum3,3)

tabler[c,] <- c(r1,1-r1)

}

}

tabler <- na.omit(tabler)

alive <- nrow(tabler)

result <- c(result,mean(tabler[,1]))

for (c in 1:alive){

d <- rbinom(1,1,tabler[c,1])

if (d == 0){

tabler[c,] <- c(NA,NA)

}else{

}

}

tabler <- na.omit(tabler)

alive <- nrow(tabler)

if (alive < cells){

dupl <- sample(1:alive,(cells-alive),replace=T)

tabler <- rbind(tabler,tabler[dupl,])

}else{

}

t <- t+1

}

results <- rbind(results,result)

}

write.csv(results,file="trans-ffn5.csv")
