# Supplementary material for: Viruses Roll the Dice: The Stochastic Behavior of Viral Genome Molecules Accelerates Viral Adaptation at the Cell and Tissue Levels
Source: PLoS Biol. 2015 Mar 17;13(3):e1002094. doi: 10.1371/journal.pbio.1002094 (PMC4364534; doi:10.1371/journal.pbio.1002094)
Supplement: S7 Text — (DOC) [file pbio.1002094.s033.doc]

**S7 Text. The adaptation-enhancing effects of SVFN and SIPA: the effective population size.**

Preceding population genetics studies have shown that stochastic variation of population size and variance in reproductive success inflates the variance of allele frequencies, resulting in the decreased effective population size (*N*_e_) and the enhanced fixation [1,2,3,4,5,6,7]. This is consistent with our simulation results that the SVFN and SIPA enhanced separation of adaptive genomes from defective ones (i.e., the enhanced fixation in each lineage) and accelerated adaptation as a whole (Fig. 5). Interestingly, a simulation assuming SVFN and SIPA with a mean founder number of 4.34 (“condition 1” in Fig. 5CD) showed a rate of adaptation that was comparable to a simulation assuming a fixed founder number of 2 with no SIPA (i.e., “fixed founder number of 2” in Fig. 5D), suggesting that SVFN and SIPA decreased *N*_e_ from 4.34 to ~2. Similarly, the comparable rate of adaptations under “condition 2” and with a “fixed founder number of 3” (Fig. 5D) suggests that SVFN decreased *N*_e_ from 4.34 to ~3. We here analyze these *N*_e_-decreasing effects of SVFN and SIPA in a population genetics approach, in order to exhibit the relatedness of our simulation model to the population genetics studies.

**Effect of SVFN**

In the current study, SVFN indicates variation in the founder number among cells. This variation is interchangeable with the time-course variation in founder number that was analyzed previously [7]. Therefore, an effective population size assuming SVFN ($N_{e}^{SVFN}$) can be obtained by calculating the weighted harmonic mean of the founder number. In a uniform environment, a founder number (*k*) follows the Poisson distribution. We exclude infections by zero founders by calculating $N_{e}^{SVFN}$ as follows:

$N_{e}^{SVFN}=\frac{1}{\sum_{k=1}^{\infty} \frac{r_{k}}{k}}$.

Here, the relative frequency of *k* founder *r_k_* is

$r_{k}=\frac{1}{1-e^{-\lambda}}\cdot\frac{\lambda^{k}e^{-\lambda}}{k!}$,

where *λ* is the mean founder number, including infection by zero founders. Using *λ* = 4.34, $N_{e}^{SVFN}$= 3.31, which is consistent with the simulation results.

**Effects of SIPA at Different Founder Numbers**

In the current study, SIPA defines the phenomenon whereby a different amount of progenies accumulate in a cell from each single founder genome. First, we assumed that two founders have alleles *A* and *B* at an overall ratio of *p*:(1 – *p*). A small founder number causes variation in the ratio of the *A* allele in founders (*p_c_*) among cells, and SIPA causes further variation in the ratio of the *A* allele in their progenies ($p_{c}^{SIPA}$). Therefore, the overall variation after cell infections can be expressed as

$V\left[p_{c}^{SIPA}\right]=E\left[\left(p_{c}^{SIPA}-p\right)^{2}\right]\approx E\left[\left(p_{c}^{SIPA}-p_{c}\right)^{2}\right]+E\left[\left(p_{c}-p\right)^{2}\right]$. (S1)

Assuming that SIPA follows beta distribution beta(*α*,*β*) with shape parameters *α* and *β*, Equation S1 can be modified as follows:

$$V\left[p_{c}^{SIPA}\right]=\frac{E\left[p_{c}\left(1-p_{c}\right)\right]}{\alpha+\beta+1}+V\left[p_{c}\right]$$

$$=\frac{p-\left(p^{2}+V\left[p_{c}\right]\right)}{\alpha+\beta+1}+V\left[p_{c}\right]$$

$$=\frac{p\left(1-p\right)}{\alpha+\beta+1}+\left(1-\frac{1}{\alpha+\beta+1}\right)\cdot V\left[p_{c}\right]$$

$$=\frac{p\left(1-p\right)}{\alpha+\beta+1}+\left(1-\frac{1}{\alpha+\beta+1}\right)\cdot\frac{p\left(1-p\right)}{2}$$

$$=\frac{p\left(1-p\right)}{2}\left(1+\frac{1}{\alpha+\beta+1}\right)$$

Therefore, the effective population size (assuming that SIPA occurs) with a founder number *k =* 2 () is obtained using

. (S2)

By generalizing the beta distribution to the Dirichlet distribution Dir(*α*_1_, …, *α_k_*) with the concentration parameters *α*_1_, …, *α_k_*, calculation of the effective population size for *k* founders is possible. Considering that each founder begins replication from a single genome molecule, the concentration parameters should be equal among the founders. Defining the concentration parameter as *α*(*^k^*) ≡ *α*_1_ … = *α_k_*, Equation S2 is generalized as

. (S3)

We estimated *α*(*^k^*) for each founder number *k* based on the simulated accumulation levels summarized in Figure 3D using the maximum likelihood method. The estimates of *α*(*^k^*) ($\hat{\alpha}^{\left(k\right)}$) are summarized in S4 Table. Subsequently, was calculated using Equation S3 and is summarized in S4 Table. The *N*_e_-decreasing effects at different founder numbers were calculated by and are also shown in S4 Table.

**Combined Effects of SVFN and SIPA**

The effective population size assuming both SVFN and SIPA () can be obtained by calculating the weighted harmonic mean of based on S4 Table. The calculated was 2.35, consistent with the simulation results.

**References**

1. Felsenstein J (1971) Inbreeding and variance effective numbers in populations with overlapping generations. Genetics 68: 581-597.

2. Kimura M, Crow JF (1963) Measurement of effective population number. Evolution 17: 279-288.

3. Kimura M, Ohta T (1969) Average number of generations until fixation of a mutant gene in a finite population. Genetics 61: 763-771.

4. Nei M, Tajima F (1981) Genetic drift and estimation of effective population size. Genetics 98: 625-640.

5. Waples RS (1989) A generalized approach for estimating effective population size from temporal changes in allele frequency. Genetics 121: 379-391.

6. Wright S (1931) Evolution in Mendelian populations. Genetics 16: 97-159.

7. Wright S (1938) Size of population and breeding structure in relation to evolution. Science 87: 430-431.
