# Supplementary material for: Viruses Roll the Dice: The Stochastic Behavior of Viral Genome Molecules Accelerates Viral Adaptation at the Cell and Tissue Levels
Source: PLoS Biol. 2015 Mar 17;13(3):e1002094. doi: 10.1371/journal.pbio.1002094 (PMC4364534; doi:10.1371/journal.pbio.1002094)
Supplement: S8 Text — (DOC) [file pbio.1002094.s034.doc]

**S8 Text. An R script used to simulate co-inoculation of 1,000 cells with a wild-type virus and a variant that synthesizes viral genomic RNA at 50% efficiency to the wild-type virus.**

#R script for simulating 1000 cells inoculation with a 1:1 mixture of a wild-type virus and a variant with 50% efficiency of genomic RNA synthesis.

repr <- 0.5

cell <- 1000

Y <- c(rep(0,cell))

C <- c(rep(0,cell))

# parameter settings

E <- 5*10^3

d <- 1*10^-2

R <- 3*10^4

p <- 3*10^-10

Eyr <- 0.5 #proportion of wild-type (Y) in the introduced vRNAs.

for (c in 1:cell){

# initial settings

t <- 1

ta <- 1000

Ey <- round(E*Eyr)

Ec <- E-Ey

table <- matrix(rep(0,6),nrow=2)

table[,1] <- c(1,2)

table[,2] <- c(Ey,Ec)

table[,3] <- c(0,0)

RCO <- R

# main body of simulation

nsum <- E

while (RCO > 0 || ta >0) {

if (nsum == 0) break

D <- rbinom(c(1,1),table[,2],c(d,d))

G <- c(rbinom(1,table[1,3],repr),table[2,3])

table[,2] <- table[,2]-D+G

nsum <- sum(table[,2])

if (nsum > 0) {

irc <- rbinom(1,RCO,min(c(1,nsum*p)))

RCO <- RCO-irc

ircy <- rbinom(1,irc,table[1,2]/nsum)

ircc <- irc-ircy

table[1,3] <- table[1,3]+ircy

table[2,3] <- table[2,3]+ircc

} else {

}

if (RCO == 0) {

ta <- ta-1

}else{

}

t <- t+1

}

#showing the progress of simulation

par(mfrow=c(1,1))

plot(c,c)

Y[c] <- table[1,2]

C[c] <- table[2,2]

gc()

gc()

}

YC <- Y/C

#infected cell

ni <- length(which(YC>=0))

ni

#singly infected cell by the variant

nsv <- length(which(YC>=Inf))

nsv

#singly infected cell by the wild-type virus

nsw <- length(which(YC==0))

nsw

#co-infected cells

nc <- ni-nsv-nsw

nc

YC0 <- YC[which(YC>0)]

YC0i <- YC0[which(YC0<Inf)]

min(YC0i)

max(YC0i)

median(YC0i)
