# Supplementary material for: Viruses Roll the Dice: The Stochastic Behavior of Viral Genome Molecules Accelerates Viral Adaptation at the Cell and Tissue Levels
Source: PLoS Biol. 2015 Mar 17;13(3):e1002094. doi: 10.1371/journal.pbio.1002094 (PMC4364534; doi:10.1371/journal.pbio.1002094)
Supplement: S10 Text — (DOC) [file pbio.1002094.s036.doc]

**S10 Text. An R script used to generate Fig. 6C.**

#R script for Fig. 6C.

#Seed value is set in order to link the simulation results of this simulation and a simulation for Fig. 6A and 6B.

set.seed(3)

par(mfrow=c(1,1))

plot(0,0,type="n",ylim=c(0,1),xlim=c(0,20000),xlab="",ylab="")

for (c in 1:10){

# parameter settings

E <- 5*10^3

R <- 3*10^4

p <- 3*10^-10

d <- 1*10^-2

rep <- c(0.5,1)

# initial settings

table <- matrix(rep(0,6),nrow=2)

table[,1] <- c(1,2) # ID

table[,2] <- c(E/2,E/2) # number of genomic RNA

table[,3] <- c(0,0) # number of RC

RCO <- R

nsum <- E

t <- 0

# graphics settings

nresult <- matrix(rep(NA,400000),nrow=4)

rresult <- matrix(rep(NA,400000),nrow=4)

colorn <- c("green","magenta")

# main body of simulation

while (RCO > 0) {

if (nsum == 0) break

## showing graphics by every 100 unit time

if (t%%1 == 0){

t1 <- t+1

nresult[1,t1] <- t

nresult[2,t1] <- table[1,2]

nresult[3,t1] <- table[2,2]

if(table[1,2]+table[2,2]>0){

nresult[4,t1] <- table[1,2]/(table[1,2]+table[2,2])

}else{

nresult[4,t1] <- NA

}

rresult[1,t1] <- t

rresult[2,t1] <- table[1,3]

rresult[3,t1] <- table[2,3]

if(table[1,3]+table[2,3]>0){

rresult[4,t1] <- table[1,3]/(table[1,3]+table[2,3])

}else{

rresult[4,t1] <- NA

}

}else{

}

## genomic RNA degradation and synthesis

for (j in 1:2) {

D[j] <- rbinom(1,table[j,2],d)

G[j] <- rbinom(1,table[j,3],rep[j])

table[j,2] <- table[j,2]-D[j]+G[j]

}

nsum <- sum(table[,2])

## RC formation

if (nsum >0) {

irc <- rbinom(1,RCO,min(c(1,nsum*p)))

RCO <- RCO-irc

ircy <- rbinom(1,irc,table[1,2]/nsum)

ircc <- irc-ircy

table[1,3] <- table[1,3]+ircy

table[2,3] <- table[2,3]+ircc

} else {

}

t <- t+1

}

points(nresult[1,],nresult[4,],type="l",lwd="4",col=rainbow(11,start=(c-1)/11,alpha=0.5))

}
