# Supplementary material for: Viruses Roll the Dice: The Stochastic Behavior of Viral Genome Molecules Accelerates Viral Adaptation at the Cell and Tissue Levels
Source: PLoS Biol. 2015 Mar 17;13(3):e1002094. doi: 10.1371/journal.pbio.1002094 (PMC4364534; doi:10.1371/journal.pbio.1002094)
Supplement: S13 Text — (DOC) [file pbio.1002094.s039.doc]

**S13 Text. An R script used to obtain the data for S6 Fig.**

#R script for obtaining the simulation results summarized in S6 Fig.

#A parameter value for "rcf", which determine the relative efficiency of RC formation by a variant, need to be provided.

#The simulation results obtained by the authors are shown in S5 Data.

rcf <- 0.5

cell <- 100

Y <- c(rep(0,cell))

C <- c(rep(0,cell))

# parameter settings

E <- 5*10^3

d <- 1*10^-2

R <- 3*10^4

p <- 3*10^-10

Eyr <- 0.5 #proportion of wild-type (Y) in the introduced vRNAs.

for (c in 1:cell){

# initial settings

t <- 1

ta <- 1000

Ey <- round(E*Eyr)

Ec <- E-Ey

table <- matrix(rep(0,6),nrow=2)

table[,1] <- c(1,2)

table[,2] <- c(Ey,Ec)

table[,3] <- c(0,0)

RCO <- R

# main body of simulation

nsum <- E

while (RCO > 0 || ta >0) {

if (nsum == 0) break

D <- rbinom(c(1,1),table[,2],c(d,d))

G <- table[,3]

table[,2] <- table[,2]-D+G

nsum <- sum(table[,2])

nsumm <- sum(table[,2]*c(rcf,1))

if (nsum > 0) {

irc <- rbinom(1,RCO,min(c(1,p*nsumm)))

RCO <- RCO-irc

ircy <- rbinom(1,irc,table[1,2]*rcf/nsumm)

ircc <- irc-ircy

table[1,3] <- table[1,3]+ircy

table[2,3] <- table[2,3]+ircc

} else {

}

if (RCO == 0) {

ta <- ta-1

}else{

}

t <- t+1

}

plot(c,c)

Y[c] <- table[1,2]

C[c] <- table[2,2]

gc()

gc()

}

BSresult <- matrix(rep(0,cell*3),nrow=3)

for(b in 1:cell){

YT <- 0

CT <- 0

s <- sample(1:cell,cell,replace=T)

for (i in 1:cell){

YT <- YT+Y[s[i]]

CT <- CT+C[s[i]]

}

YC <- YT/CT

BSresult[1,b] <- YT

BSresult[2,b] <- CT

BSresult[3,b] <- YC

}

mean(BSresult[1,]) #number of the variant RNA in 1000 cells

sd(BSresult[1,]) #standard error caluculated by boot-strap analysis

mean(BSresult[2,]) #number of wild-type RNA in 1000 cells

sd(BSresult[2,]) #standard error caluculated by boot-strap analysis

mean(BSresult[3,]) #ratio of the variant to wild-type RNA

sd(BSresult[3,]) #standard error caluculated by boot-strap analysis
