# Supplementary material for: Viruses Roll the Dice: The Stochastic Behavior of Viral Genome Molecules Accelerates Viral Adaptation at the Cell and Tissue Levels
Source: PLoS Biol. 2015 Mar 17;13(3):e1002094. doi: 10.1371/journal.pbio.1002094 (PMC4364534; doi:10.1371/journal.pbio.1002094)
Supplement: S14 Text — (DOC) [file pbio.1002094.s040.doc]

**S14 Text. An R script used to obtain the data for S8 Fig and S11 Fig.**

#R script for obtaining the simulation results summarized in S8 Fig and S11 Fig.

#This script generates an output file in CSV format.

#This simulation may require a couple of days.

#The simulation results obtained by the authors are shown in S6 Data.

repr <- 0.5

cell <- 1000

factors <- matrix(c(

0,4,-11,6,

0,4,-10,5,

0,4,-9,4,

0,4,-8,3,

0,4,-7,2,

0,4,-6,1,

1,4,-11,5,

1,4,-10,4,

1,4,-9,3,

1,4,-8,2,

1,4,-7,1,

2,4,-11,4,

2,4,-10,3,

2,4,-9,2,

2,4,-8,1,

3,1,-11,6,

3,1,-10,5,

3,1,-9,4,

3,1,-8,3,

3,1,-7,2,

3,1,-6,1,

3,2,-11,5,

3,2,-10,4,

3,2,-9,3,

3,2,-8,2,

3,2,-7,1,

3,3,-11,4,

3,3,-10,3,

3,3,-9,2,

3,3,-8,1,

3,4,-11,3,

3,4,-10,2,

3,4,-9,1,

3,5,-11,2,

3,5,-10,1,

3,6,-11,1,

4,4,-11,2,

4,4,-10,1,

5,4,-11,1

),ncol=39)

results <- NULL

for (s in 1:39){

# parameter settings

Ef <- factors[1,s]

Rf <- factors[2,s]

pf <- factors[3,s]

df <- factors[4,s]

E <- 5*10^Ef

R <- 3*10^Rf

p <- 3*10^pf

d <- 1*10^(-df)

Y <- c(rep(0,cell))

C <- c(rep(0,cell))

c <- 1

while (c < (cell+1)){

# parameter settings

eyr <- 1/2

t <- 1

# initial settings

Niy <- round(E*eyr)

Nic <- E-Niy

Npy <- 0

Npc <- 0

Ry <- 0

Rc <- 0

RCO <- R

# main body of simulation

nsum <- E

while (RCO > 0) {

if (nsum == 0) break

Diy <- rbinom(1,Niy,d)

Dic <- rbinom(1,Nic,d)

Dpy <- rbinom(1,Npy,d)

Dpc <- rbinom(1,Npc,d)

Gy <- rbinom(1,Ry,repr)

Gc <- Rc

Niy <- Niy-Diy

Nic <- Nic-Dic

Npy <- Npy-Dpy+Gy

Npc <- Npc-Dpc+Gc

nsum <- Niy+Nic+Npy+Npc

if (nsum > 0) {

irc <- rbinom(1,RCO,min(c(1,nsum*p)))

RCO <- RCO-irc

ircy <- rbinom(1,irc,(Niy+Npy)/nsum)

ircc <- irc-ircy

Ry <- Ry+ircy

Rc <- Rc+ircc

} else {

}

t <- t+1

}

if (RCO == 0){

Y[c] <- Npy

C[c] <- Npc

}else{

}

gc()

gc()

c <- c+1

par(mfrow=c(1,1))

plot(c,s,xlim=c(0,cell))

}

BSresult <- matrix(rep(0,cell*3),nrow=3)

for(b in 1:cell){

YT <- 0

CT <- 0

s <- sample(1:cell,cell,replace=T)

YT <- sum(Y[s])

CT <- sum(C[s])

YC <- YT/CT

BSresult[1,b] <- YT

BSresult[2,b] <- CT

BSresult[3,b] <- YC

}

meansd <- c(Ef,Rf,pf,df,mean(BSresult[1,]),sd(BSresult[1,]),mean(BSresult[2,]),sd(BSresult[2,]),mean(BSresult[3,]),sd(BSresult[3,]))

results <- rbind(results,meansd)

}

write.csv(results,file="YC05.csv")
